# Synthesis of D-fructose-derived spirocyclic 2-substituted-2-oxazoline ribosides

**DOI:** 10.3762/bjoc.11.249

**Published:** 2015-11-24

**Authors:** Madhuri Vangala, Ganesh P Shinde

**Affiliations:** 1 Department of Chemistry, Indian Institute of Science Education and Research, Pune 411 008, India

**Keywords:** fructose, oxazoline, riboside, Ritter reaction, spiro

## Abstract

The TMSOTf-mediated synthesis of β-configured spirocyclic 2-substituted-2-oxazoline ribosides was achieved using a “Ritter-like” reaction in toluene through nucleophilic addition of electron-rich nitriles to the oxacarbenium ion intermediate of 1,2;3,4-di-*O*-isopropylidene-β-D-psicofuranose derivatives with concomitant intramolecular trapping of the C2 hydroxymethyl group on the electrophilic nitrilium carbon. These carbohydrate-derived spirooxazolines are stable and were obtained in good yield with high stereoselectivity due to the conformational rigidity imparted by the 3,4-isopropylidene group.

## Introduction

**Figure 1:**
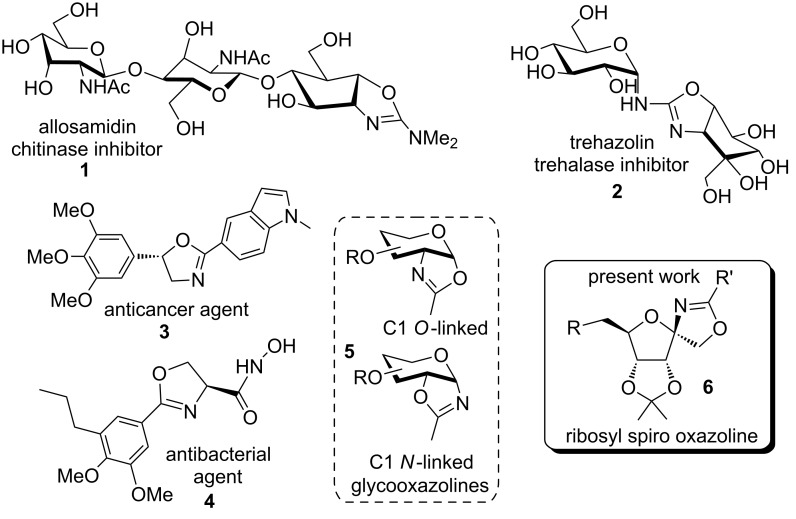
Some representative molecules having a 2-oxazoline moiety.

2-Oxazolines represent a unique class of 5-membered heterocyclic compounds with a wide range of applications spanning different chemistry disciplines. 2-Oxazoline derivatives are extensively studied due to their presence in numerous bioactive natural products [1–3] and synthetic drugs with anticancer, antibiotic, antidiabetic, and antifungal properties (Figure 1, **1–4**) [4–6]. The strong affinity of the nitrogen in oxazoline towards metal makes the chiral derivatives excellent catalysts for asymmetrical transformations [7–10]. The amphiphilicity and biocompatibility of poly-2-substituted-2-oxazolines gained them recognition as biomaterials for polymer therapeutics [11–13]. Traditionally, oxazolines are used as protecting groups in organic synthesis [14]. Several efficient methods for the construction of the 2-oxazoline functionality are reported in the literature from alkenes, carboxylic acid derivatives, nitriles [15–19], etc. In carbohydrate chemistry, they are versatile intermediates in the synthesis of *N*-linked glycoproteins [20–22].

In the context of carbohydrate chemistry, there are two possible regioisomers for glycooxazolines. Firstly, the C1 *O*-linked 1,2-glycooxazolines (Figure 1, **5**) are formed by intramolecular nucleophilic substitution at the anomeric center by vicinal participation of the C2 acylamino group. These are highly reactive and favor the formation of 1,2-*trans*-glycosides in a diastereoselective fashion [23–26]. Secondly, the C1 *N*-linked 1,2-glycooxazolines (Figure 1, **5**) are derived from either glycosyl azides via intramolecular condensation with a vicinal ester group in the presence of triphenylphosphine [27] or from the reaction of D-glucals with *N*-iodosuccinimide and amides [28–29]. Alternatively, **5** can be derived by a Ritter-like [30–31] transformation, involving trapping of the oxacarbenium-ion intermediate by a nitrile and subsequent intramolecular nucleophilic attack of the vicinal C2 ether or a free hydroxy group [32–34]. Such glycooxazolines are exploited for the generation of *N*-glycan structures [35]. In *O*-glycosylation reactions, an oxacarbenium-ion intermediate interacts with nitrile solvents, providing a transient α,β-glycosyl nitrilium species that could produce an oxazolinium intermediate through the participation of the vicinal oxygen atom. In 1981, Pavia and co-workers proposed the formation of uncharacterized oxazolinium from glycosyl hemiacetal in acetonitrile that upon acid-catalyzed hydrolysis gave glucosylamines [36]. In 1991, Danishefsky and co-workers synthesized a C1 *N*-linked glycooxazoline from a 1,2-anhydropyranose system in dry acetonitrile and zinc chloride as a Lewis acid [32]. Though the fused oxazolines were fully characterized, the compounds were eventually found to be unstable. Later in 2004, García Fernández and co-workers elegantly showed the formation of fused and spiroglycooxazolines from D-fructose [37]. More recently, Mong and co-workers synthesized fused glucopyranose oxazolines in nitrile solvents from thioglycoside donors and applied them to the synthesis of α(1→5)-arabinan [38]. Thus, the exploration of carbohydrate-based oxazolines [39–40] has been limited to serve as glycosyl donors or as intermediates for *N*-glycan synthesis. Although, a few pyranose-based oxazoline frameworks are known [41], the corresponding furanoid spiro [42] 2-oxazolines [43] are limited, to the best of our knowledge. With our interest in the area of glycopeptides [44] and carbohydrates [45], and owing to the importance of ribosides in general and spiroribofuranoses [46] in particular for drug discovery, in this paper, we report the synthesis of spiro 2-substituted-2-oxazoline ribosides utilizing a Ritter-like reaction having the general structure **6** (Figure 1).

## Results and Discussion

**Scheme 1:**
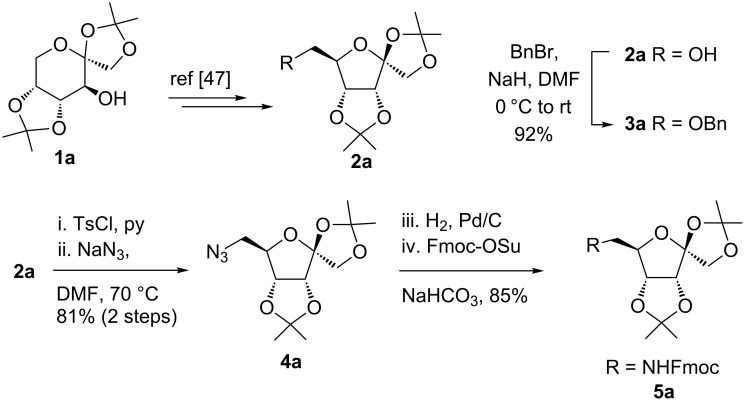
Synthesis of **3a**, **5a** from 1,2;3,4-di-*O*-isopropylidene-β-D-fructopyranose (**2a**).

García Fernández et al. isolated the fused and spiroglycooxazolines by activating 1,2;4,5-di-*O*-isopropylidene-β-D-fructopyranose and 3,4,6-tri-*O*-benzyl or benzoyl-protected 1,2-*O*-isopropylidene-β-D-fructofuranose derivatives with triflic acid (1.5 equiv) using nitriles (10 equiv) at −20 °C to rt in DCM [37]. Although fructopyranoses were shown to react with acetonitrile, propionitrile and benzonitrile, only one example of the corresponding fructofuranose with acetonitrile was shown, illustrating the instability of spirooxazolines in comparison with fused oxazolines [37]. In our work, the activation of the β-D-psicofuranose derivative **5a** with TMSOTf in acetonitrile exclusively resulted in the formation of spirooxazoline **6a**. The stability of this compound inspired us to explore the scope of the synthesis and isolation of spirooxazolines. Thus, the required key intermediate D-psicofuranose **2a** was synthesized in four steps from the readily available starting material D-fructose following the literature procedure [42,47]. The C6–OH group of D-psicofuranose was benzylated using BnBr and NaH in DMF to afford **3a** (Scheme 1). Further, the C6-tosyl intermediate was converted into 6-azido-6-deoxy-1,2;3,4-di-*O*-isopropylidene-β-D-psicofuranose (**4a**) by a nucleophilic displacement of the C6-*O*-tosylate with NaN_3_ in DMF at 70 °C [48]. The spectral data and the specific rotation values of **2a** and **4a** are in accordance with the previously reported data [42,47–48]. In the next step, hydrogenation of the azide with 10% Pd/C in EtOAc afforded the primary amine, which was protected using Fmoc-OSu/NaHCO_3_ in THF/H_2_O to afford **5a**. Finally, the C6–OBn- and C6–NHFmoc-protected compounds **3a** and **5a** were utilized to check the substrate feasibility in the formation of spirooxazolines.

**Scheme 2:**
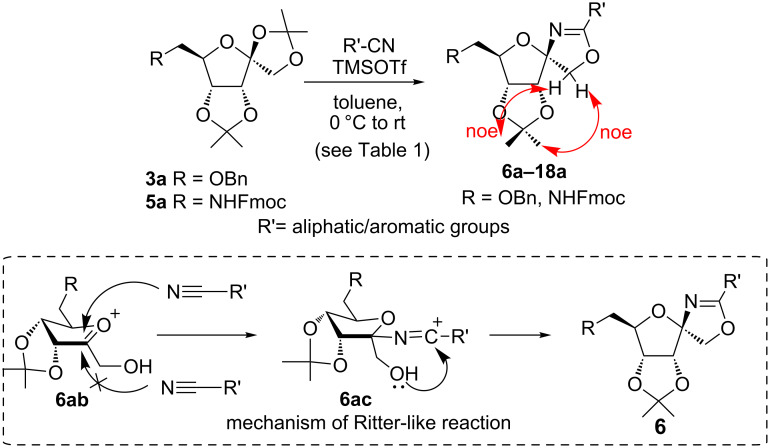
Synthesis of spirooxazolines.

In our first experiment, compound **5a** was reacted with acetonitrile as a nucleophilic participating solvent using TMSOTf at 0 °C, then the reaction was left at room temperature for 1 h. To our delight, the complete consumption of the starting material and the formation of spiro 2-substituted-2-oxazoline **6a** in 69% yield was observed. This Ritter-like reaction is known to proceed via TMSOTf-mediated in situ cleavage of the 1,2-*O*-isopropylidene group, generating an oxacarbenium ion and its nucleophilic addition to the nitrile (Scheme 2, **6ab**). This occurs with simultaneous intramolecular trapping of the C2 hydroxymethyl group on the electrophilic nitrilium carbon (Scheme 2, **6ac**) giving spiro 2-substituted-2-oxazoline **6** (Scheme 2). It is important to note that the 3,4-*O*-isopropylidene group is unaffected under these conditions, indicating the stability of the *cis*-fused 5-membered ring systems. The formation of a spirooxazoline product was confirmed by the ^13^C NMR spectra from the signals at 110 ppm for the anomeric C2 carbon and at 168 ppm for the N=C carbon, along with the presence of the absorption band at 1657 cm^−1^ for C=N and the absence of an OH band in the IR spectra. Under similar reaction conditions, compound **5a** reacted with propionitrile as a solvent and nitrile source to obtain product **7a** in 72% isolated yield (Table 1, entry 2). To optimize the reaction conditions for nitrile reagents, the reaction of **5a** (0.2 g, 0.415 mmol) was performed at different concentrations (2.5, 5, 10, and 15 equiv) of cyclohexane carbonitrile (Table 1, entry 3) in dry toluene using TMSOTf (1 equiv) at 0 °C to rt under N_2_ atmosphere. A complex mixture of products was obtained with 2.5 equiv and 5 equiv of nitrile, along with trace amounts of product. Using 10 equiv of cyclohexane carbonitrile gave moderate yield of product **8a** along with the formation of di-D-fructose dianhydride or spiroketals [49] as the side product. Finally, 15 equiv of nitrile served best for this transformation providing spirooxazoline **8a** in 65% yield. This result suggested that under a lower concentration of nitrile, or in the absence of a nucleophilic nitrogen, the oxacarbenium-ion intermediate of D-fructose underwent spiroketalization, giving spirocyclic disaccharides as reported earlier [49] along with other unidentified products.

**Table 1:** Synthesis of spiro 2-substituted-2-oxazolines **6a–18a**.

Entry	Substrate	Nitrile	Product	Yield^a^ (%)

1	**5a**	CH_3_CN	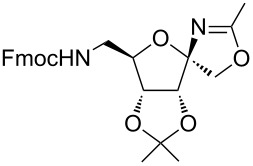 **6a**	69%
2	**5a**	CH_3_CH_2_CN	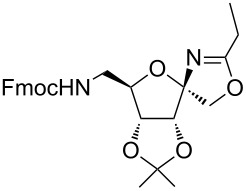 **7a**	72%
3	**5a**	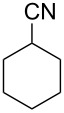	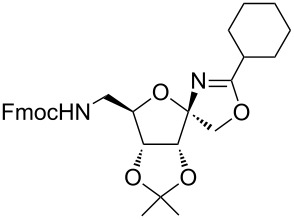 **8a**	65%
4	**5a**	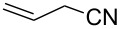	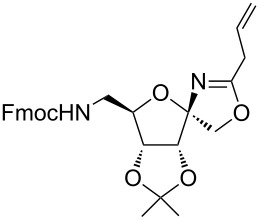 **9a**	53%
5	**5a**	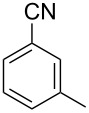	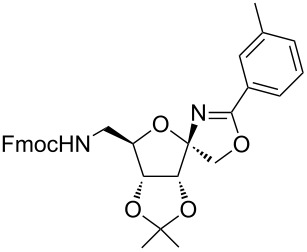 **10a**	61%
6	**3a**	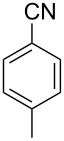	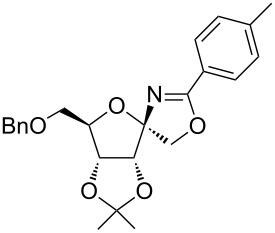 **11a**	52%
7	**3a**	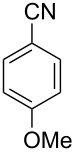	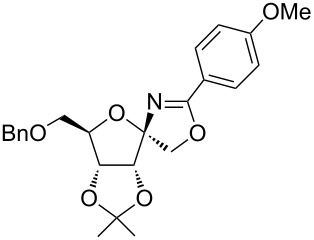 **12a**	72%
8	**5a**^b^	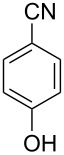	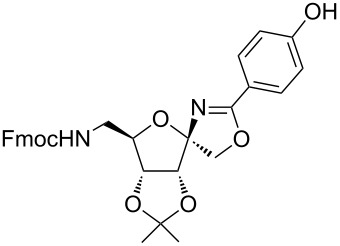 **13a**	69%
9	**3a**	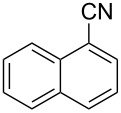	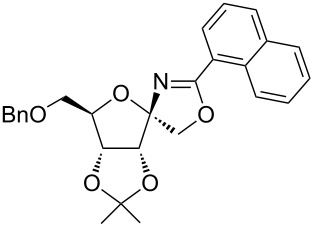 **14a**	50%
10	**3a**	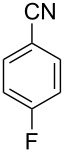	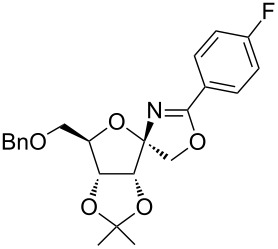 **15a**	66%
11	**5a**	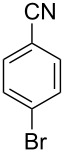	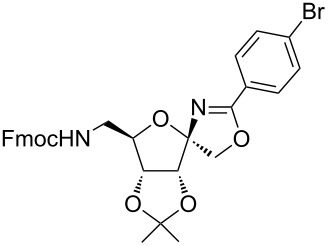 **16a**	44%
12	**3a**	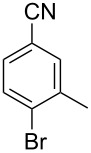	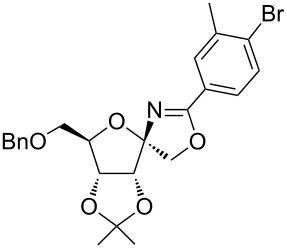 **17a**	45%
13	**5a**	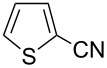	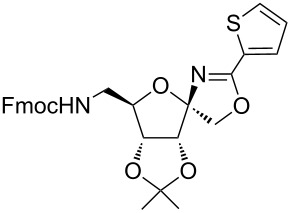 **18a**	45%

^a^Conditions: All reactions were performed with 0.2 g of substrate, 1 equiv TMSOTf, 15 equiv nitrile in toluene (except entries 1 and 2) at 0 °C to rt for 1–1.5 h. ^b^As 4-hydroxybenzonitrile is insoluble in toluene, the reaction was carried out in 10:1 DCM/THF.

**Scheme 3:**
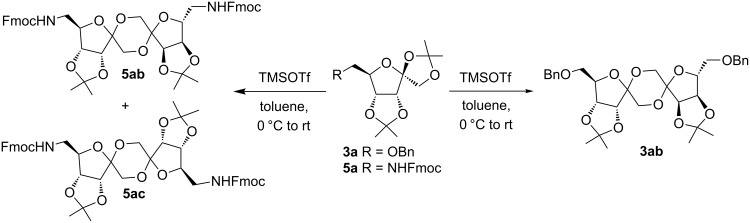
Formation of spiroketals from **3a, 5a**.

With this optimization, we began to explore the scope of the reaction by employing various aliphatic and aromatic nitriles bearing electron-donating and electron-withdrawing substituents. Accordingly, compound **5a** was treated with allyl cyanide (Table 1, entry 4) to get **9a** in 53% yield in a stereoselectively manner. Next, the reaction was carried out with electron-withdrawing groups containing aliphatic nitriles such as bromoacetonitrile, trichloroacetonitrile and malonitrile. However, in all the reactions, no product formation was observed, and instead, the reaction mixture gave spiroketals. Attention then was turned to check the feasibility of the reaction with different substituted, aromatic nitriles. In this respect, electron-rich aromatic nitriles, such as 3-methyl, 4-methyl, 4-methoxybenzonitrile (Table 1, entries 5–7) and 1-naphthonitrile (Table 1, entry 9), reacted with **3a** and **5a** affording products **10a–12a** and **14a**, in 61%, 52%, 72% and 50% yields. In the case of 4-hydroxybenzonitrile (Table 1, entry 8), because of its insolubility in toluene, the reaction was carried out in 10:1 DCM:THF under similar reaction conditions. It is important to note that no protection of the hydroxy group is required and the desired oxazoline product **13a** was obtained as the major product in 69% yield. Of the halogen containing benzonitriles, 4-fluorobenzonitrile (Table 1, entry 10) gave **15a** in 66% yield, whereas 4-bromo and 4-bromo-3-methylbenzonitriles (Table 1, entries 11 and 12) gave the corresponding products **16a** and **17a** in moderate yields. Finally, 2-cyanothiophene (Table 1, entry 13) also reacted with **5a** to give **18a** in 45% yield. Further, 4-nitrobenzonitrile and 2-cyanopyridine with an electron-withdrawing group or ring did not give the expected product due to the reduced nucleophilicity of the nitrile group. In all the low yielding reactions, spiroketals are observed in 15–20% yield. This was evidenced by activating **3a** and **5a** with TMSOTf (1 equiv) in toluene in the absence of a nucleophile. While **3a** gave a symmetric spiroketal **3ab** with a single set of protons in 75% yield (Scheme 3), compound **5a** gave a separable 1:1 mixture of symmetric **5ab** and asymmetric **5ac** spiroketals in 74% yield (NMR and HRMS data provided in Supporting Information File 1).

It is worth noting that contrary to the observed instability of furanoid spirooxazolines by Garcia et al., all psicofuranose-derived ribosyl spiro 2-substituted-2-oxazolines were stable and obtained as a single diastereomer. To assign the relative stereochemistry at the anomeric center, compounds **11a** and **17a** were subjected to 2D NMR analysis. In the NOESY spectra of compounds **11a** and **17a**, the cross peaks between C1–H, C1–H’ and CH_3_ groups of acetonide confirmed the α-orientation of the anomeric CH_2_–O group and β-orientation of the *N*-substituent (see Supporting Information File 1, pages S47 and S48). This stereochemical outcome is due to the presence of the 3,4-acetonide group in α-orientation, thereby directing the nucleophile to attack from the β-orientation, leading to a high stereoselectivity. Finally, due to the widespread applicability of chiral oxazolines, the synthesis of fructose-derived spirooxazolines was examined on a multigram scale. For example, the reaction of **5a** (1.2 g, 2.49 mmol) with cyclohexane carbonitrile (15 equiv) in 12 mL of toluene activated by TMSOTf (1 equiv) at 0 °C to rt for 1.5 h provided **8a** in 61% yield.

## Conclusion

In conclusion, TMSOTf-activated protected D-psicofuranose derivatives in the presence of an excess of electron-rich nitriles permitted the synthesis of spiro 2-substituted-2-oxazoline ribosides as a single diastereomer in good yield. In the absence of the nucleophile, the oxacarbenium-ion intermediate of protected D-psicofuranose derivatives underwent spiroketalization to give di-D-fructose dianhydrides. Given the established, pharmacological applications of riboside derivatives, these stable, conformationally constrained riboside spirooxazolines open up the possible application in the development of new drug molecules. In addition, these highly functionalized glycooxazolines can be used to make covalently cross-linked polymers for application in nanomedicine and material science.

## Supporting Information

File 1 Detailed experimental procedures, compound characterization, copies of ^1^H, ^13^C NMR spectra of all new compounds, NOESY spectra of **11a**, **17a** and HRMS spectra of all new compounds.

## References

[1] Bergeron R J, Xin M G, Weimar W R, Smith R E, Wiegand J (2001). J Med Chem.

[2] Davidson B S (1993). Chem Rev.

[3] Tsuda M, Yamakawa M, Oka S, Tanaka Y, Hoshino Y, Mikami Y, Sato A, Fujiwara H, Ohizumi Y, Kobayashi J (2005). J Nat Prod.

[4] Li Q, Woods K W, Claiborne A, Gwaltney S L, Barr K J, Liu G, Gehrke L, Credo R B, Hui Y H, Lee J (2002). Bioorg Med Chem Lett.

[5] Onishi H R, Pelak B A, Gerckens L S, Silver L L, Kahan F M, Chen M-H, Patchett A A, Galloway S M, Hyland S A, Anderson M S (1996). Science.

[6] Clark D, Travis D A (2001). Bioorg Med Chem.

[7] Bedekar A V, Andersson P G (1996). Tetrahedron Lett.

[8] Goyard D, Telligmann S M, Goux-Henry C, Boysen M M K, Framery E, Gueyrard D, Vidal S (2010). Tetrahedron Lett.

[9] Irmak M, Groschner A, Boysen M M K (2007). Chem Commun.

[10] Minuth T, Irmak M, Groschner A, Lehnert T, Boysen M M K (2009). Eur J Org Chem.

[11] Adams N, Schubert U S (2007). Adv Drug Delivery Rev.

[12] Hoogenboom R (2009). Angew Chem, Int Ed.

[13] Hartlieb M, Kempe K, Schubert U S (2015). J Mater Chem B.

[14] Greene T W, Wuts P G M (1991). Protective Groups in Organic Synthesis.

[15] Lusskin R M, Ritter J J (1950). J Am Chem Soc.

[16] Allen J V, Williams J M J (1994). Tetrahedron: Asymmetry.

[17] Rajaram S, Sigman M S (2002). Org Lett.

[18] Ohshima T, Iwasakia T, Mashima K (2006). Chem Commun.

[19] Minakata S, Nishimura M, Takahashi T, Oderaotoshi Y, Komatsu M (2001). Tetrahedron Lett.

[20] Rising T W D F, Heidecke C D, Moir J W B, Ling Z, Fairbanks A J (2008). Chem – Eur J.

[21] Noguchi M, Tanaka T, Gyakushi H, Kobayashi A, Shoda S-i (2009). J Org Chem.

[22] Zeng Y, Wang J, Li B, Hauser S, Li H, Wang L-X (2006). Chem – Eur J.

[23] Paulsen H (1982). Angew Chem.

[24] Banoub J, Boullanger P, Lafont D (1992). Chem Rev.

[25] Bongat A F G, Demchenko A V (2007). Carbohydr Res.

[26] Schweizer F, Lohse A, Otter A, Hindsgaul O (2001). Synlett.

[27] Damkaci F, DeShong P (2003). J Am Chem Soc.

[28] De Castro M, Marzabadi C H (2004). Tetrahedron Lett.

[29] De Castro M, Marzabadi C H (2005). J Carbohydr Chem.

[30] Ritter J J, Minieri P P (1948). J Am Chem Soc.

[31] Kuimen L I, Cota D J (1969). Org React.

[32] Gordon D M, Danishefsky S J (1991). J Org Chem.

[33] Klein H, Mietchen R, Reinke H, Michalik M (1999). J Prakt Chem.

[34] Crich D, Patel M (2006). Carbohydr Res.

[35] Jiménez Blanco J L, Sylla B, Mellet C O, García Fernández J M (2007). J Org Chem.

[36] Pavia A A, Ung-Chhun S N, Durand J L (1981). J Org Chem.

[37] Jiménez Blanco J L, Rubio E M, Mellet C O, García Fernández J M (2004). Synlett.

[38] Chao C-S, Lin C-Y, Mulani S, Hung W-C, Mong K-k T (2011). Chem – Eur J.

[39] Awan S I, Werz D B (2012). Bioorg Med Chem.

[40] Koester D C, Holkenbrink A, Werz D B (2010). Synthesis.

[41] Kurhade S E, Ravula S, Siddaiah V, Bhuniya D, Reddy D S (2011). Tetrahedron Lett.

[42] Dell’Isola A, McLachlan M M W, Neuman B W, Al-Mullah H M N, Binks A W D, Elvidge W, Shankland K, Cobb A J A (2014). Chem – Eur J.

[43] Gasch C, Pradera M A, Salameh B A B, Molina J L, Fuentes J (2001). Tetrahedron: Asymmetry.

[44] Vangala M, Dhokale S A, Gawade R L, Pattuparambil R R, Puranik V G, Dhavale D D (2013). Org Biomol Chem.

[45] Bhuma N, Vangala M, Nair R J, Sabharwal S G, Dhavale D D (2015). Carbohydr Res.

[46] Nakajima M, Itoi K, Takamatsu Y, Kinoshita T, Okazaki T, Kawakubo K, Shindo M, Honma T, Tohjigamori M, Haneishi T (1991). J Antibiot.

[47] Perali R S, Mandava S, Bandi R (2011). Tetrahedron.

[48] Joseph C C, Regeling H, Zwanenburg B, Chittenden G J F (2002). Carbohydr Res.

[49] Benito J M, Gómez-García M, Mellet C O, García Fernández J M, Defaye J (2001). Org Lett.

