# Supplementary material for: Synthesis of D-fructose-derived spirocyclic 2-substituted-2-oxazoline ribosides
Source: Beilstein J Org Chem. 2015 Nov 24;11:2289–96. doi: 10.3762/bjoc.11.249 (PMC4685833; doi:10.3762/bjoc.11.249)
Supplement: File 1 — Detailed experimental procedures, compound characterization, copies of 1H, 13C NMR spectra of all new compounds, NOESY spectra of 11a, 17a and HRMS spectra of all new compounds. [file Beilstein_J_Org_Chem-11-2289-s001.pdf]

Supporting Information

for

Synthesis of D-fructose derived spirocyclic 2-substituted-2-oxazoline ribosides

Madhuri Vangala* and Ganesh P Shinde

Address: Department of Chemistry, Indian Institute of Science Education and Research, Pune 411 008, India

Email: Madhuri Vangala* - madhuri@iiserpune.ac.in

* Corresponding author

Detailed experimental procedures, compound characterization, copies of ^{1}H, ^{13}C NMR spectra of all new compounds, NOESY spectra of 11a, 17a and HRMS spectra of all new compounds

Section	Content	Pages
1	Experimental section and data	S2–S6
2	^{1}H, ^{13}C NMR spectra of compounds **2a–17a**, **3ab**, **5ab**, **5ac**	S7–S46
3	NOESY spectra of **11a**, **17a**	S47, S48
4	HRMS spectra of compounds **5a–17a, 3ab**, **5ab**, **5ac**	S49–S57

EXPERIMENTAL SECTION

General Methods. All reagents were purchased from commercial sources and were used without further purification unless otherwise stated. Reactions were carried out with distilled and dried solvents using oven-dried glassware. ^{1}H NMR and ^{13}C NMR were recorded in $CDCl_3$ using TMS as internal standard on 400 and 100 MHz spectrometer respectively. Coupling constants (J) are reported in Hertz (Hz). In ^{1}H NMR spectra signal multiplicity is abbreviated as s = singlet, d = doublet, dd = doublet of doublet, dt = doublet of triplet, td = triplet of doublet, qd, quartet of doublet, t = triplet, q = quartet, m = multiplet, br = broad. IR spectra were recorded on a FT-IR spectrometer, and only major peaks were reported in cm^{-1}. Optical rotations were measured on a polarimeter using sodium light (D line 589 nm). High resolution mass spectroscopy (HRMS) were obtained by the ESI (TOF) ionization sources.

1,2:3,4-Di-O-isopropylidene-β-D-psicofuranose (***2a***). Thick liquid: $[\alpha]_D^{25}$ = -80.3 (c 1.02, $CHCl_3$); ^{1}H NMR (400 MHz, $CDCl_3$) δ 4.90 (dd, J = 5.9, 0.8 Hz, 1H), 4.63 (d, J = 5.9 Hz, 1H), 4.32 (d, J = 9.7 Hz, 1H), 4.28 (t, J = 2.8 Hz, 1H), 4.06 (d, J = 5.9 Hz, 1H), 3.74 (dt, J = 12.6, 2.8 Hz, 1H), 3.67 – 3.59 (m, 1H), 3.20 (dd, J = 10.5, 3.0 Hz, 1H), 1.49 (s, 3H), 1.43 (s, 3H), 1.39 (s, 3H), 1.31 (s, 3H); ^{13}C NMR (100 MHz, $CDCl_3$) δ 113.5, 112.4, 111.8, 86.9, 85.9, 81.7, 70.0, 64.0, 26.6, 26.4, 26.2; 24.9; IR ($CHCl_3$) v 3495, 2988, 1376, 1205, 1015, 852 cm^{-1}; HRMS (TOF): $[M.+ Na]^+$ calcd for $C_{12}H_{20}NaO_6$ 283.1158, found 283.1159.

1,2:3,4-di-O-isopropylidene-6-O-benzyl-β-D-psicofuranose (***3a***). To a stirring solution of **2a** (2 g, 7.6 mmol) in DMF at 0 °C, NaH (0.4 g, 10.0 mmol) was added under N_2 atmosphere and stirred for 10 min. To this, benzyl bromide (1 mL, 8.4 mmol) was added drop-wise. The resulting mixture was slowly allowed to warm to room temperature and stirred overnight. After completion of starting material saturated aq. $NaHCO_3$ is added and extracted with EtOAc. The combined organic layers were washed with water, brine, dried over Na_2SO_4 filtered and concentrated *in vacuo*. Purification of the crude product by column chromatography using petroleum ether/EtOAC gave the title compound **3a**. Thick liquid; R_f = 0.5 (petroleum ether/EtOAC, 85:15); Yield: (2.4 g, 92%); $[\alpha]_D^{25}$ = -65.6 (c 0.67, $CHCl_3$); ^{1}H NMR (400 MHz, $CDCl_3$) δ 7.40 – 7.25 (m, 5H,), 4.76 (d, J = 5.9 Hz, 1H), 4.61 (d, J = 5.7 Hz, 1H), 4.57 (d, J = 4.9 Hz, 2H), 4.34-4.27 (m, 2H), 4.06 (d, J = 9.6 Hz, 1H), 3.62 – 3.50 (m, 2H), 1.45 (s, 3H), 1.44 (s, 3H), 1.38 (s, 3H), 1.33 (s, 3H); ^{13}C NMR (100 MHz, $CDCl_3$) δ 138.1, 128.4, 127.6, 113.5, 112.6, 111.5, 85.1, 83.9, 82.5, 73.3, 70.9, 69.8, 26.6, 26.5, 26.4, 25.2; IR ($CHCl_3$) v 3495, 2988, 1376, 1205, 1015, 852 cm^{-1}; HRMS (TOF): $[M.+ Na]^+$ calcd for $C_{19}H_{26}NaO_6$ 373.1627, found 373.1631.

6-Azido-6-deoxy-1,2;3,4-di-O-isopropylidene-β-D-psicofuranose (***4a***). Thick liquid: $[\alpha]_D^{25}$ = -53.0 (c 1.06, $CHCl_3$); ^{1}H NMR (400 MHz, $CDCl_3$) δ 4.63 (dd, J = 5.9, 1.0 Hz, 1H), 4.59 (d, J = 5.8 Hz, 1H), 4.30 (d, J = 9.7 Hz, 1H), 4.21 – 4.15 (m, 1H), 4.04 (d, J = 9.9 Hz, 1H), 3.51 (dd, J = 12.6, 7.6 Hz, 1H), 3.28 (dd, J = 12.6, 6.8 Hz, 1H), 1.46 (s, 3H), 1.43 (s, 3H), 1.37 (s, 3H), 1.30 (s, 3H); ^{13}C NMR (100 MHz, $CDCl_3$) δ 113.9, 113.0, 112.0, 85.3, 84.2, 82.5, 69.7, 53.3, 26.5, 26.5, 26.3, 25.2; IR ($CHCl_3$) v 2989, 2099, 1376, 1205, 1065, 1020, 853 cm^{-1}; HRMS (TOF): $[M + Na]^+$ calcd for $C_{12}H_{19}N_3NaO_5$ 308.1222, found 308.1219.

6-*deoxy-6-((9H-fluoren-9-yl)methylcarbamate)-1,2;3,4-di-O-isopropylidene-β-D-psicofuranose* (***5a)***. To a solution of compound **4a** (3.5 g, 12.2 mmol) in EtOAc (15 mL) in a RB flask was added 10% Pd–C 0.5 g. The mixture was hydrogenated using H_2 balloon at room temperature for 3 h. After completion of reaction the slurry was filtered through celite and concentrated *in vacuo* to give amine quantitatively. This crude amine was dissolved in THF and 10% $NaHCO_3$ soln (7.5 mL) added. The resulting solution was cooled to 0 °C and Fmoc-OSu 1.1 eq (4.5 g, 13.5 mmol) with respect to amine was added slowly as a solution in THF. The resulting mixture was allowed to warm to room temperature overnight. Water was then added and extracted with EtOAc (100 mL X 3). The combined organic layers were washed with water, brine and dried over Na_2SO_4 and concentrated to dryness. The

dry residue was chromatographed on a column of silica gel using petroleum ether/EtOAC to get **5a**. White solid: R_f = 0.5 (petroleum ether/EtOAC, 75:25); Yield: (5.0 g, 85%); $[\alpha]_D^{25}$ = -53.0 (*c* 0.7, $CHCl_3$); mp- 102-104 ºC; ^{1}H NMR (400 MHz, $CDCl_3$) δ 7.77 (d, *J* = 7.5 Hz, 2H), 7.62 – 7.57 (m, 2H), 7.40 (t, *J* = 7.4 Hz, 2H), 7.31 (t, *J* = 7.4 Hz, 2H), 5.54 (t, *J* = 5.4 Hz, 1H), 4.66 (d, *J* = 5.8 Hz, 1H), 4.59 (d, *J* = 5.7 Hz, 1H), 4.48 – 4.37 (m, 2H), 4.34 (d, *J* = 9.8 Hz, 1H), 4.25 (dt, *J* = 13.9, 6.2 Hz, 2H), 4.06 (d, *J* = 9.9 Hz, 1H), 3.42 (t, *J* = 5.7 Hz, 2H), 1.49 (s, 3H), 1.44 (s, 3H), 1.41 (s, 3H), 1.31 (s, 3H); ^{13}C NMR (100 MHz, $CDCl_3$) δ 156.6, 144.1, 144.0, 141.5, 141.4, 127.8, 127.1, 125.1, 120.1, 113.7, 112.8, 111.8, 85.7, 84.6, 82.3, 70.0, 66.8, 47.3, 43.6, 26.5, 26.5, 26.4, 25.2; IR ($CHCl_3$) ν 3356, 2988, 1718, 1519, 1243, 1064, 855, 733 cm^{-1}; HRMS (TOF): $[M + Na]^+$ calcd for $C_{27}H_{31}NNaO_7$ 504.1998, found 504.1998.

General procedure for the synthesis of spirooxazolines **6a** & **7a**.

To a solution of psicofuranose derivative **5a** (0.2 g, 0.415 mmol) and acetonitrile (**a**) (2 mL) or propionitrile (**b**) (2 mL) at 0 °C was added TMSOTf (0.074 mL, 0.415 mmol) under N_2 atmosphere. After addition, the reaction mixture was stirred at room temperature for 1-1.5 h. After complete consumption of the starting material, triethylamine was added to quench the reaction. The mixture was concentrated *in vacuo*. Water is then added and extracted with EtOAc (20 mL X 2). The combined organic layers were washed with water, brine and dried over Na_2SO_4 and concentrated to dryness. The dry residue was chromatographed on a column of silica gel using petroleum ether (bp 60−70 °C) and EtOAc to afford compounds **6a** & **7a**.

General procedure for the synthesis of spiro oxazolines **8a-18a**.

To a solution of psicofuranose derivative **3a** (0.2 g, 0.57 mmol) or **5a** (0.2 g, 0.415 mmol) and nitrile (**c-m**) (15 equiv) in toluene (2 mL) at 0 °C was added TMSOTf (1 equiv) under N_2 atmosphere. After addition, the reaction mixture was stirred at room temperature for 1-1.5 h. After complete consumption of the starting material, triethylamine was added to quench the reaction and followed the same workup and purification procedure as above mentioned to afford compounds **8a-18a**.

Compound (***6a***). White foam: R_f = 0.3 (petroleum ether/EtOAc, 70:30); Yield: (133 mg, 69%); $[\alpha]_D^{25}$ = -44.8 (*c* 0.24, $CHCl_3$); ^{1}H NMR (400 MHz, $CDCl_3$) δ 7.77 (d, *J* = 7.5 Hz, 2H), 7.68-7.60 (m, 2H), 7.40 (t, *J* = 7.4 Hz, 2H), 7.35 – 7.26 (m, 2H), 7.06 (d, *J* = 5.5 Hz, 1H), 4.77 (d, *J* = 5.8 Hz, 1H), 4.58 (d, *J* = 10.8 Hz, 1H), 4.52 (d, *J* = 5.9 Hz, 1H), 4.40 (d, *J* = 7.3 Hz, 2H), 4.35 (br, 1H), 4.24 (t, *J* = 7.0 Hz, 1H), 4.18 (d, *J* = 10.8 Hz, 1H), 3.63 (ddd, *J* = 13.2, 8.2, 4.7 Hz, 1H), 3.34 (dt, *J* = 14.3, 3.1 Hz, 1H), 2.05 (s, 3H), 1.46 (s, 3H), 1.32 (s, 3H); ^{13}C NMR (100 MHz, $CDCl_3$) δ 168.2, 156.9, 144.2, 144.1, 141.4, 127.7, 127.0, 125.3, 125.2, 120.0, 112.7, 110.1, 86.2, 84.6, 83.3, 72.5, 66.6, 47.4, 43.8, 26.5, 25.1, 14.5; IR ($CHCl_3$) ν 3303, 2939, 1709, 1657, 1517, 1214, 1075, 741 cm^{-1}; HRMS (TOF): $[M + H]^+$ calcd for $C_{26}H_{29}N_2O_6$ 465.2026, found 465.2029.

Compound (***7a***).Thick liquid: R_f = 0.4 (petroleum ether/EtOAc, 80:20); Yield: (143 mg, 72%); $[\alpha]_D^{25}$ = -73.1 (*c* 0.2, $CHCl_3$); ^{1}H NMR (400 MHz, $CDCl_3$) δ 7.76 (d, *J* = 7.5 Hz, 2H), 7.63 (t, *J* = 7.1 Hz, 2H), 7.43 – 7.34 (m, 2H), 7.32 – 7.26 (m, 2H), 4.77 (d, *J* = 5.9 Hz, 1H), 4.57 (d, *J* = 10.8 Hz, 1H), 4.51 (d, *J* = 5.9 Hz, 1H), 4.41 (d, *J* = 8.0 Hz, 2H), 4.37 (t, *J* = 3.2 Hz, 1H), 4.23 (t, *J* = 7.0 Hz, 1H), 4.18 (d, *J* = 10.8 Hz, 1H), 3.73 – 3.63 (m, 1H), 3.31 (dt, *J* = 14.3, 2.6 Hz, 1H), 2.33 (qd, *J* = 7.5, 3.1 Hz, 2H), 1.46 (s, 3H), 1.33 (s, 3H), 1.18 (t, *J* = 7.5 Hz, 3H); ^{13}C NMR (100 MHz, $CDCl_3$) δ 172.4, 157.0, 144.3, 144.0, 141.4, 127.7, 127.0, 125.3, 125.2, 120.0, 112.6, 109.7, 86.2, 84.7, 83.2, 72.4, 66.6, 47.4, 43.8, 26.7, 25.1, 21.8, 10.0. IR ($CHCl_3$) ν 3246, 2931, 1720, 1656, 1536, 1250, 1085, 749 cm^{-1}; HRMS (TOF): $[M + H]^+$ calcd for $C_{27}H_{31}N_2O_6$ 479.2182, found 479.2184.

Compound (***8a***).White foam: R_f = 0.4 (petroleum ether/EtOAc, 80:20); Yield: (143 mg, 65%); $[\alpha]_D^{25}$ = -67.2 (*c* 0.88, $CHCl_3$); ^{1}H NMR (400 MHz, $CDCl_3$) δ 7.77 (d, *J* = 7.5 Hz, 2H), 7.65 (t, 8.3 Hz, 2H), 7.47 (d, *J* = 7.4 Hz, 1H), 7.40 (t, *J* = 7.4 Hz, 2H), 7.34 – 7.27 (m, 2H), 4.78 (d, *J* = 5.9 Hz, 1H), 4.55 (d, *J* = 10.8 Hz, 1H), 4.52 (d, *J* = 5.9 Hz, 1H), 4.41 (d, *J* = 7.6 Hz, 2H), 4.37 (br s, 1H), 4.23 (t, *J* = 7.1 Hz, 1H), 4.17 (d, *J* = 10.8 Hz, 1H), 3.71 (ddd, *J* = 13.4, 8.9, 4.2 Hz, 1H), 3.31 (dt, *J* = 14.3, 2.5 Hz,

1H), 2.37-2.27 (m, 1H), 2.03-1.91 (m, 2H), 1.79 – 1.69 (m, 2H), 1.67-1.57 (m, 1H), 1.54-1.48 (m, 1H), 1.47 (s, 3H), 1.45 – 1.37 (m, 1H), 1.34 (s, 3H), 1.31 – 1.18 (m, 3H); ^{13}C NMR (100 MHz, $CDCl_3$) δ 174.4, 157.0, 144.4, 144.1, 141.4 127.7, 127.1, 127.0, 125.2, 120.0, 112.6, 109.7, 86.4, 84.6, 83.3, 72.1, 66.8, 47.5, 43.9, 37.5, 29.8, 29.6, 26.6, 25.8, 25.6, 25.5, 25.2; IR ($CHCl_3$) ν 3247, 2932, 1716, 1648, 1534, 1249, 1057, 747 cm^{-1}; HRMS (TOF): [M + H]$^+$ calcd for $C_{31}H_{37}N_2O_6$ 533.2652, found 533.2652.

Compound (***9a***). Sticky liquid: R_f = 0.4 (petroleum ether/EtOAc, 70:30); Yield: (107 mg, 53%); $[\alpha]_D^{25}$ = -52.3 (*c* 0.3, $CHCl_3$); ^{1}H NMR (400 MHz, $CDCl_3$); 7.77 (d, *J* = 7.5 Hz, 2H), 7.69 – 7.59 (m, 2H), 7.40 (t, *J* = 7.5 Hz, 2H), 7.30 (t, *J* = 7.4 Hz, 2H), 7.19 (d, *J* = 6.1 Hz, 1H), 6.86 – 6.73 (m, 1H), 5.98 (dd, *J* = 15.8, 1.7 Hz, 1H), 5.24 – 5.10 (m, 1H), 4.79 (d, *J* = 5.8 Hz, 1H), 4.61 (d, *J* = 10.7 Hz, 1H), 4.54 (d, *J* = 5.8 Hz, 1H), 4.46 – 4.34 (m, 3H), 4.29 – 4.18 (m, 2H), 3.71 – 3.59 (m, 1H), 3.40 – 3.29 (m, 1H), 1.89 (dd, *J* = 6.9, 1.6 Hz, 2H), 1.47 (s, 3H), 1.33 (s, 3H); ^{13}C NMR (100 MHz, $CDCl_3$) δ 165.4, 157.0, 144.3, 144.2, 142.3, 141.5, 130.5, 127.8, 127.1, 125.3, 120.1, 118.7, 112.7, 110.0, 86.4, 84.8, 83.4, 72.0, 66.7, 47.5, 44.0, 33.0, 26.6, 25.2; IR ($CHCl_3$) ν 3253, 2931, 1713, 1674, 1606, 1532, 1247, 1053, 746 cm^{-1}; HRMS (TOF): [M + H]$^+$ calcd for $C_{28}H_{31}N_2O_6$ 491.2182, found 491.2189.

Compound (***10a***). Light brown foam: R_f = 0.3 (petroleum ether/EtOAc, 80:20); Yield: (137 mg, 61%); $[\alpha]_D^{25}$ = -85.8 (*c* 1.0, $CHCl_3$); ^{1}H NMR (400 MHz, $CDCl_3$) δ 7.81 (d, *J* = 7.2 Hz, 2H), 7.75 (d, *J* = 7.6 Hz, 2H), 7.66 (d, *J* = 7.5 Hz, 2H), 7.45 (d, *J* = 6.5 Hz, 1H), 7.41-7.33 (m, 2H), 7.32 – 7.18 (m, 4H), 4.89 (d, *J* = 5.8 Hz, 1H), 4.79 (d, *J* = 10.8 Hz, 1H), 4.67 (d, *J* = 5.9 Hz, 1H), 4.51 – 4.36 (m, 4H), 4.30 (t, *J* = 7.3 Hz, 1H), 3.76 (ddd, *J* = 13.0, 8.4, 4.3 Hz, 1H), 3.42 (dt, *J* = 14.2, 2.8 Hz, 1H), 2.26 (s, 3H), 1.51 (s, 3H), 1.37 (s, 3H); ^{13}C NMR (100 MHz, $CDCl_3$) δ 166.6, 157.0, 144.2, 141.4, 138.4, 133.2, 129.4, 128.6, 127.8, 127.2, 127.1, 126.8, 126.0, 125.3, 120.1, 112.8, 110.2, 86.5, 85.0, 83.5, 72.6, 67.0, 47.6, 44.0, 26.7, 25.3, 21.4; IR ($CHCl_3$) ν 3245, 2940, 1713, 1641, 1246, 1078, 745 cm^{-1}; HRMS (TOF): [M + H]$^+$ calcd for $C_{32}H_{33}N_2O_6$ 541.2339, found 541.2339.

Compound (***11a***). Thick liquid: R_f = 0.5 (petroleum ether/EtOAc, 80:20); Yield: (121 mg, 52%); $[\alpha]_D^{25}$ = -96.8 (*c* 0.46, $CHCl_3$); ^{1}H NMR (400 MHz, $CDCl_3$) δ 7.72 (d, *J* = 8.2 Hz, 2H), 7.30 – 7.17 (m, 5H), 7.13 (d, *J* = 7.9 Hz, 2H), 4.81 (dd, *J* = 5.9, 1.4 Hz, 1H), 4.59 (d, *J* = 10.8 Hz, 1H), 4.57 (d, *J* = 6.2 Hz, 1H), 4.54 (d, *J* = 4.6 Hz, 2H), 4.32 – 4.26 (m, 2H), 3.74 – 3.63 (m, 2H), 2.32 (s, 3H), 1.42 (s, 3H), 1.27 (s, 3H); ^{13}C NMR (100 MHz, $CDCl_3$) 166.2, 142.5, 138.4, 129.2, 128.8, 128.5, 127.9, 127.7, 124.8, 112.8, 110.4, 85.9, 84.3, 83.7, 73.5, 72.2, 71.3, 26.7, 25.4, 21.7; IR ($CHCl_3$) ν 2933, 1641, 1351, 1079, 736 cm^{-1}; HRMS (TOF): [M + H]$^+$ calcd for $C_{24}H_{28}NO_5$ 410.1967, found 410.1976.

Compound (***12a***). Thick liquid: R_f = 0.4 (petroleum ether/EtOAc, 85:15); Yield: (174 mg, 72%); $[\alpha]_D^{25}$ = -74.9 (*c* 0.48, $CHCl_3$); ^{1}H NMR (400 MHz, $CDCl_3$) δ 7.85 (d, *J* = 9.0 Hz, 2H), 7.40 – 7.24 (m, 5H), 6.90 (d, *J* = 9 Hz, 2H), 4.88 (dd, *J* = 5.9, 1.4 Hz, 1H), 4.66 (d, *J* = 7.9 Hz, 1H), 4.64 (d, *J* = 3.1 Hz, 1H), 4.61 (d, *J* = 4.5 Hz, 2H), 4.41 – 4.32 (m, 2H), 3.85 (s, 3H), 3.81-3.70 (m, 2H), 1.50 (s, 3H), 1.35 (s, 3H); ^{13}C NMR (100 MHz, $CDCl_3$) δ 166.0, 162.7, 138.4, 130.6, 128.5, 127.9, 127.7, 120.0, 113.8, 112.8, 110.4, 85.9, 84.2, 83.7, 73.5, 72.2, 71.3, 55.5, 26.7, 25.4. IR ($CHCl_3$) ν 2936, 1638, 1609, 1512, 1254, 1078, 741 cm^{-1}; HRMS (TOF): [M + H]$^+$ calcd for $C_{24}H_{28}NO_6$ 426.1917, found 426.1925.

Compound (***13a***). White foam: R_f = 0.4 (petroleum ether/EtOAc, 60:40); Yield: (155 mg, 69%); $[\alpha]_D^{25}$ = -69.1 (*c* 0.3, $CHCl_3$); ^{1}H NMR (400 MHz, $CDCl_3$) δ 7.88 (d, *J* = 8.7 Hz, 2H), 7.75 (d, *J* = 7.6 Hz, 2H), 7.65 (d, *J* = 7.5 Hz, 2H), 7.58 (d, *J* = 6.6 Hz, 1H), 7.38 (td, *J* = 7.5, 2.1 Hz, 2H), 7.25 (td, *J* = 7.4, 2.9 Hz, 2H), 6.79 (d, *J* = 8.7 Hz, 2H), 4.86 (d, *J* = 5.8 Hz, 1H), 4.77 (d, *J* = 10.8 Hz, 1H), 4.65 (d, *J* = 5.9 Hz, 1H), 4.52 – 4.36 (m, 4H), 4.26 (t, *J* = 7.1 Hz, 1H), 3.78 – 3.68 (m, 1H), 3.40 (dt, *J* = 14.1, 2.8 Hz, 1H), 1.52 (s, 3H), 1.37 (s, 3H); ^{13}C NMR (100 MHz, $CDCl_3$) δ 166.5, 159.9, 157.3, 144.2, 144.1, 141.4, 131.0, 127.8, 127.2, 125.3, 120.1, 119.0, 115.7, 112.8, 110.1, 86.4, 84.8, 83.4, 72.6, 67.1, 47.5, 44.0, 26.7, 25.3; IR ($CHCl_3$) ν 3237, 2937, 1695, 1635, 1515, 1262, 1080, 746 cm^{-1}; HRMS (TOF): [M + H]$^+$ calcd for $C_{31}H_{31}N_2O_7$ 543.2131, found 543.2131.

Compound (***14a***). Thick liquid: R_f = 0.5 (petroleum ether/EtOAc, 85:15); Yield: (127 mg, 50%); $[\alpha]_D^{25}$ = -100.8 (*c* 0.87, $CHCl_3$); ^{1}H NMR (400 MHz, $CDCl_3$) *δ* 9.14-9.05 (m, 1H), 8.13 (dd, *J* = 7.3, 1.2 Hz, 1H), 8.01 (d, *J* = 8.2 Hz, 1H), 7.95 – 7.86 (m, 1H), 7.60 – 7.48 (m, 3H), 7.44 – 7.25 (m, 5H), 4.98 (dd, *J* = 5.9, 1.2 Hz, 1H), 4.79 (d, *J* = 5.9 Hz, 1H), 4.76 (d, *J* = 10.7 Hz, 1H), 4.72 (d, *J* = 12.1 Hz, 1H), 4.64 (d, *J* = 12.1 Hz, 1H), 4.48 (t, *J* = 6.8 Hz, 1H), 4.45 (d, *J* = 10.8 Hz, 1H), 3.83 (m, 2H), 1.57 (s, 3H), 1.41 (s, 3H); ^{13}C NMR (100 MHz, $CDCl_3$) *δ* 165.9, 138.3, 133.9, 132.7, 131.3, 129.7, 128.7, 128.5, 127.9, 127.8, 127.4, 126.4, 126.3, 124.8, 124.0, 112.8, 111.0, 86.0, 84.3, 83.8, 73.6, 71.1, 26.7, 25.4; IR ($CHCl_3$) *v* 2936, 1638, 1511, 1208, 1077, 777 cm^{-1}; HRMS (TOF): $[M + H]^+$ calcd for $C_{27}H_{28}NO_5$ 446.1967, found 446.1964.

Compound (***15a***). Thick liquid: R_f = 0.4 (petroleum ether/EtOAc, 80:20); Yield: (155 mg, 66%); $[\alpha]_D^{25}$ = -82.9 (*c* 1.21, $CHCl_3$); ^{1}H NMR (400 MHz, $CDCl_3$) *δ* 7.97 – 7.90 (m, 2H), 7.42 – 7.26 (m, 5H), 7.11 (t, *J* = 8.7 Hz, 2H), 4.92 (dd, *J* = 5.9, 1.4 Hz, 1H), 4.72 (d, *J* = 10.7 Hz, 1H), 4.67 (d, *J* = 6.0 Hz, 1H), 4.64 (d, *J* = 2.7 Hz, 2H), 4.44 – 4.38 (m, 2H), 3.82 – 3.73 (m, 2H), 1.53 (s, 3H), 1.38 (s, 3H); ^{13}C NMR (100 MHz, $CDCl_3$, C−F coupling not assigned) *δ* 166.4, 165.1, 163.9, 138.3, 131.1, 131.0, 129.1, 128.4, 127.8, 127.7, 115.7, 115.5, 112.8, 110.4, 85.8, 84.2, 83.6, 73.5, 72.4, 71.1, 26.6, 25.3; IR ($CHCl_3$) *v* 2936, 1643, 1509, 1215, 1073, 739 cm^{-1}; HRMS (TOF): $[M + H]^+$ calcd for $C_{23}H_{25}FNO_5$ 414.1717, found 414.1719.

Compound (***16a***). White foam: R_f = 0.3 (petroleum ether/EtOAc, 80:20); Yield: (110 mg, 44%); $[\alpha]_D^{25}$ = -77.2 (*c* 0.19, $CHCl_3$); ^{1}H NMR (400 MHz, $CDCl_3$) *δ* 7.80 (d, *J* = 8.5 Hz, 2H), 7.74 (dd, *J* = 7.5, 3.8 Hz, 2H), 7.63 (dd, *J* = 7.4, 2.1 Hz, 2H), 7.47 (d, *J* = 8.5 Hz, 2H), 7.38 (dd, *J* = 13.1, 7.3 Hz, 2H), 7.26 – 7.18 (m, 2H), 4.83 (dd, *J* = 5.9, 1.2 Hz, 1H), 4.78 (d, *J* = 10.9 Hz, 1H), 4.59 (d, *J* = 5.8 Hz, 1H), 4.54 – 4.42 (m, 2H), 4.39 (d, *J* = 10.9 Hz, 2H), 4.26 (t, *J* = 6.9 Hz, 1H), 3.71 (ddd, *J* = 13.0, 8.4, 4.4 Hz, 1H), 3.37 (dt, *J* = 14.2, 2.9 Hz, 1H), 1.49 (s, 3H), 1.36 (s, 3H); ^{13}C NMR (100 MHz, $CDCl_3$) *δ* 165.7, 157.0, 144.3, 144.1, 141.5, 141.5, 132.1, 130.3, 127.8, 127.3, 127.2, 125.9, 125.2, 120.1, 112.9, 110.2, 86.3, 85.1, 83.4, 72.8, 66.8, 47.6, 43.9, 26.7, 25.3; IR ($CHCl_3$) *v* 3261, 2933, 1717, 1642, 1246, 1081, 743 cm^{-1}; HRMS (TOF): $[M + H]^+$ calcd for $C_{31}H_{30}BrN_2O_6$ 605.1287, found 605.1284.

Compound (***17a***). Thick liquid: R_f = 0.4 (petroleum ether/EtOAc, 85:15); Yield: (125 mg, 45%); $[\alpha]_D^{25}$ = -74.5 (*c* 0.39, $CHCl_3$); ^{1}H NMR (400 MHz, $CDCl_3$) *δ* 7.79 (s, 1H), 7.59 (d, *J* = 1.2 Hz, 2H), 7.42 – 7.26 (m, 5H), 4.91 (dd, *J* = 5.9, 1.4 Hz, 1H), 4.70 (d, *J* = 10.7 Hz, 1H), 4.66 (d, *J* = 6.0 Hz, 1H), 4.64 (d, *J* = 2.6 Hz, 2H), 4.44 – 4.36 (m, 2H), 3.82 – 3.71 (m, 2H), 2.45 (s, 3H), 1.53 (s, 3H), 1.37 (s, 3H); ^{13}C NMR (100 MHz, $CDCl_3$) *δ* 165.5, 138.3, 132.6, 130.8, 129.3, 128.5, 127.8, 127.7, 127.5, 126.7, 112.9, 110.4, 85.8, 84.3, 83.6, 73.5, 72.4, 71.2, 26.7, 25.4, 23.0; IR ($CHCl_3$) *v* 2929, 1642, 1462, 1081, 739 cm^{-1}; HRMS (TOF): $[M + H]^+$ calcd for $C_{24}H_{27}BrNO_5$ 488.1073, found 488.1079.

Compound (***18a***). White solid: R_f = 0.4 (petroleum ether/EtOAc, 70:30); Yield: (99 mg, 45%); $[\alpha]_D^{25}$ = -84.4 (*c* 0.56, $CHCl_3$); mp- 137-139 ºC; ^{1}H NMR (400 MHz, $CDCl_3$) *δ* 7.75 (d, *J* = 7.6 Hz, 2H), 7.71-7.65 (m, 3H), 7.49 (dd, *J* = 4.9, 1.0 Hz, 1H), 7.38 (td, *J* = 7.4, 2.6 Hz, 2H), 7.25 (t, *J* = 7.5 Hz, 2H), 7.20 (br, 1H), 7.06 (t, *J* = 3.9 Hz, 1H), 4.84 (d, *J* = 5.9 Hz, 1H), 4.77 (d, *J* = 10.7 Hz, 1H), 4.65 (d, *J* = 5.9 Hz, 1H), 4.47 – 4.36 (m, 4H), 4.28 (t, *J* = 7.2 Hz, 1H), 3.78 – 3.69 (m, 1H), 3.37 (dt, *J* = 14.3, 2.8 Hz, 1H), 1.49 (s, 3H), 1.34 (s, 3H); ^{13}C NMR (100 MHz, $CDCl_3$) *δ* 162.1, 157.0, 144.3, 144.1, 141.4, 132.0, 131.5, 129.4, 128.1, 127.7, 127.1, 125.4, 125.3, 120.0, 112.8, 110.2, 86.3, 84.9, 83.3, 73.0, 66.8, 47.5, 43.9, 26.6, 25.1; IR ($CHCl_3$) *v* 3267, 2929, 1714, 1638, 1521, 1245, 1070, 748 cm^{-1}; HRMS (TOF): $[M + H]^+$ calcd for $C_{29}H_{29}N_2O_6S$ 533.1746, found 533.1747.

Compound (***3ab***). To a solution of **3a** (0.2 g, 0.57 mmol) in toluene at 0 °C, was added TMSOTf (0.103 mL, 0.57 mmol). After addition ice bath was removed and reaction stirred at room temperature. After 45 min complete consumption of starting material was observed and reaction

quenched by adding Et_3N. Toluene removed on rota evaporator. Water was added and extracted into EtOAc. The combined organic layers were washed with water, brine and dried over Na_2SO_4 and concentrated to dryness. The dry residue was chromatographed on a column of silica gel using petroleum ether/EtOAC to get **3ab**. Thick liquid: R_f = 0.4 (petroleum ether/EtOAC, 85:15); Yield: (125 mg, 75%); $[\alpha]_D^{25}$ = -74.2 (*c* 0.73, $CHCl_3$); ^1H NMR (400 MHz, $CDCl_3$) δ 7.38 – 7.27 (m, 5H X 2), 4.73 (dd, *J* = 6.0, 1.1 Hz, 1H X 2), 4.59 (d, *J* = 6.0 Hz, 1H X 2), 4.54 (d, *J* = 2.1 Hz, 2H X 2), 4.34 – 4.27 (m, 1H X 2), 3.95 (d, *J* = 12.5 Hz, 1H X 2), 3.78 (d, *J* = 12.5 Hz, 1H X 2), 3.59 – 3.46 (m, 2H X 2), 1.46 (s, 3H X 2), 1.32 (s, 3H X 2); ^{13}C NMR (100 MHz, $CDCl_3$) δ 138.1, 128.5, 127.9, 113.1, 106.4, 84.2, 83.7, 83.0, 73.4, 71.0, 60.1, 26.5, 25.2; IR ($CHCl_3$) ν 2925, 1375, 1212, 1093 cm^{-1}; HRMS (TOF): $[M + Na]^+$ calcd for $C_{32}H_{40}NaO_{10}$ 607.2519, found 607.2515.

Compound (***5ab***). This compound was prepared following the above procedure for **3ab** using **5a** (0.2 g, 0.57 mmol) of the starting material to obtain **5ab**, **5ac** as 1:1 in 74% yield. White solid: R_f = 0.3 (petroleum ether/EtOAC, 60:40); Yield: (70 mg, 40%); $[\alpha]_D^{25}$ = -48.8 (*c* 0.44, $CHCl_3$); mp- 101-103 ºC; ^1H NMR (400 MHz, $CDCl_3$) δ 7.77 (d, *J* = 7.5 Hz, 2H X 2), 7.60 (d, *J* = 7.4 Hz, 2H X 2), 7.40 (t, *J* = 7.5 Hz, 2H X 2), 7.32 (td, *J* = 7.4, 0.9 Hz, 2H X 2), 5.36 (t, *J* = 5.5 Hz, 1H X 2), 4.67 (dd, *J* 1= 8.2, 5.8 Hz, 2H X 2), 4.50 – 4.35 (m, 2H X 2), 4.30 (t, *J* = 5.1 Hz, 1H X 2), 4.22 (t, *J* = 6.9 Hz, 1H X 2), 4.07 (d, *J* = 12.4 Hz, 1H X 2), 3.88 (d, *J* = 12.3 Hz, 1H X 2), 3.53 – 3.33 (m, 2H X 2), 1.49 (s, 3H X 2), 1.35 (s, 3H X 2); ^{13}C NMR (100 MHz, $CDCl_3$) δ 156.6, 144.1, 144.0, 141.5, 127.8, 127.2, 125.2, 120.1, 113.4, 106.0, 85.4, 83.9, 82.6, 66.9, 61.3, 47.4, 44.1, 26.6, 25.1; IR ($CHCl_3$) ν 3345, 2940, 1708, 1519, 1245, 1074, 739 cm^{-1}; HRMS (TOF): $[M + H]^+$ calcd for $C_{48}H_{51}N_2O_{12}$ 847.3442, found 847.3442.

Compound (***5ac***). Thick liquid: R_f = 0.25 (petroleum ether/EtOAC, 60:40); Yield: (60 mg, 34%); $[\alpha]_D^{25}$ = -12.8 (*c* 0.83, $CHCl_3$); ^1H NMR (400 MHz, $CDCl_3$) δ 7.76 (d, *J* = 7.5 Hz, 4H), 7.64 – 7.55 (m, 4H), 7.40 (t, *J* = 7.5 Hz, 4H), 7.35 – 7.28 (m, 4H), 5.35 (br t, 1H), 5.03 (t, *J* = 5.9 Hz, 1H), 4.70 (d, *J* = 5.9 Hz, 1H), 4.59 (d, *J* = 5.8 Hz, 1H), 4.53 – 4.32 (m, 6H), 4.28 – 4.07 (m, 6H), 3.81 (d, *J* = 12.2 Hz, 1H), 3.58 – 3.44 (m, 3H), 3.43 – 3.31 (m, 2H), 1.58 (s, 3H), 1.47 (s, 3H), 1.34 (s, 3H), 1.30 (s, 3H); ^{13}C NMR (100 MHz, $CDCl_3$) δ 156.6, 144.0, 141.4, 127.8, 127.2, 125.2, 125.1, 120.1, 117.1, 113.2, 104.1, 99.3, 85.9, 85.7, 82.5, 82.0, 81.0, 67.0, 66.8, 63.9, 62.8, 47.3, 44.1, 42.5, 26.4, 26.3, 26.1, 25.0; IR ($CHCl_3$) ν 3017, 1712, 1517, 1214, 1086, 741 cm^{-1}; HRMS (TOF): $[M + H]^+$ calcd for $C_{48}H_{51}N_2O_{12}$ 847.3442, found 847.3448.

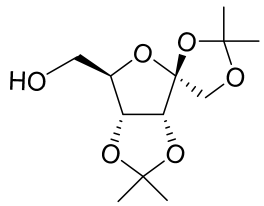

2a

$CDCl_3$, 400 MHz

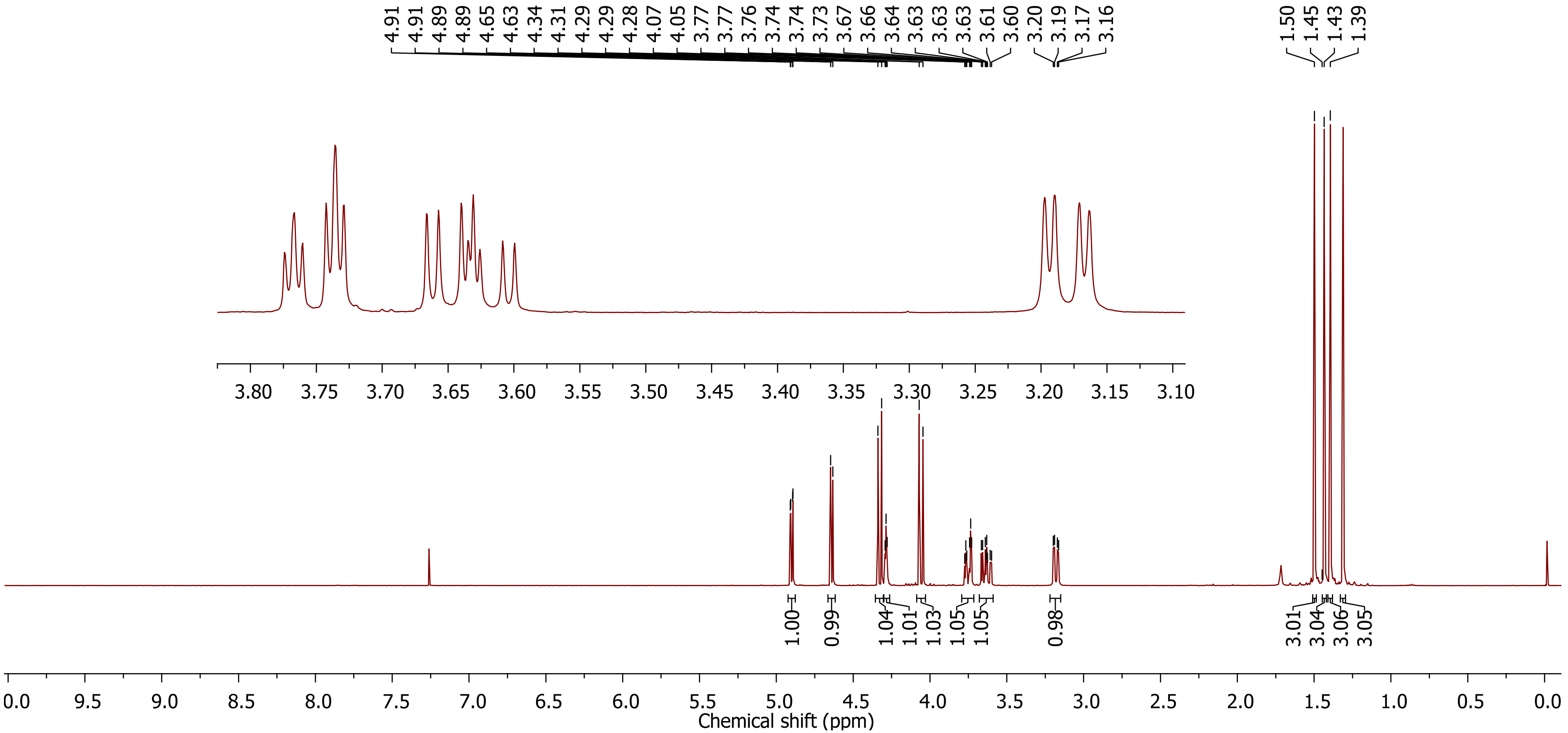

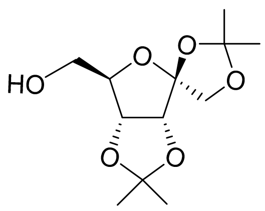

2a
$CDCl_3$, 100 MHz

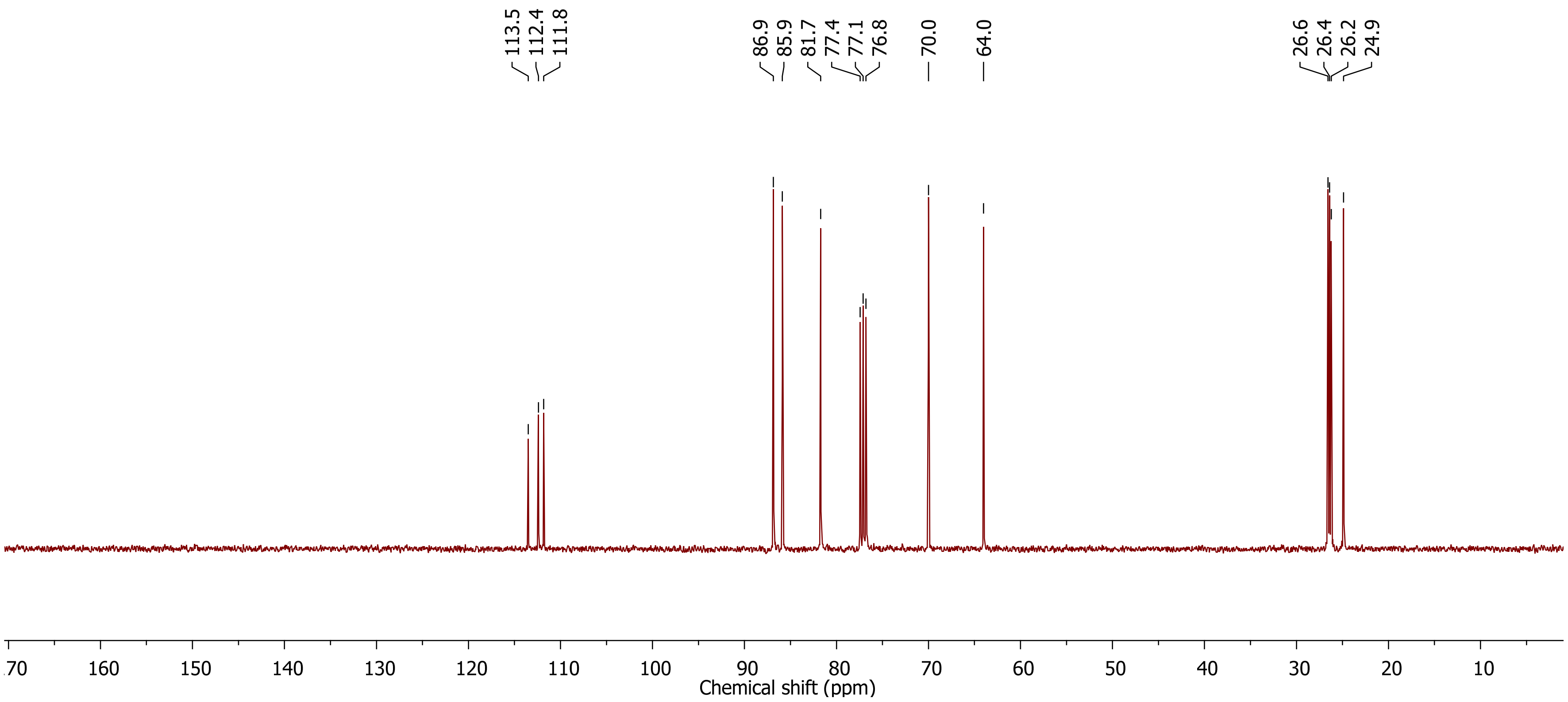

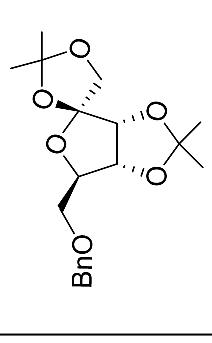

3a

$CDCl_3$, 400 MHz

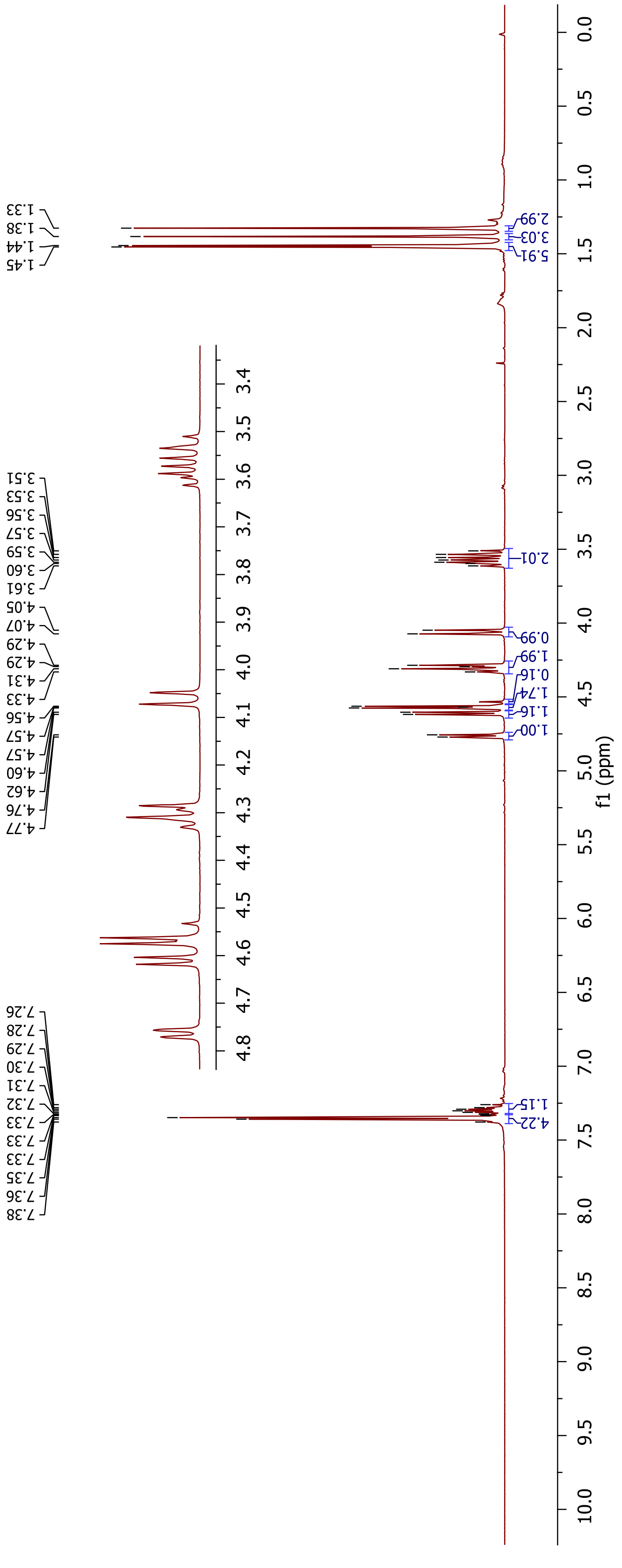

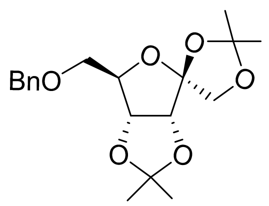

3a

$CDCl_3$, 100 MHz

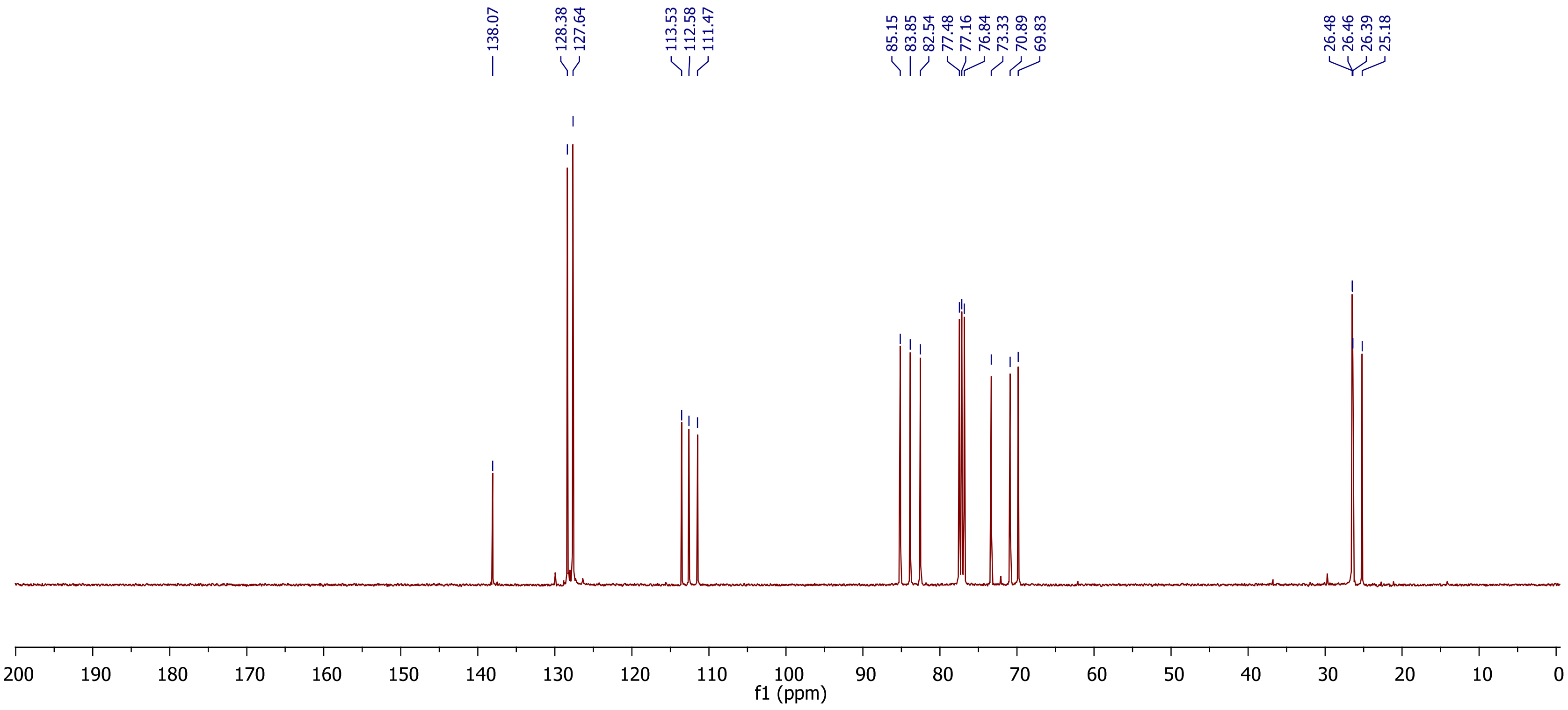

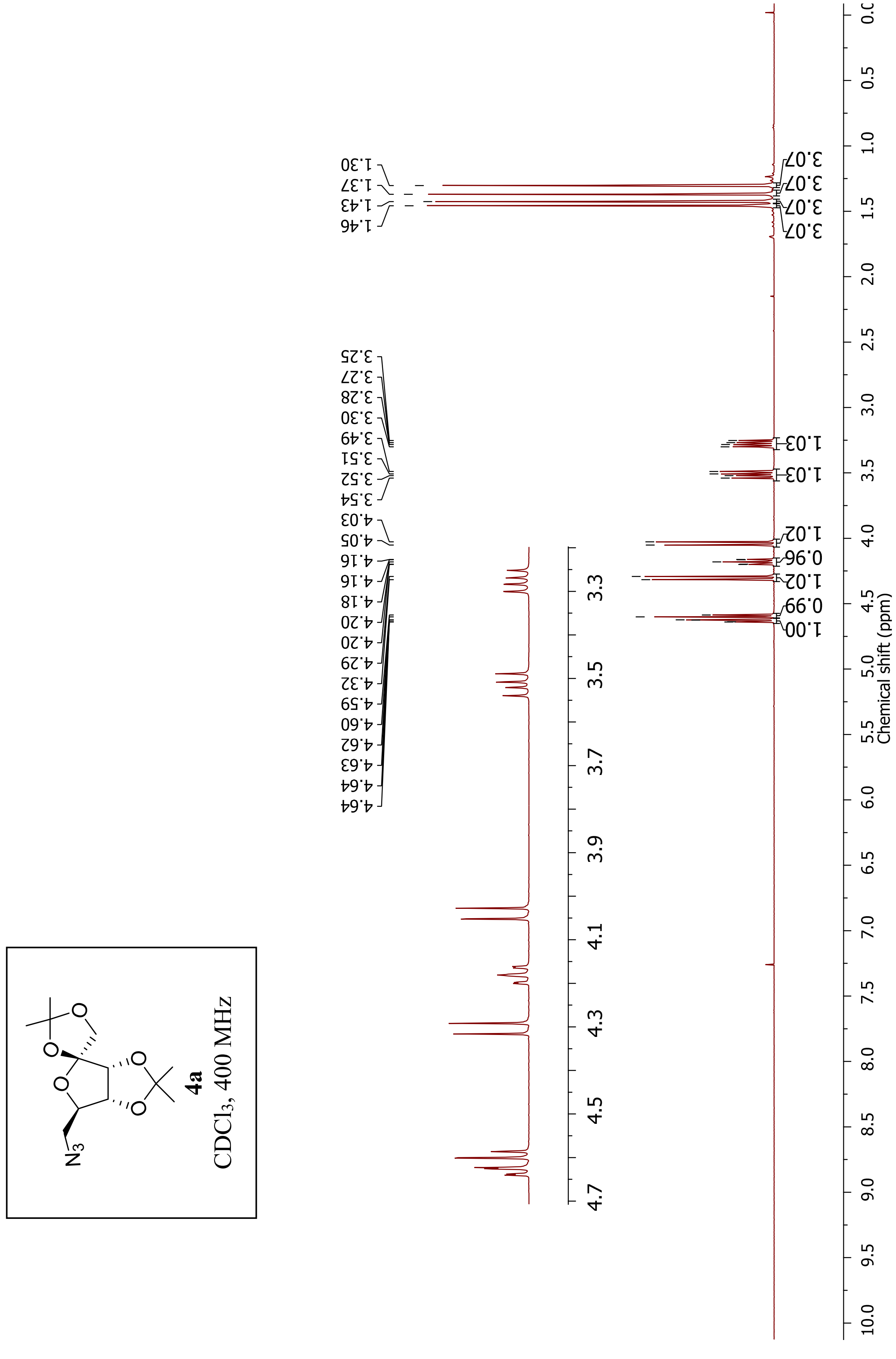

4a
CDCl3, 400 MHz
1.30
1.37
1.43
1.46
3.25
3.27
3.28
3.30
3.49
3.51
3.52
3.54
4.03
4.05
4.16
4.16
4.18
4.20
4.20
4.29
4.32
4.59
4.60
4.62
4.63
4.64
4.64
3.07
3.07
3.07
3.07
1.03
1.03
1.02
0.96
1.02
0.99
1.00
Chemical shift (ppm)

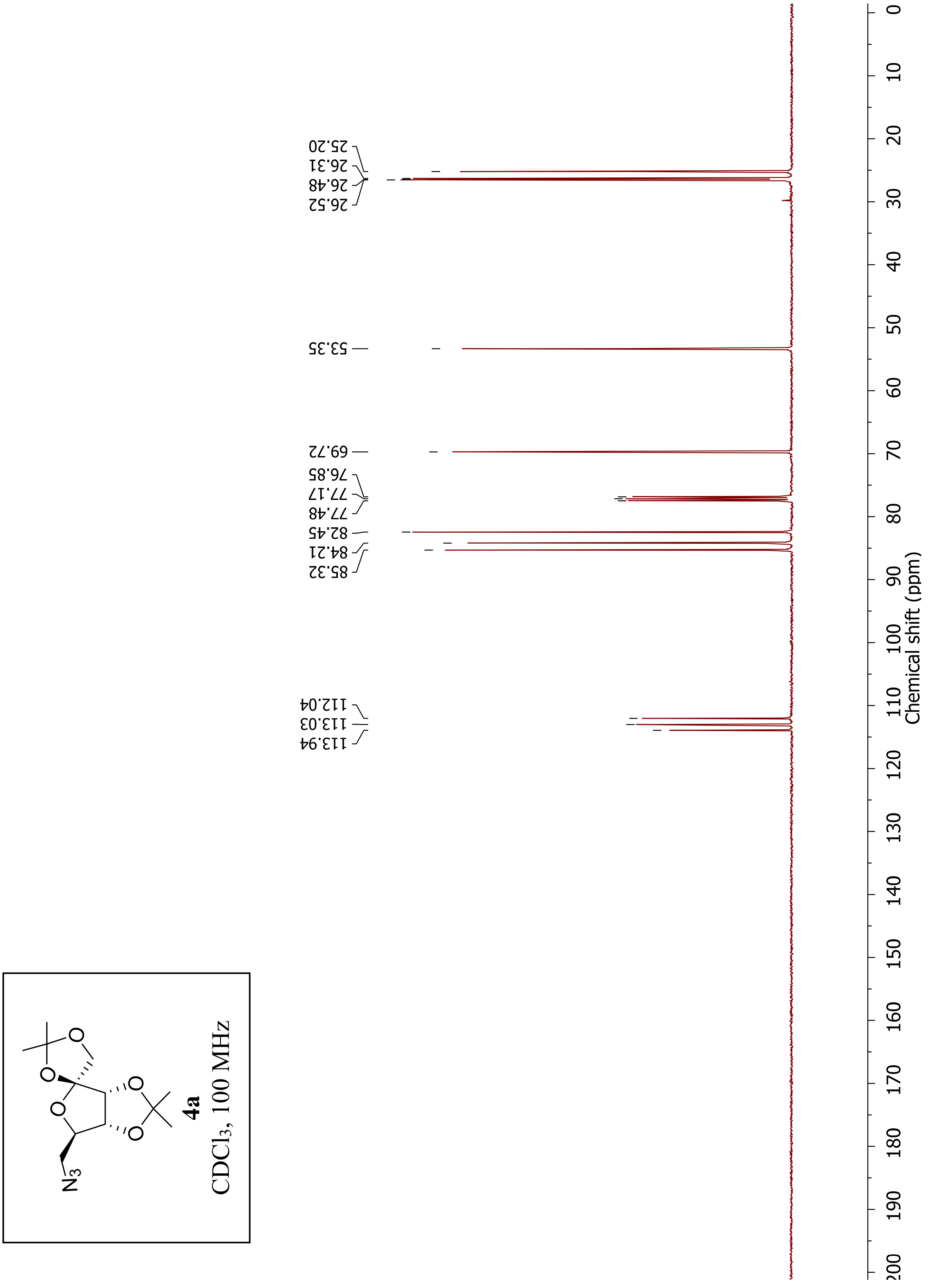
25.20
26.31
26.48
26.52
53.35
69.72
76.85
77.17
77.48
82.45
84.21
85.32
112.04
113.03
113.94
Chemical shift (ppm)
N3
O
4a
CDCl3, 100 MHz

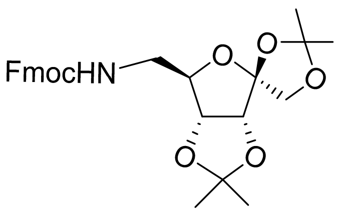

5a

$CDCl_3$, 400 MHz

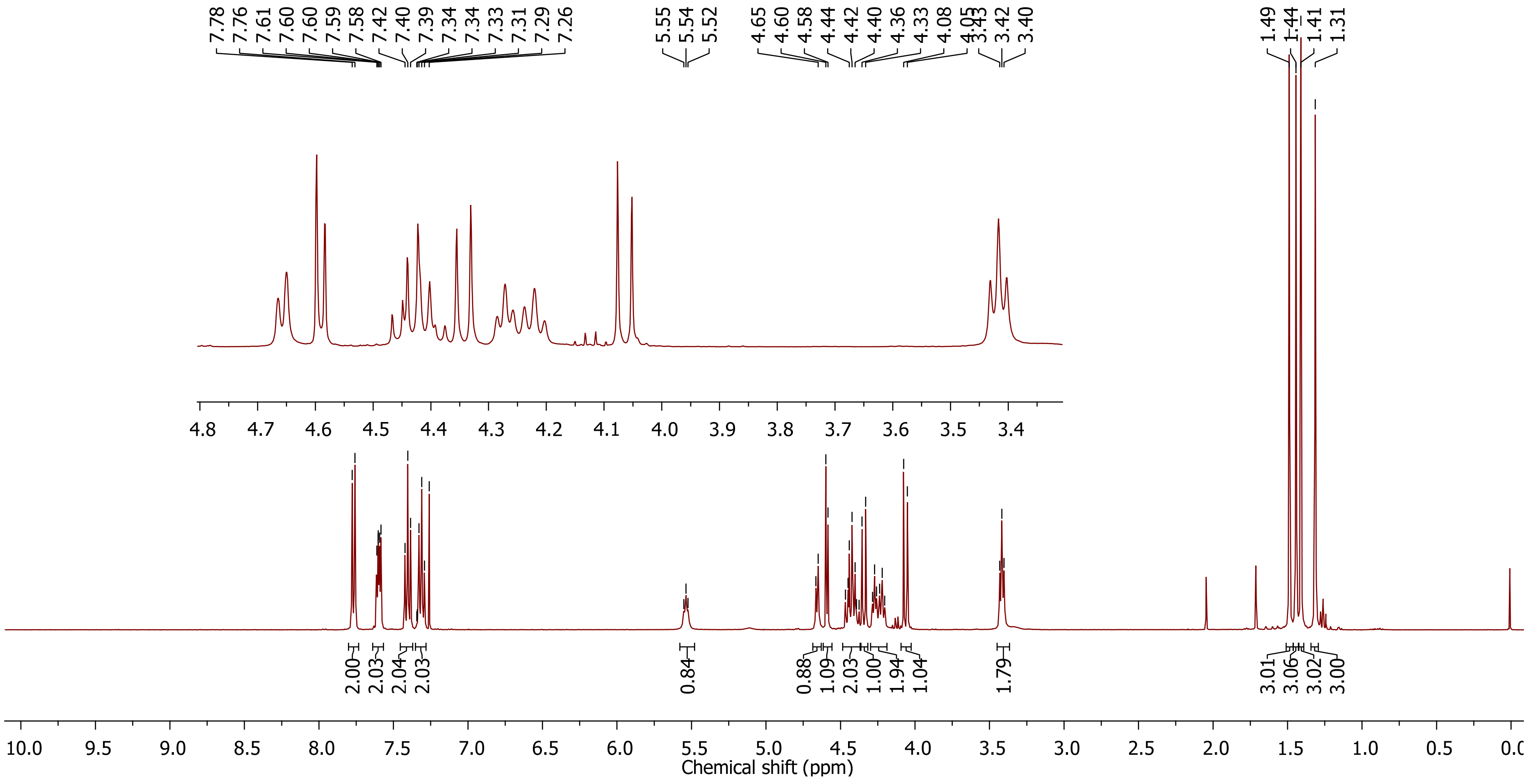

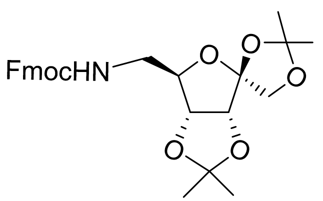

5a
$CDCl_3$, 100 MHz

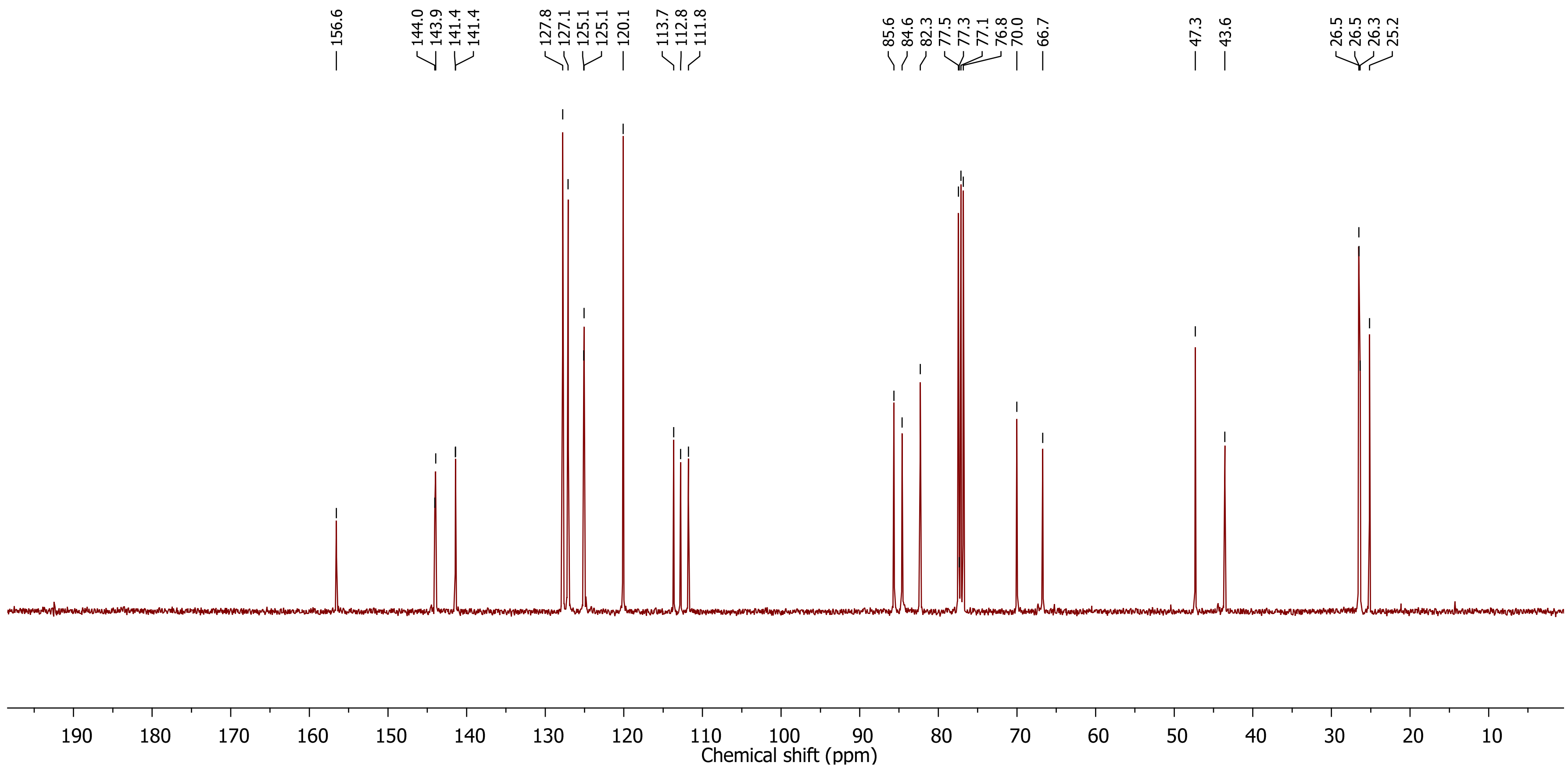

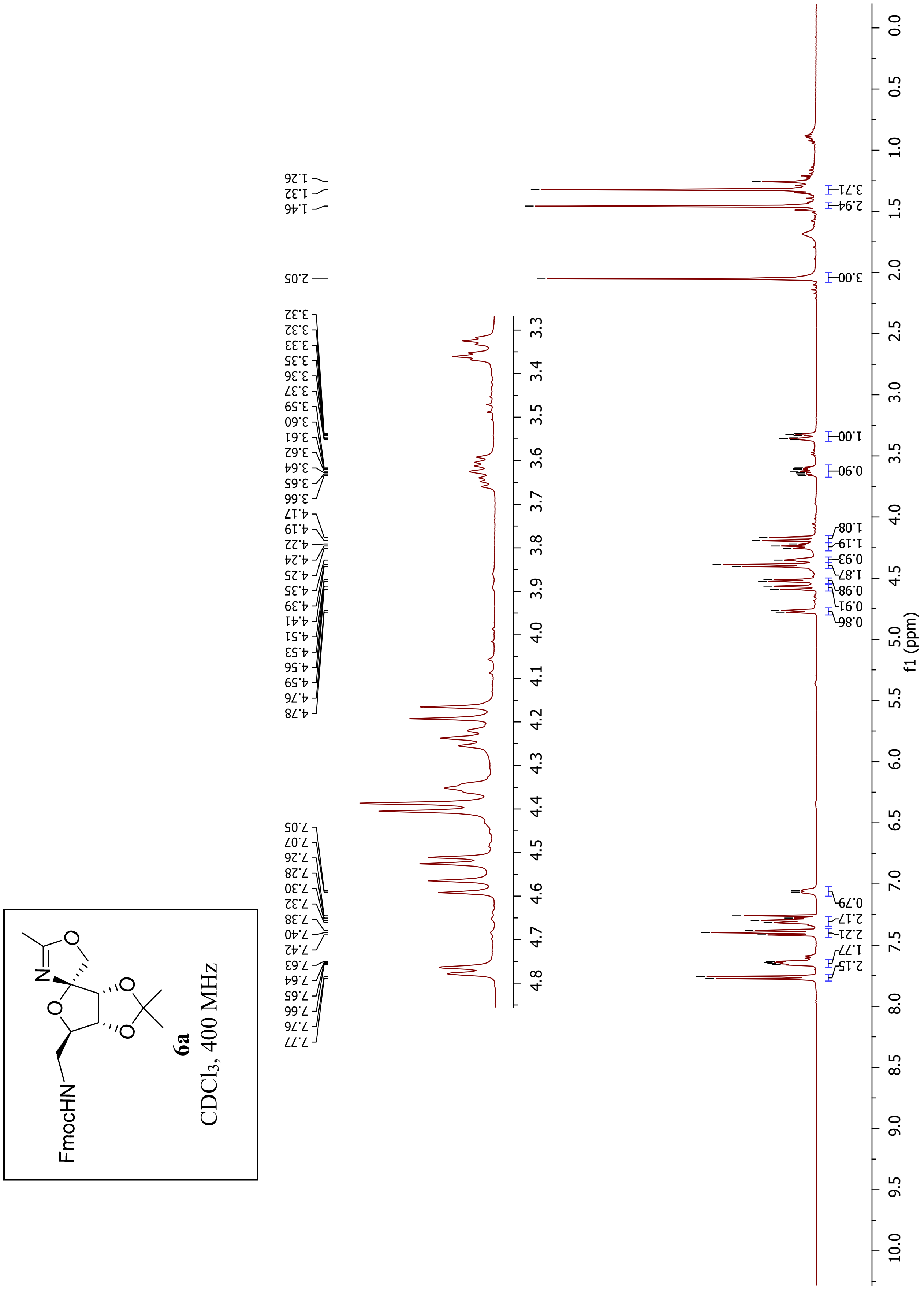
FmocHN
6a
CDCl3, 400 MHz
f1 (ppm)

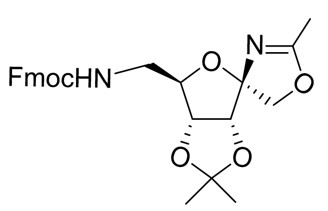

6a

$CDCl_3$, 100 MHz

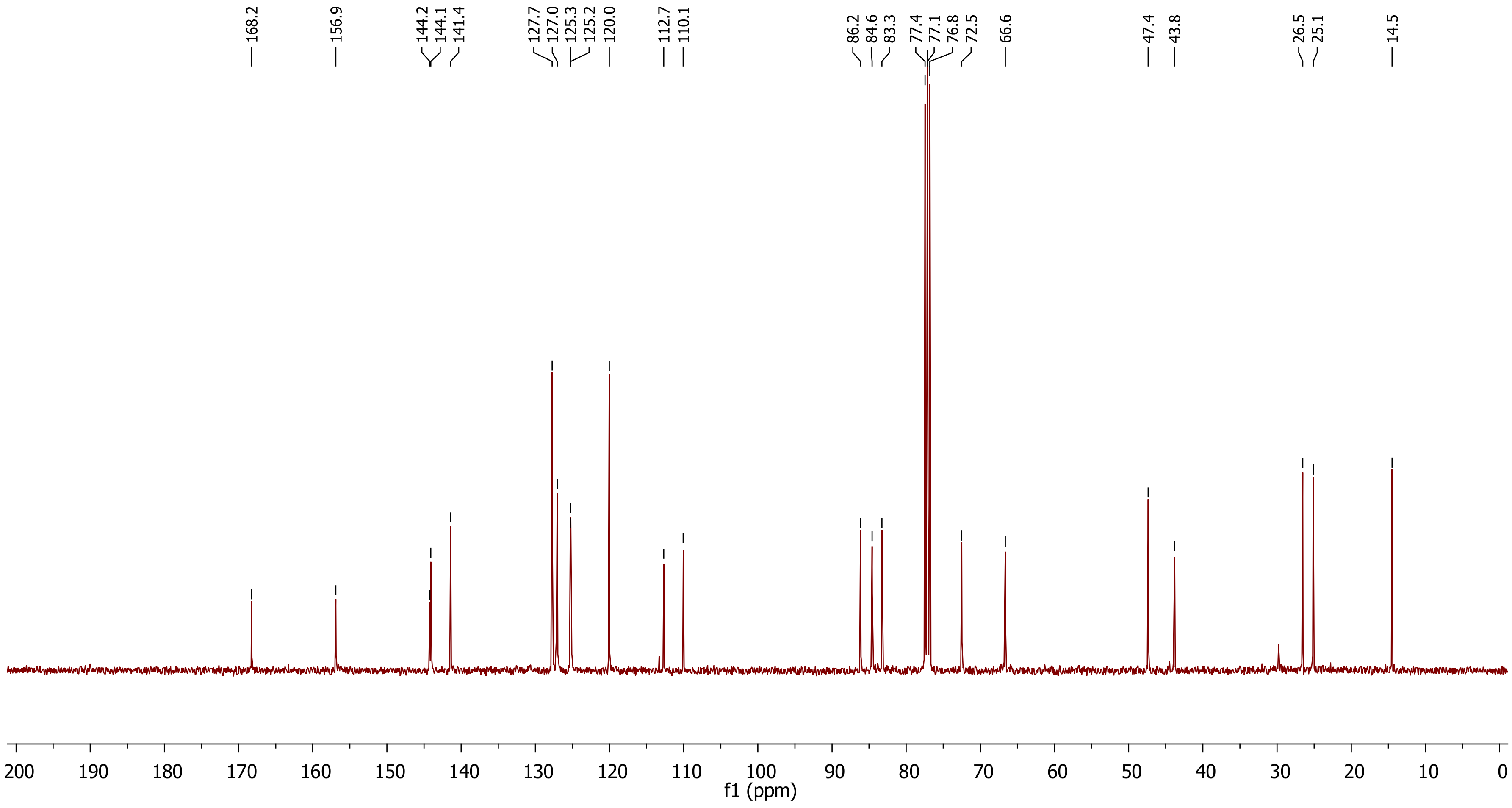

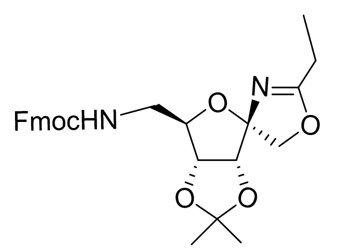

7a
$CDCl_3$, 400 MHz

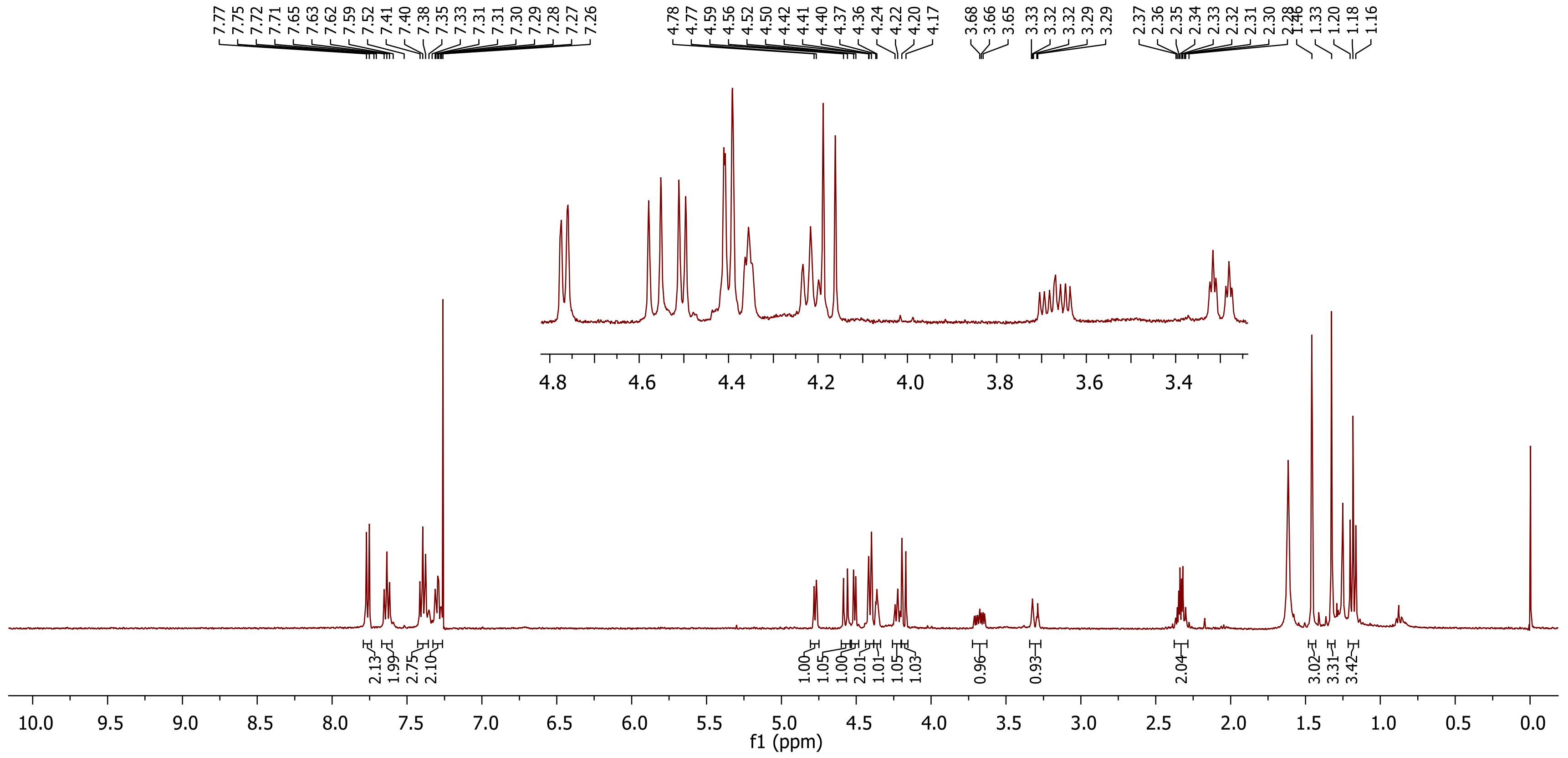

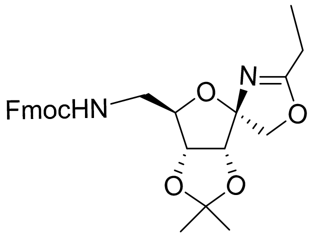

7a

$CDCl_3$, 100 MHz

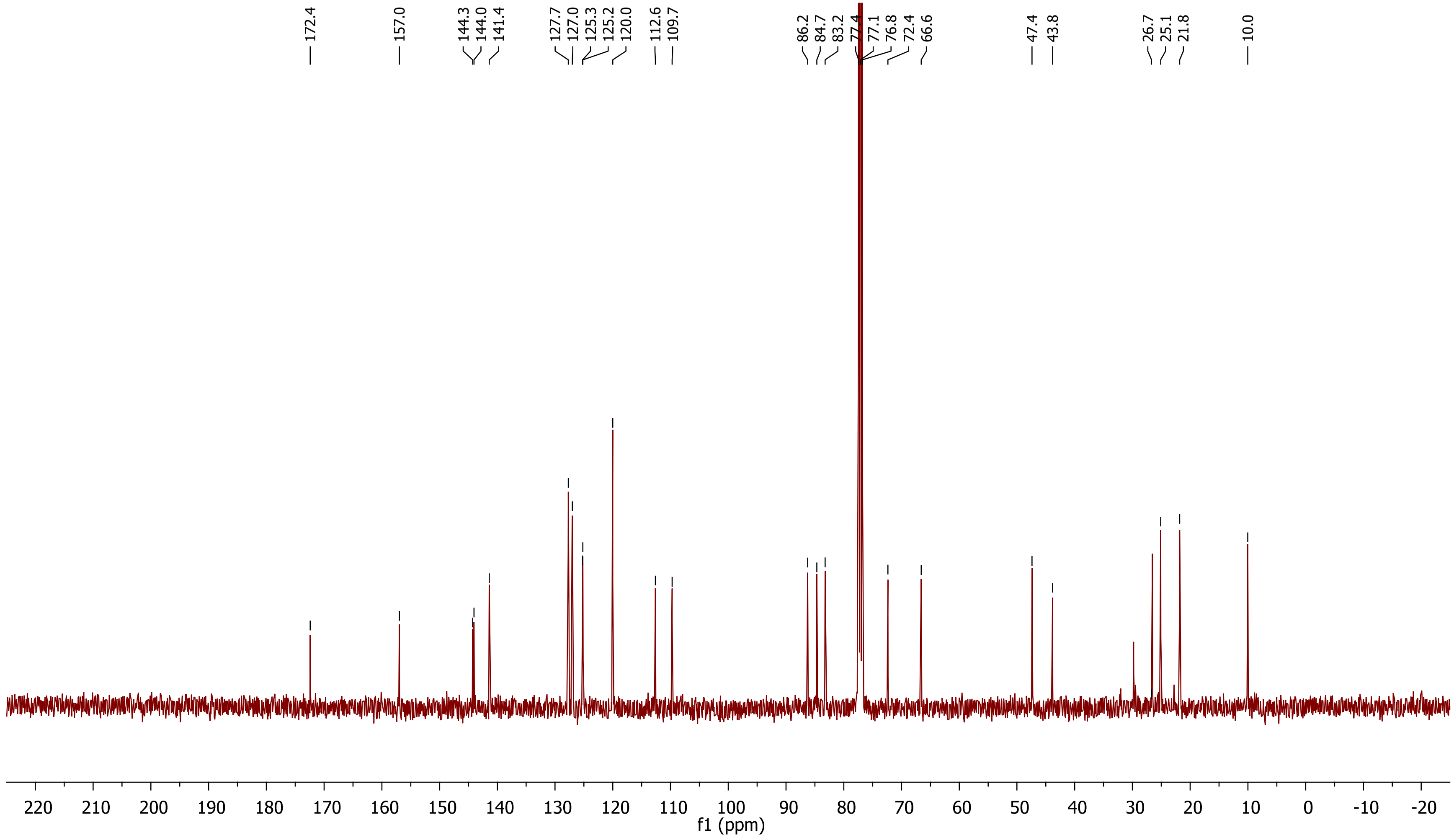

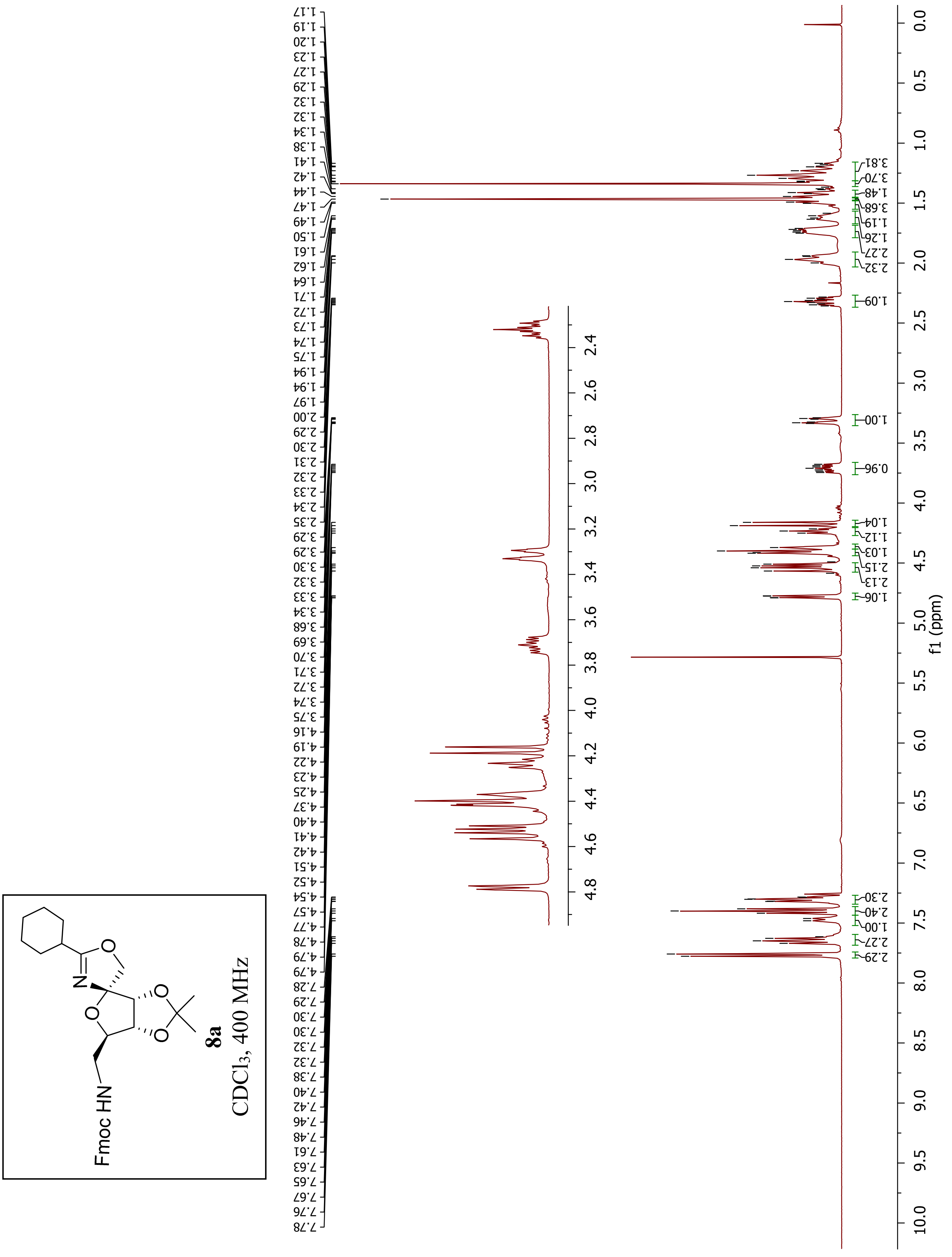

8a
$CDCl_3$, 400 MHz

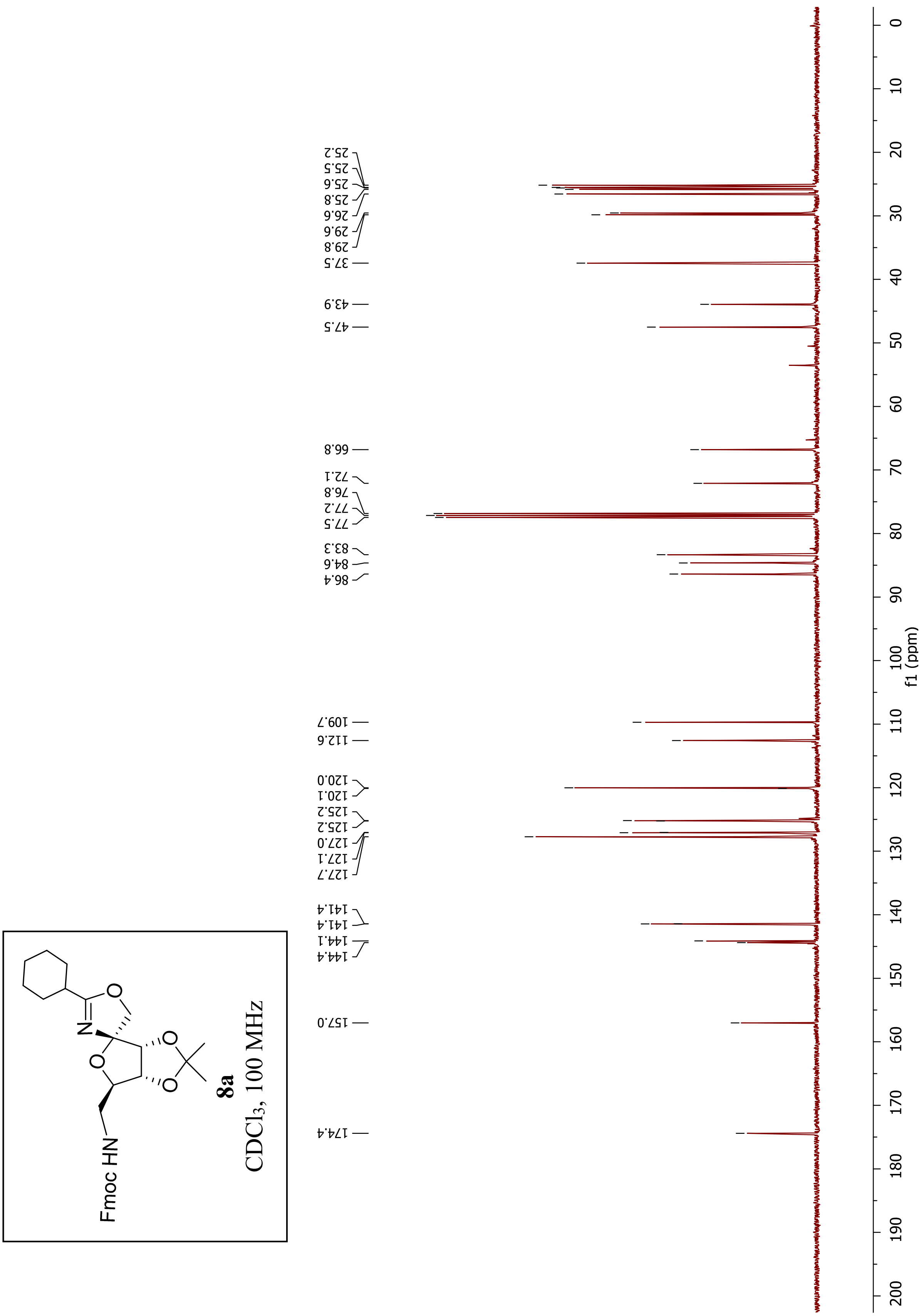
Fmoc HN
8a
CDCl3, 100 MHz
174.4
157.0
144.4
144.1
141.4
141.4
127.7
127.1
127.0
125.2
125.2
120.1
120.0
112.6
109.7
86.4
84.6
83.3
77.5
77.2
76.8
72.1
66.8
47.5
43.9
37.5
29.8
29.6
26.6
25.8
25.6
25.5
25.2
f1 (ppm)

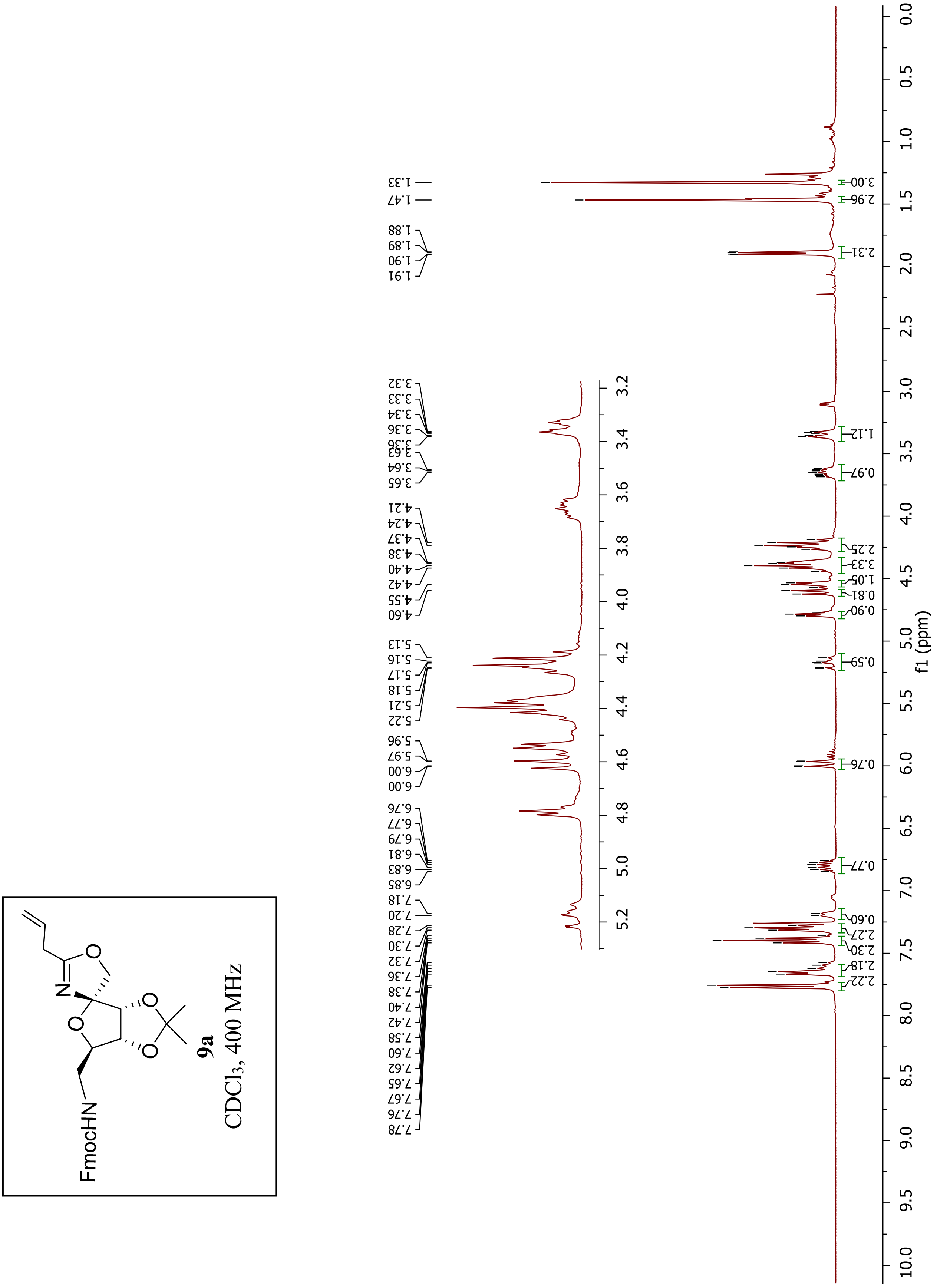
FmocHN
9a
CDCl3, 400 MHz
f1 (ppm)
1.33
1.47
1.88
1.89
1.90
1.91
3.00
2.96
2.31
1.12
0.97
2.25
3.33
1.05
0.81
0.90
0.59
0.76
0.77
0.60
2.27
2.30
2.18
2.22

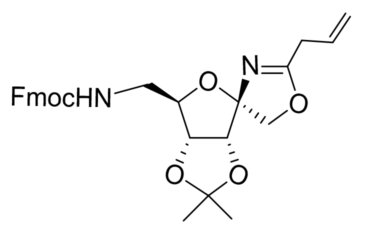

9a

CDCl3, 100 MHz

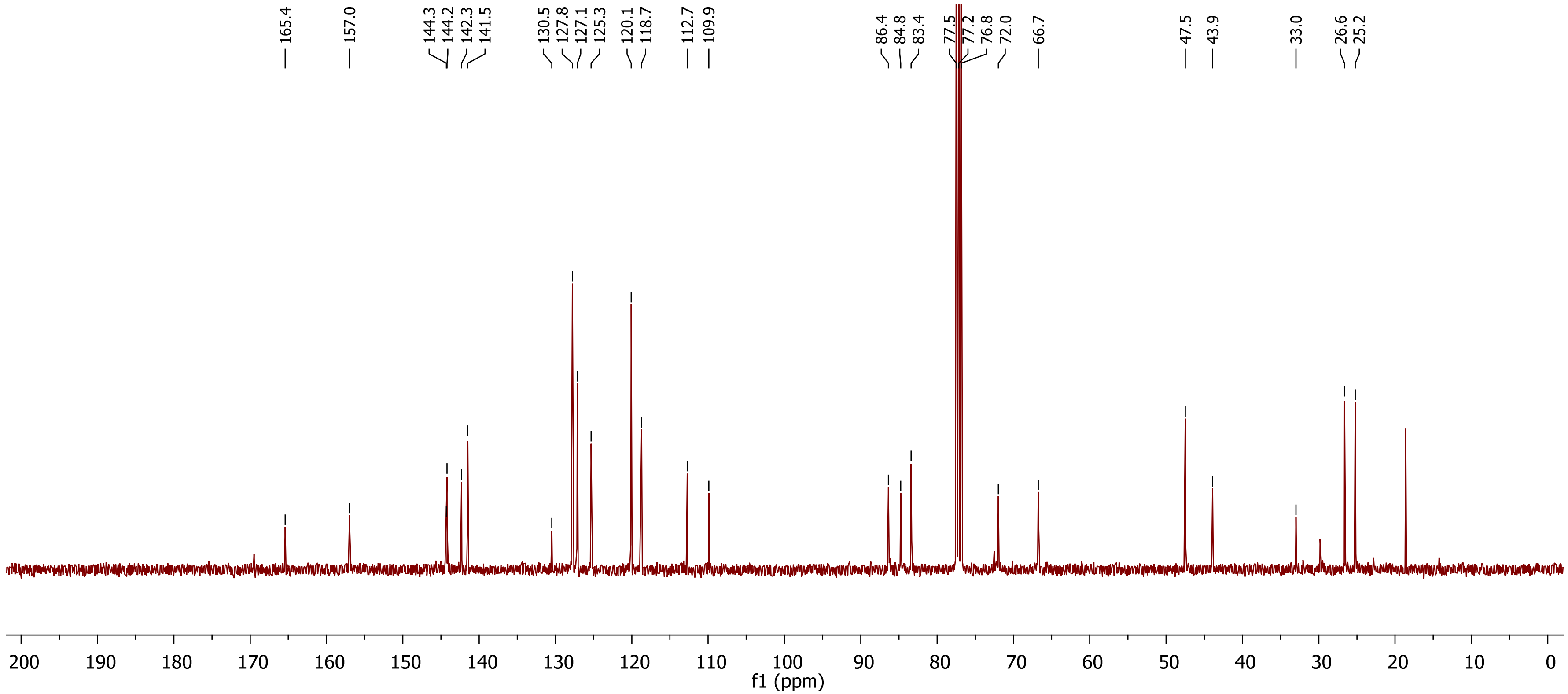

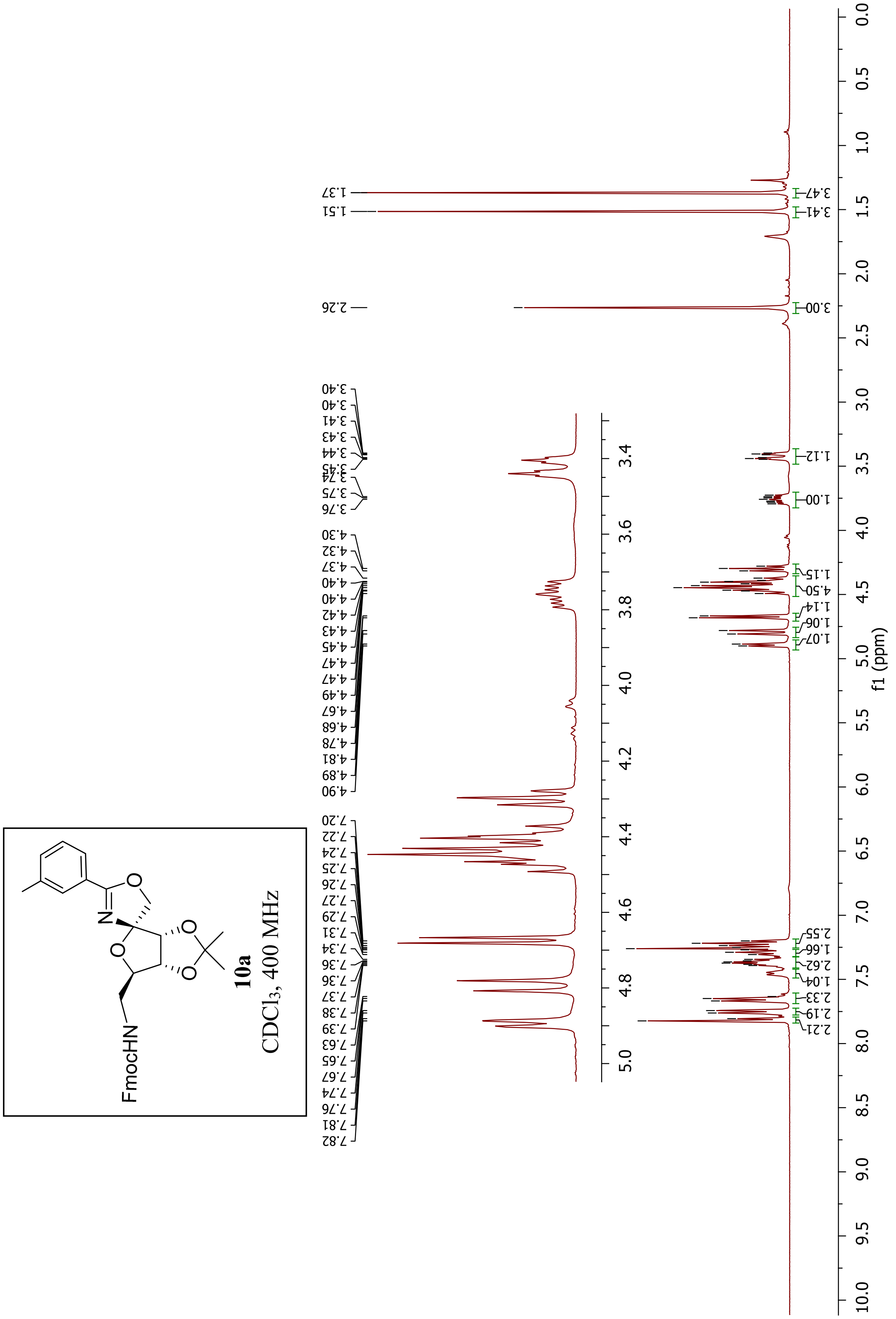
FmocHN
10a
CDCl3, 400 MHz
f1 (ppm)

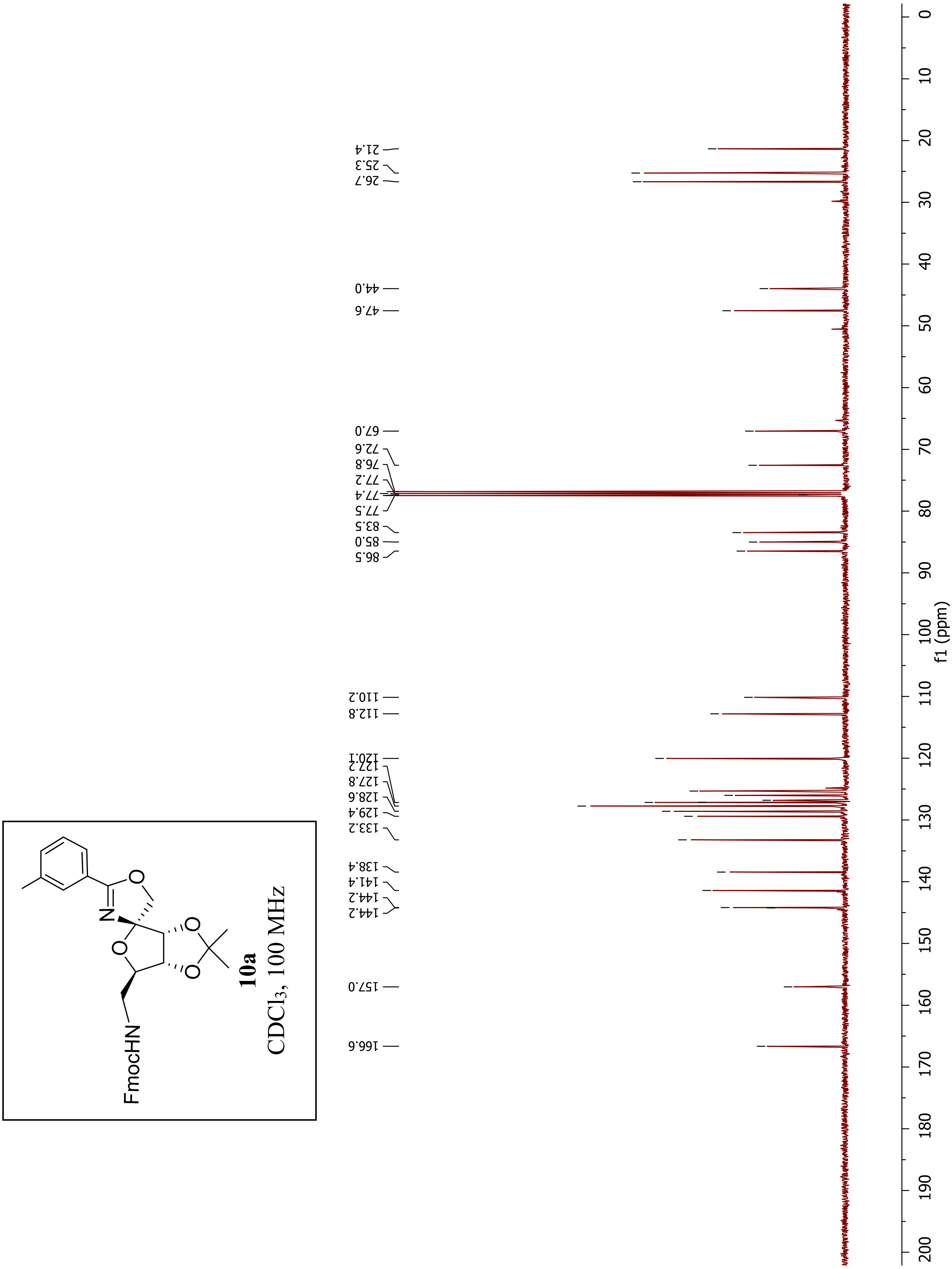
FmocHN
10a
CDCl3, 100 MHz
21.4
25.3
26.7
44.0
47.6
67.0
72.6
76.8
77.2
77.4
77.5
83.5
85.0
86.5
110.2
112.8
120.1
127.2
127.8
128.6
129.4
133.2
138.4
141.4
144.2
144.2
157.0
166.6
f1 (ppm)

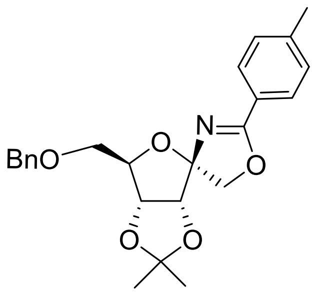

11a

$CDCl_3$, 400 MHz

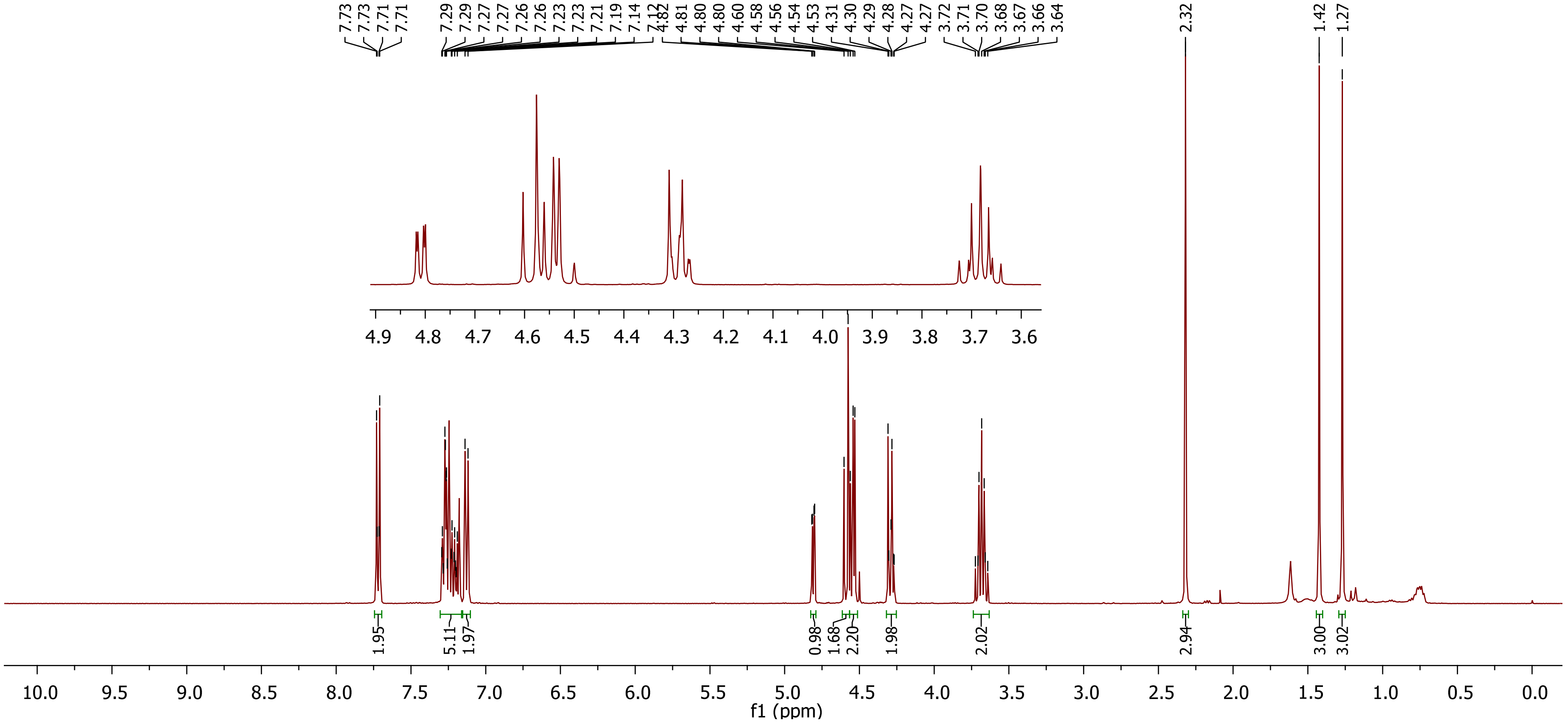

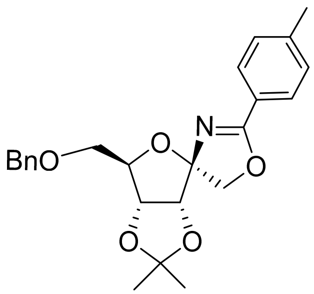

11a
$CDCl_3$, 100 MHz

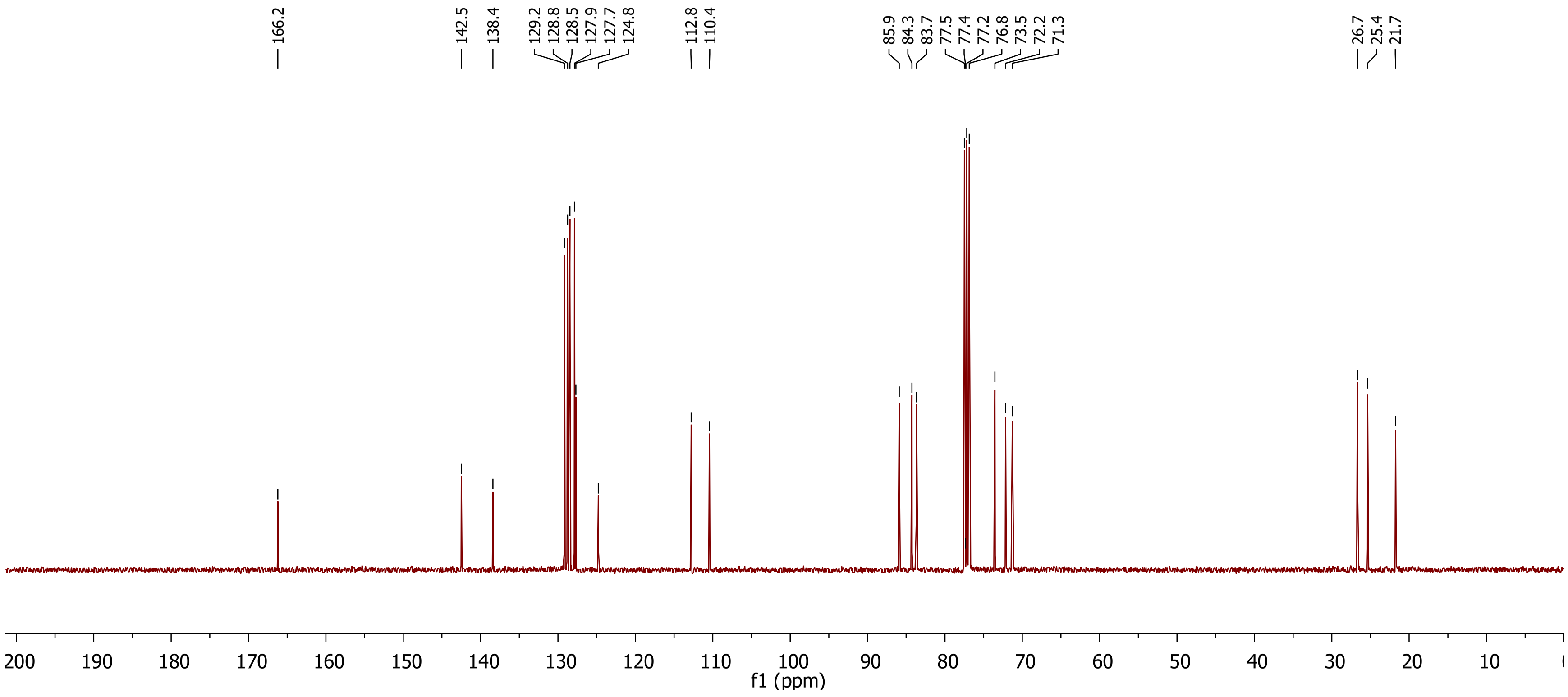

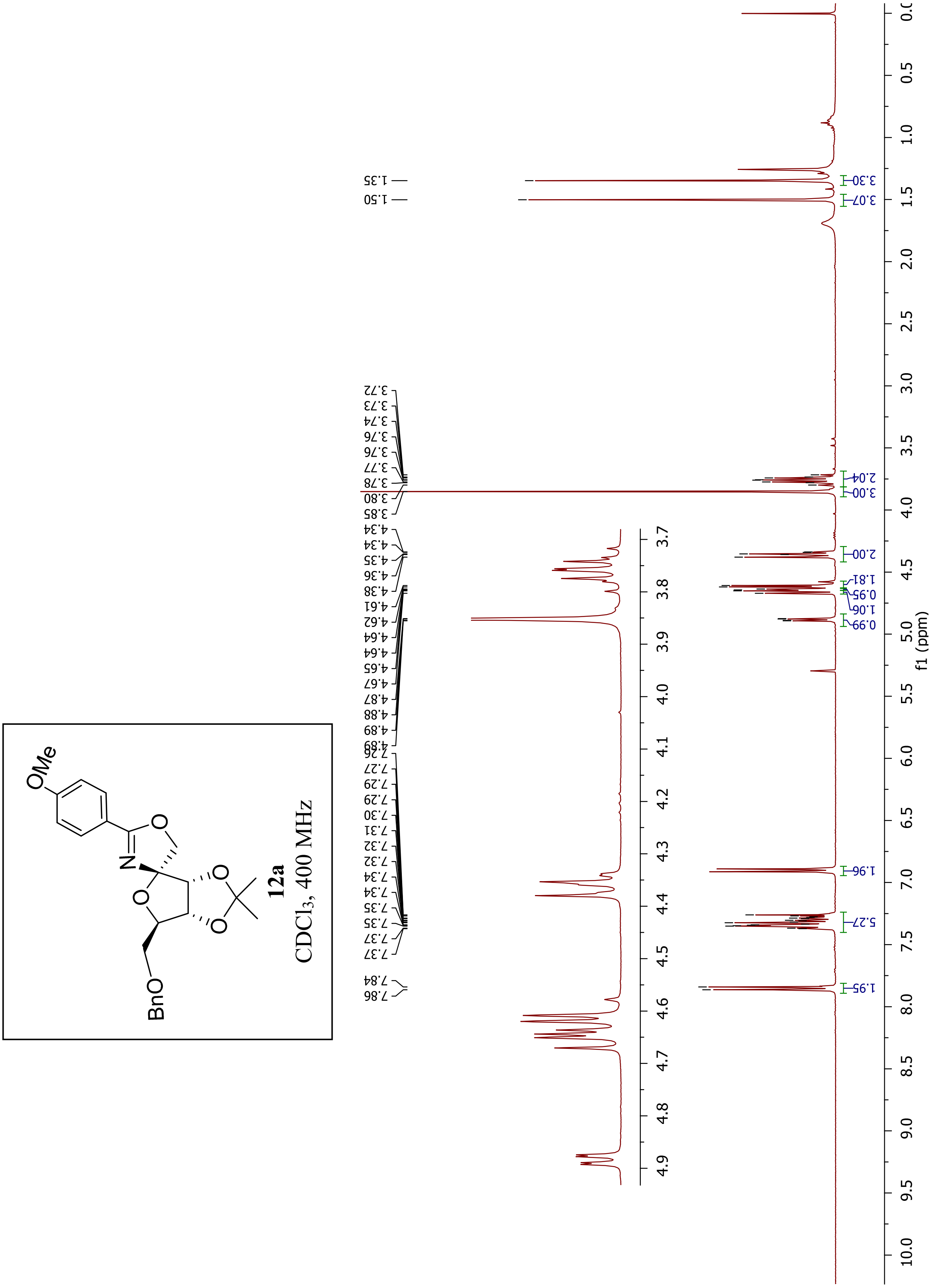
BnO
OMe
12a
CDCl3, 400 MHz
f1 (ppm)

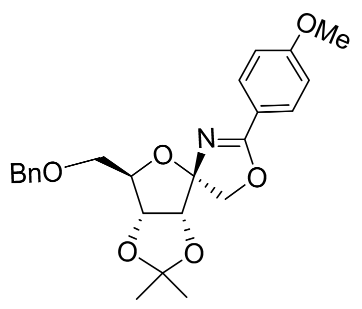

12a
$CDCl_3$, 100 MHz

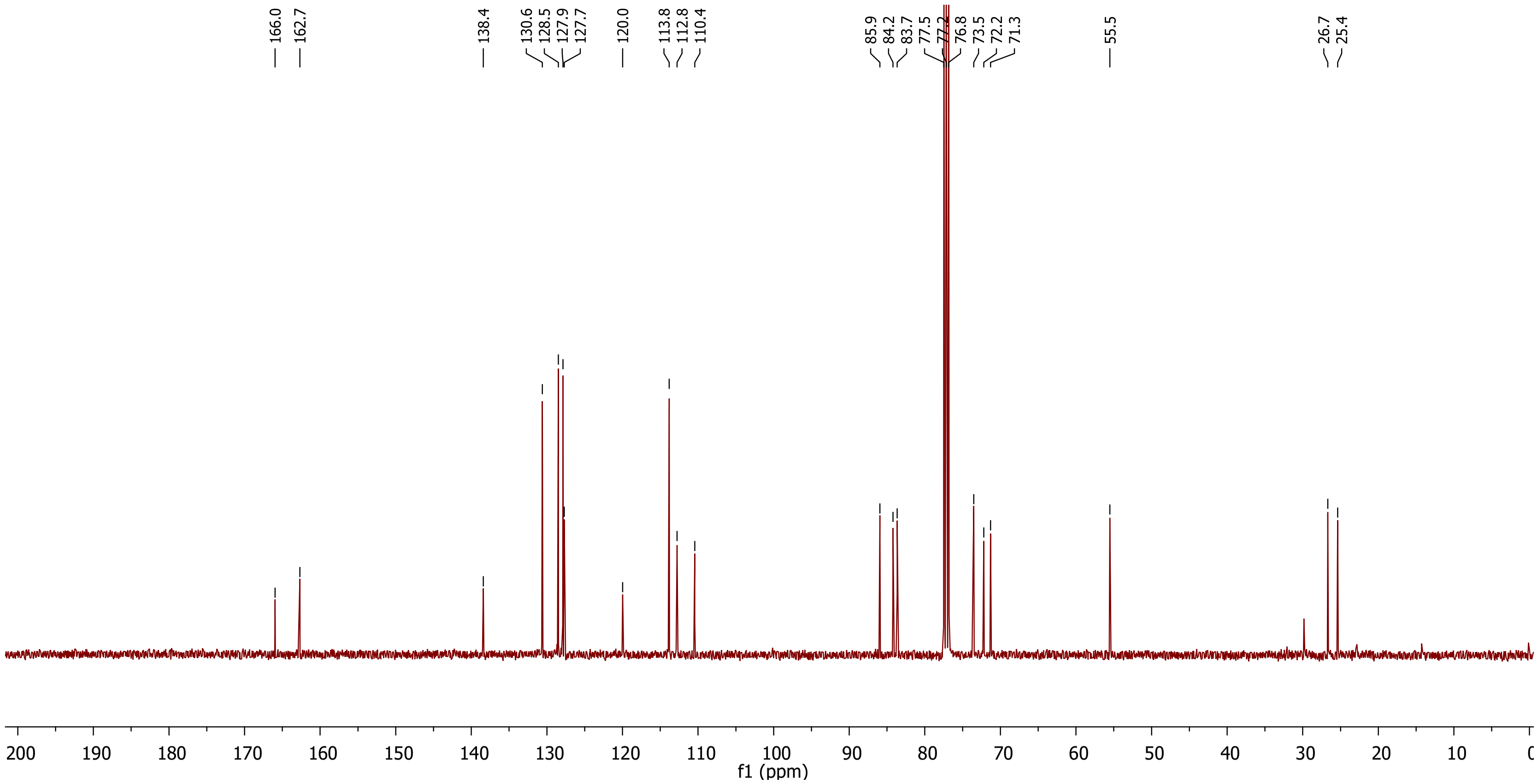

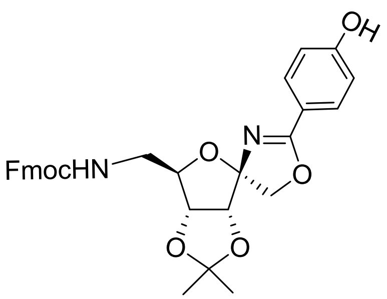

13a
$CDCl_3$, 400 MHz

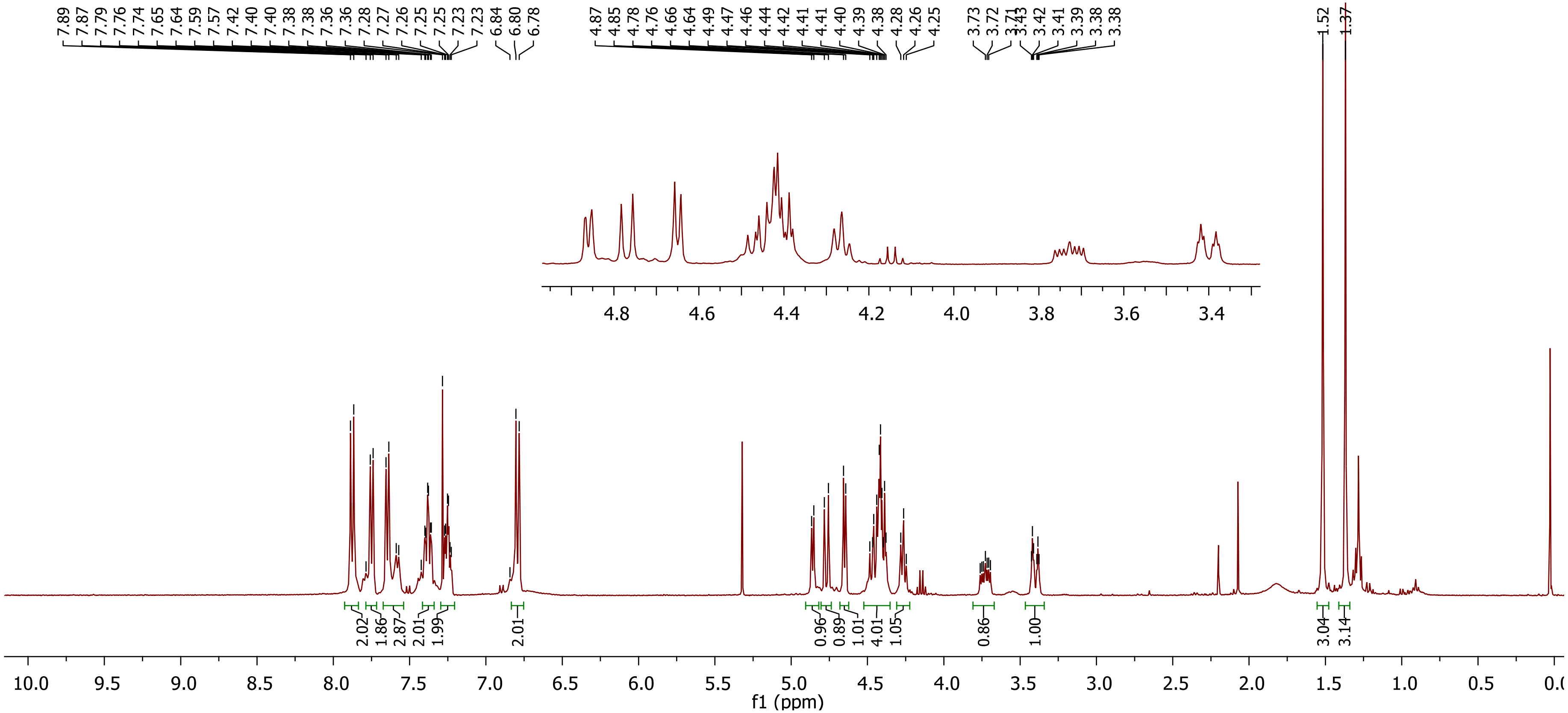

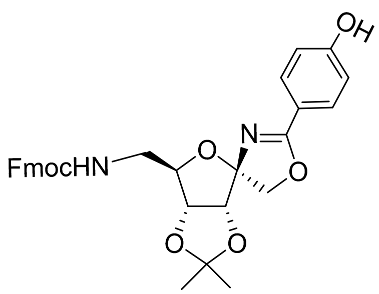

13a

$CDCl_3$, 100 MHz

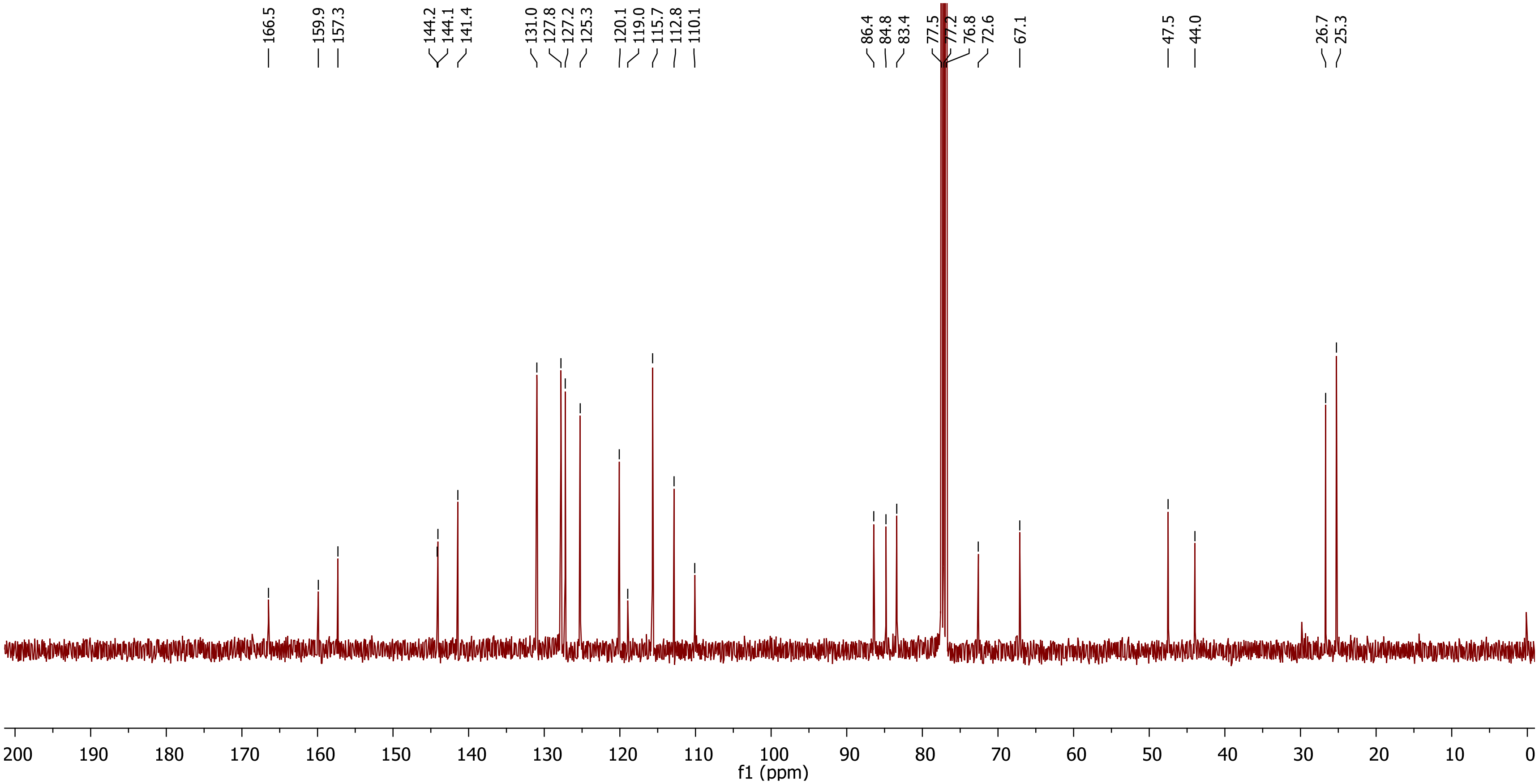

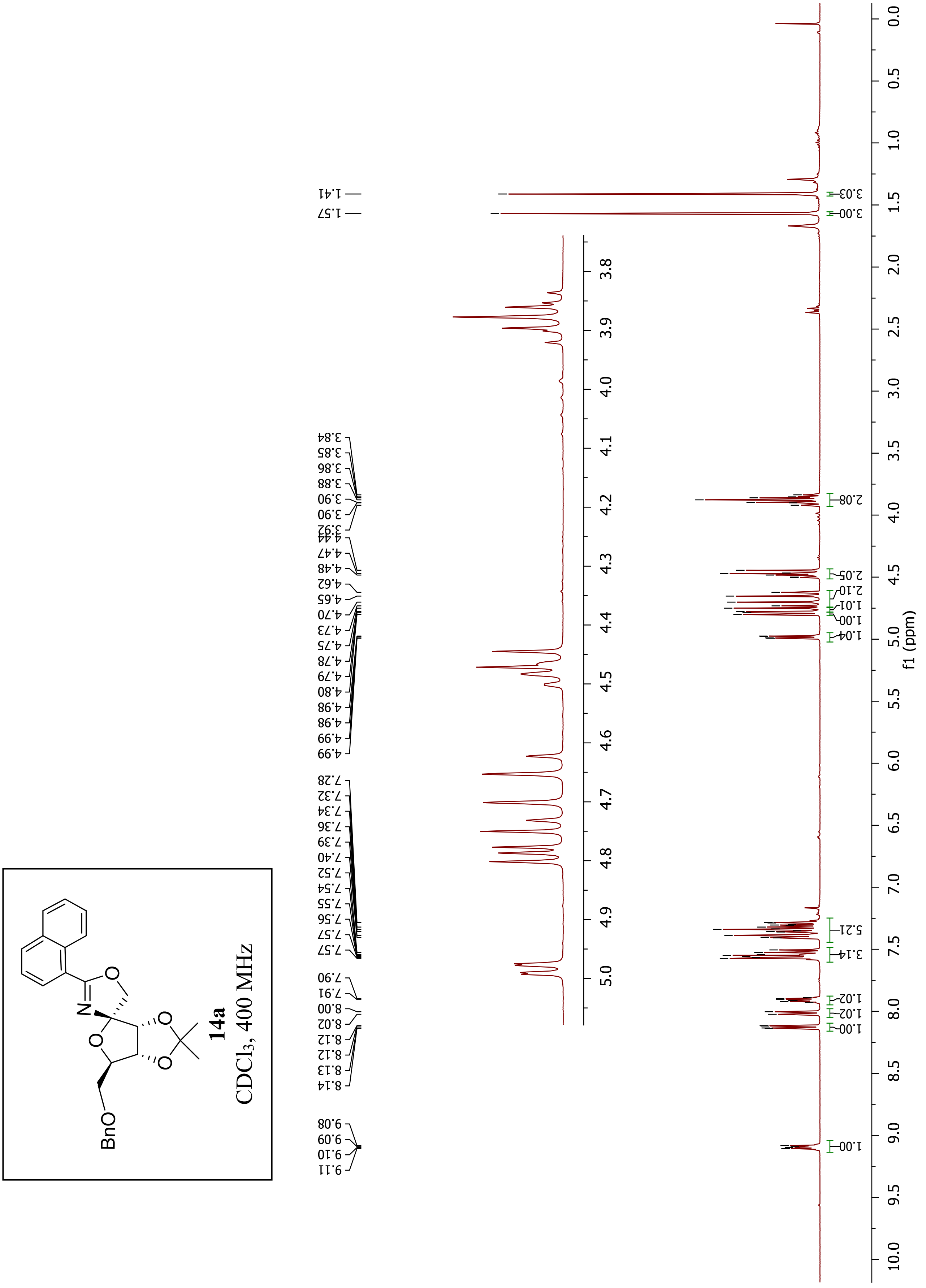
BnO
14a
CDCl3, 400 MHz
f1 (ppm)
1.41
1.57
3.84
3.85
3.86
3.88
3.90
3.90
3.92
4.44
4.47
4.48
4.62
4.65
4.70
4.73
4.75
4.78
4.79
4.80
4.98
4.98
4.99
4.99
7.28
7.32
7.34
7.36
7.39
7.40
7.52
7.54
7.55
7.56
7.57
7.57
7.90
7.91
8.00
8.02
8.12
8.12
8.13
8.14
9.08
9.09
9.10
9.11
3.03
3.00
2.08
2.05
2.10
1.01
1.00
1.04
5.21
3.14
1.02
1.02
1.00
1.00

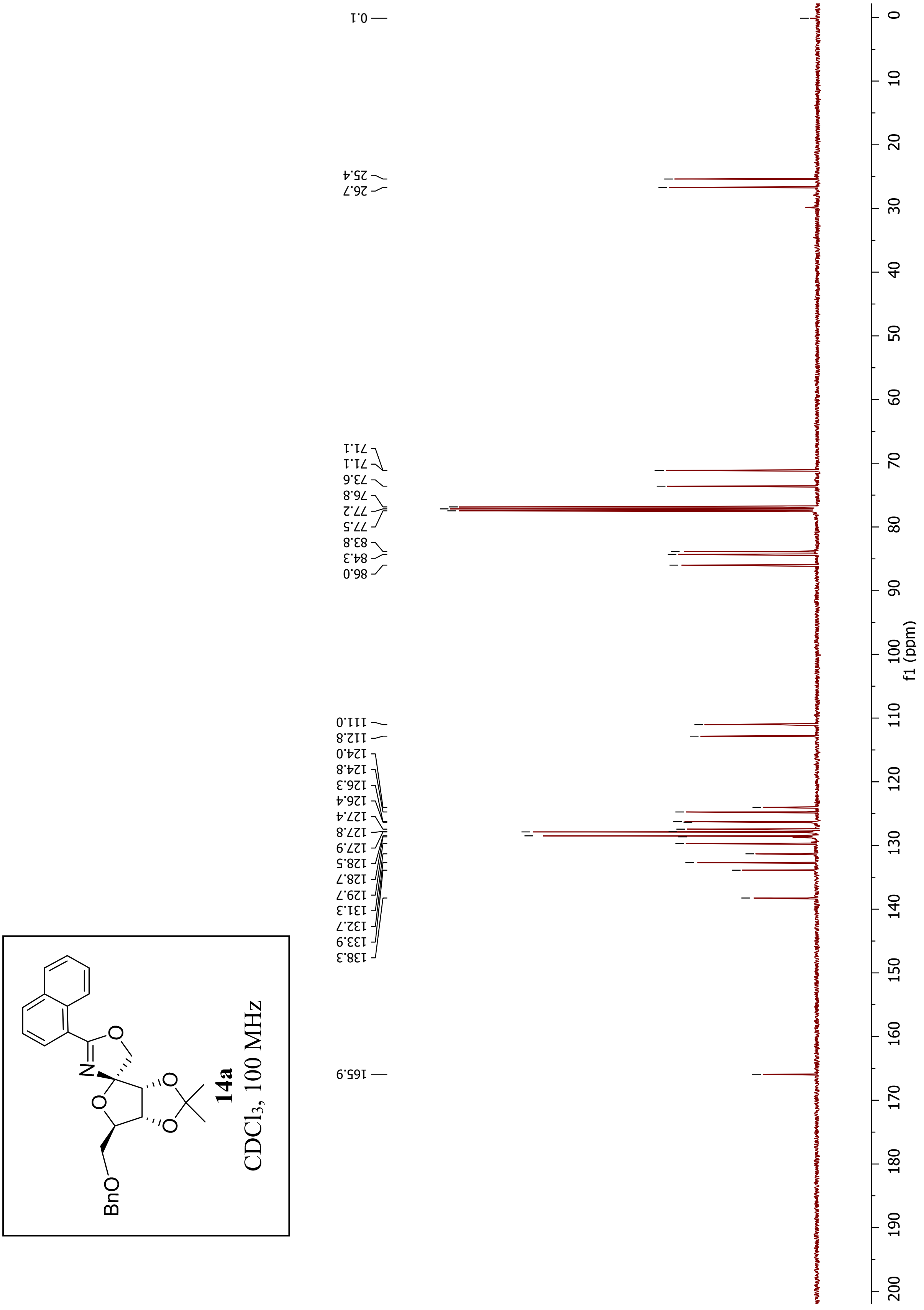
0.1
25.4
26.7
71.1
71.1
73.6
76.8
77.2
77.5
83.8
84.3
86.0
111.0
112.8
124.0
124.8
126.3
126.4
127.4
127.8
127.9
128.5
128.7
129.7
131.3
132.7
133.9
138.3
165.9
f1 (ppm)
BnO
O
N
14a
CDCl3, 100 MHz

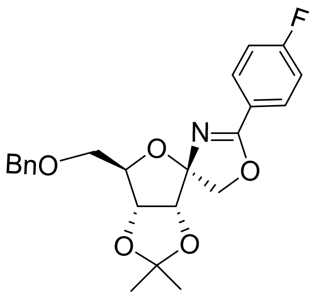

15a

$CDCl_3$, 400 MHz

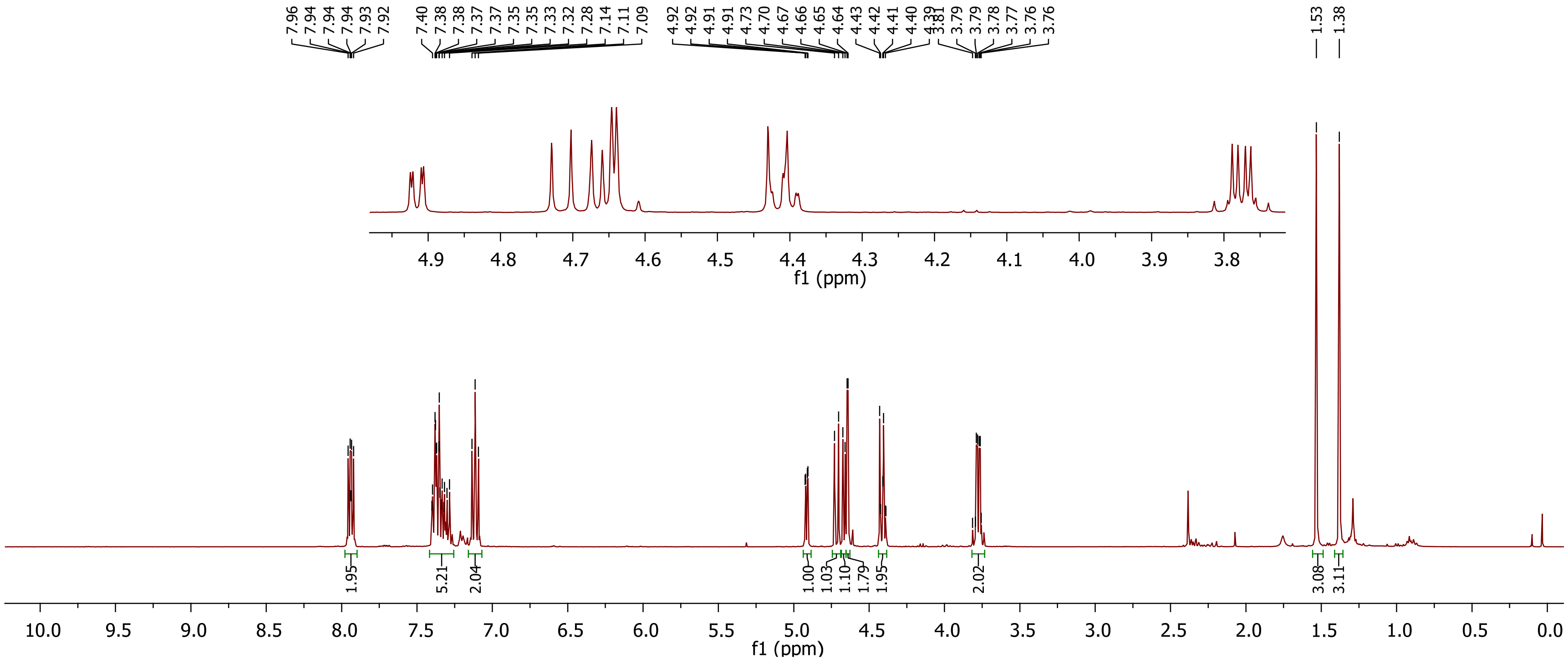

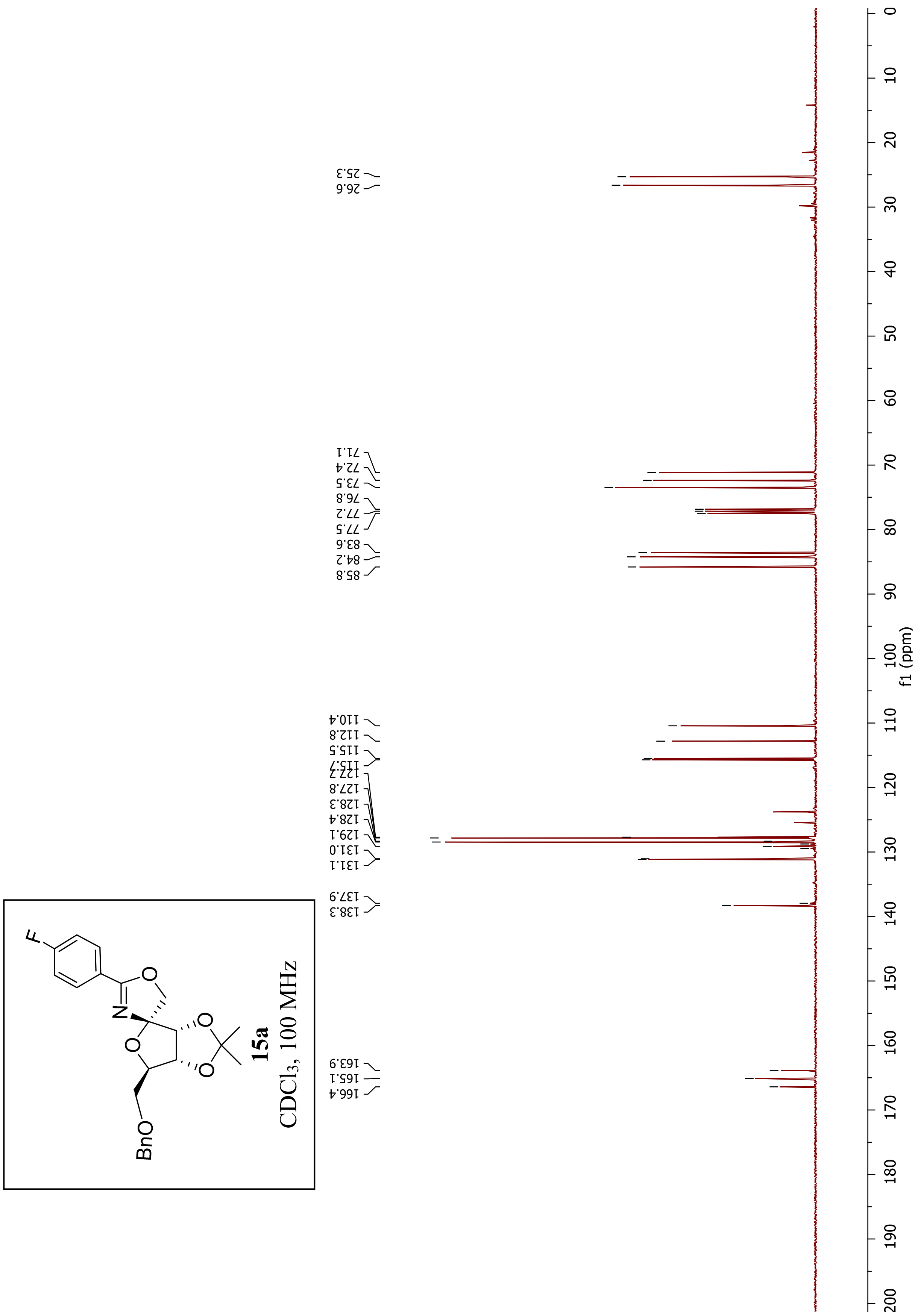
BnO
N
O
F
15a
CDCl3, 100 MHz
25.3
26.6
71.1
72.4
73.5
76.8
77.2
77.5
83.6
84.2
85.8
110.4
112.8
115.5
115.7
127.7
127.8
128.3
128.4
129.1
131.0
131.1
137.9
138.3
163.9
165.1
166.4
f1 (ppm)

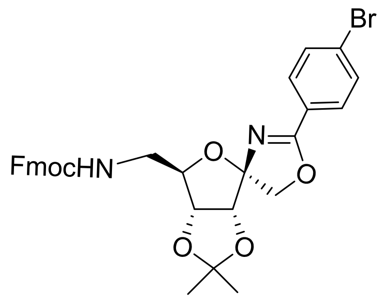

16a

$CDCl_3$, 400 MHz

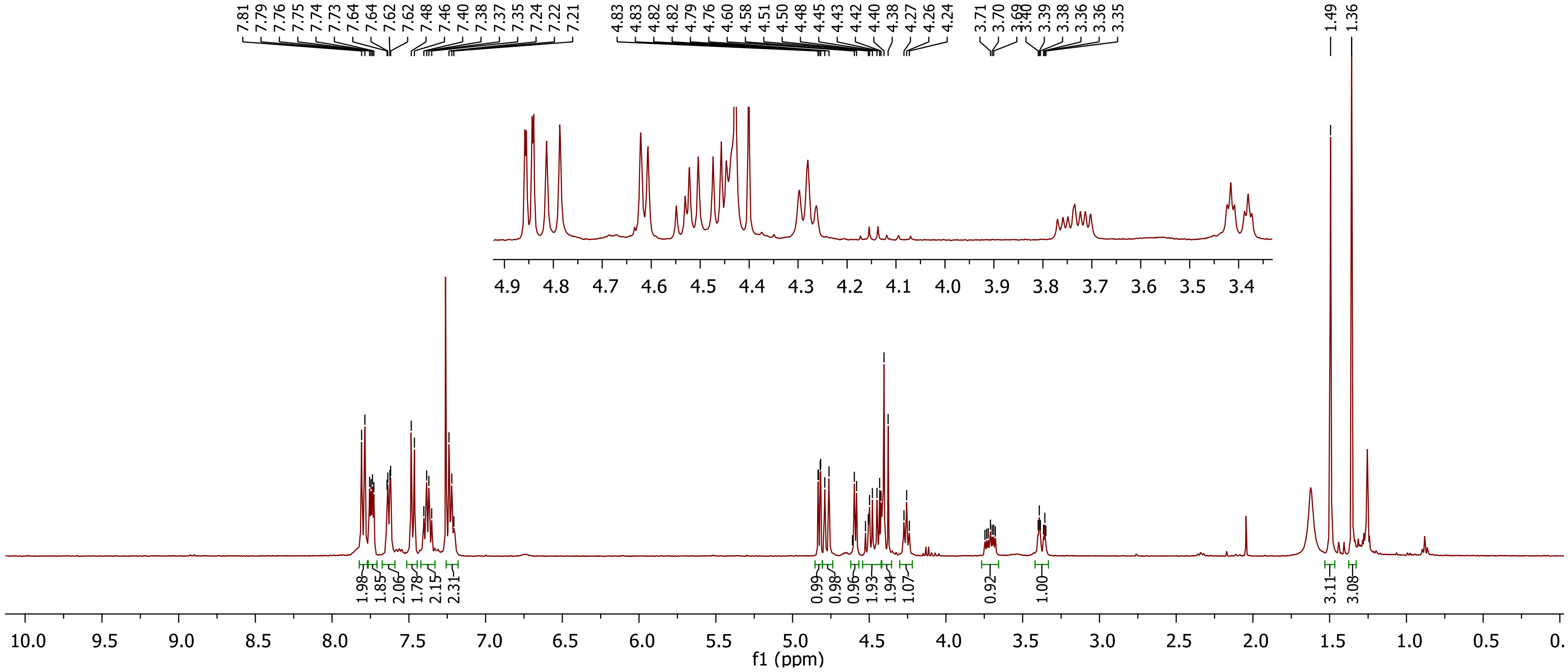

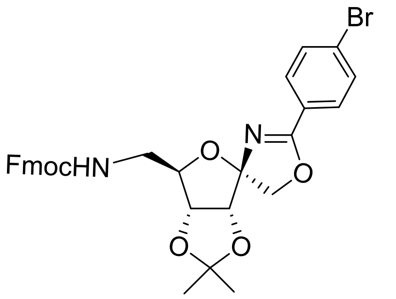

16a

$CDCl_3$, 100 MHz

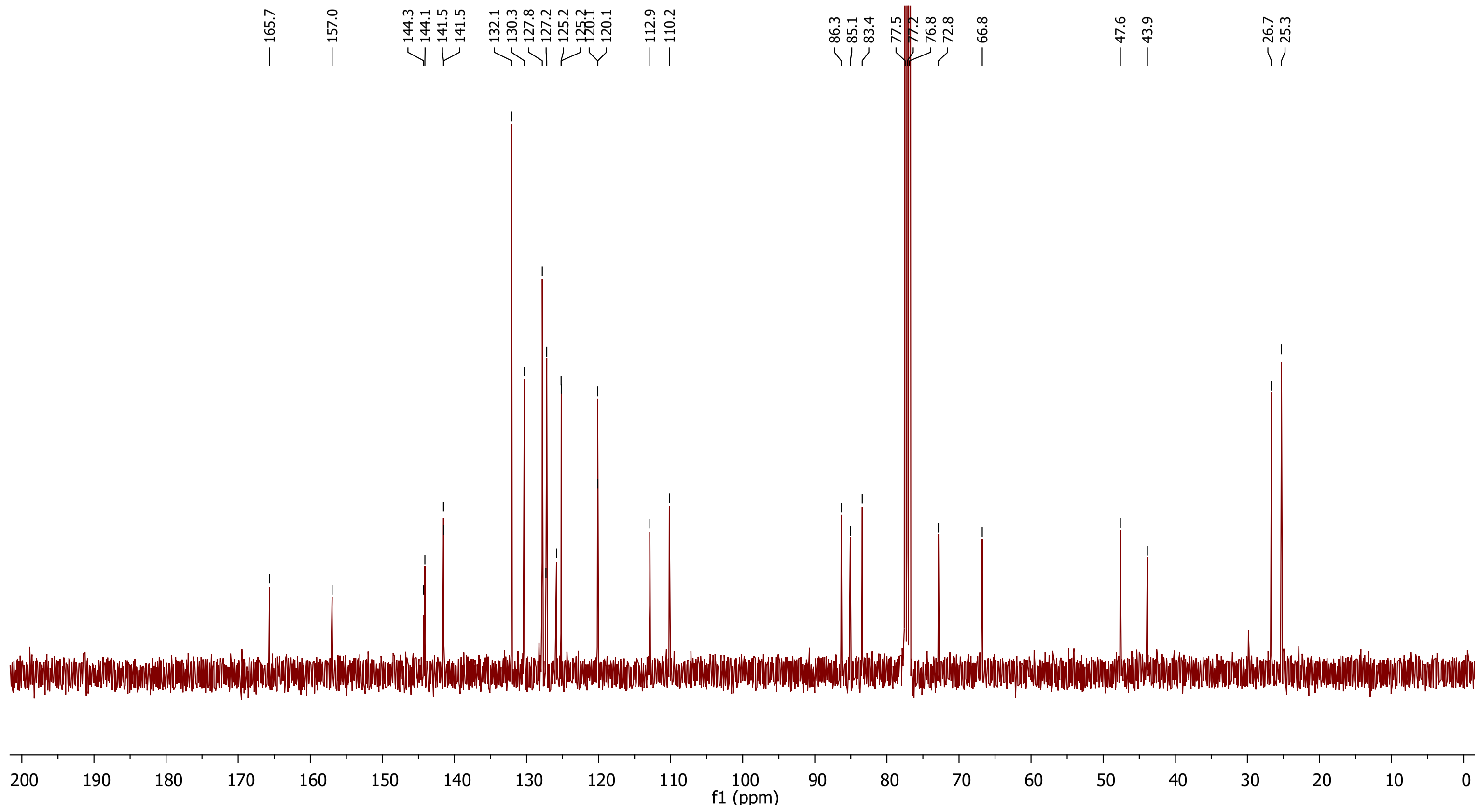

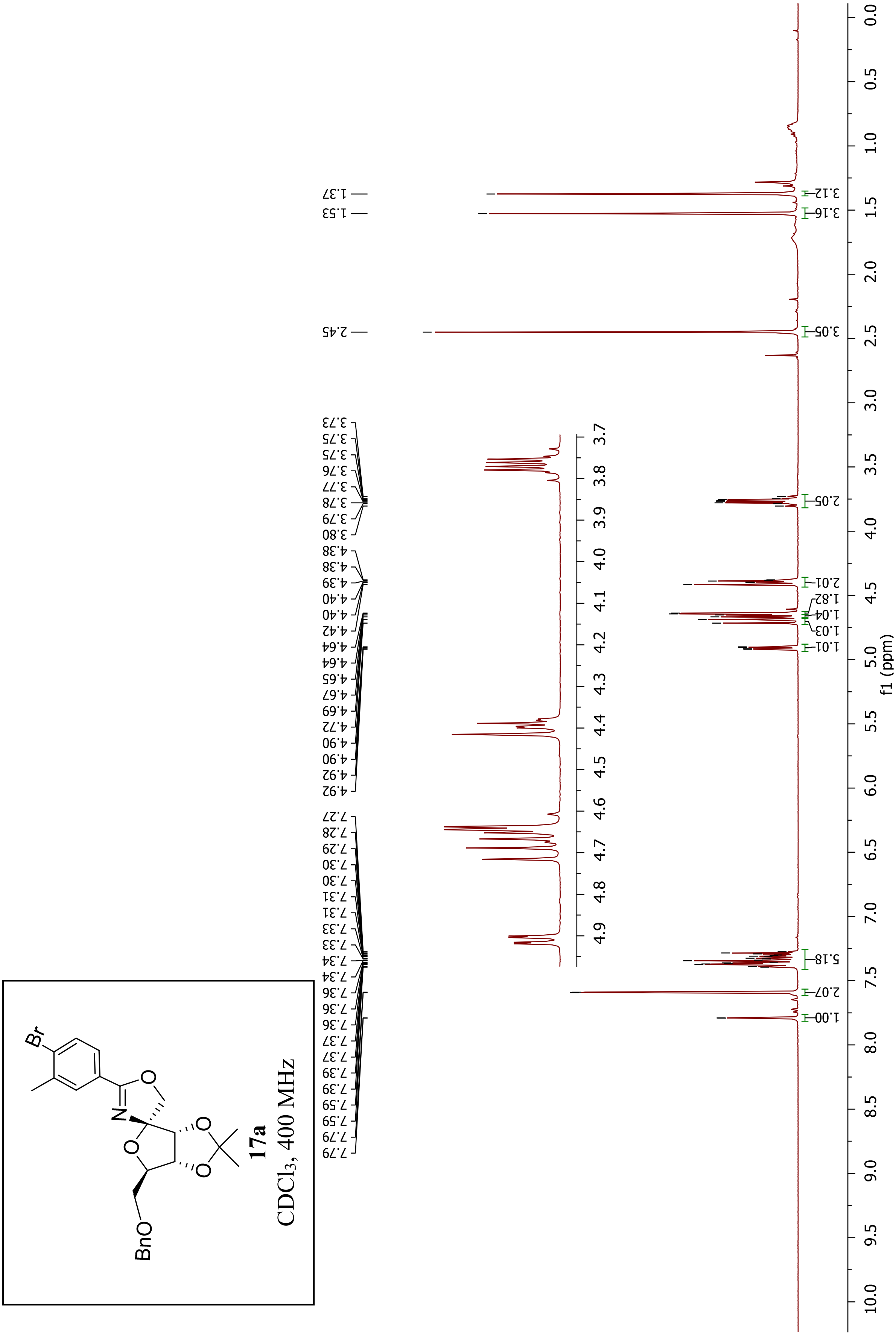
BnO
Br
N
O
17a
CDCl3, 400 MHz
f1 (ppm)

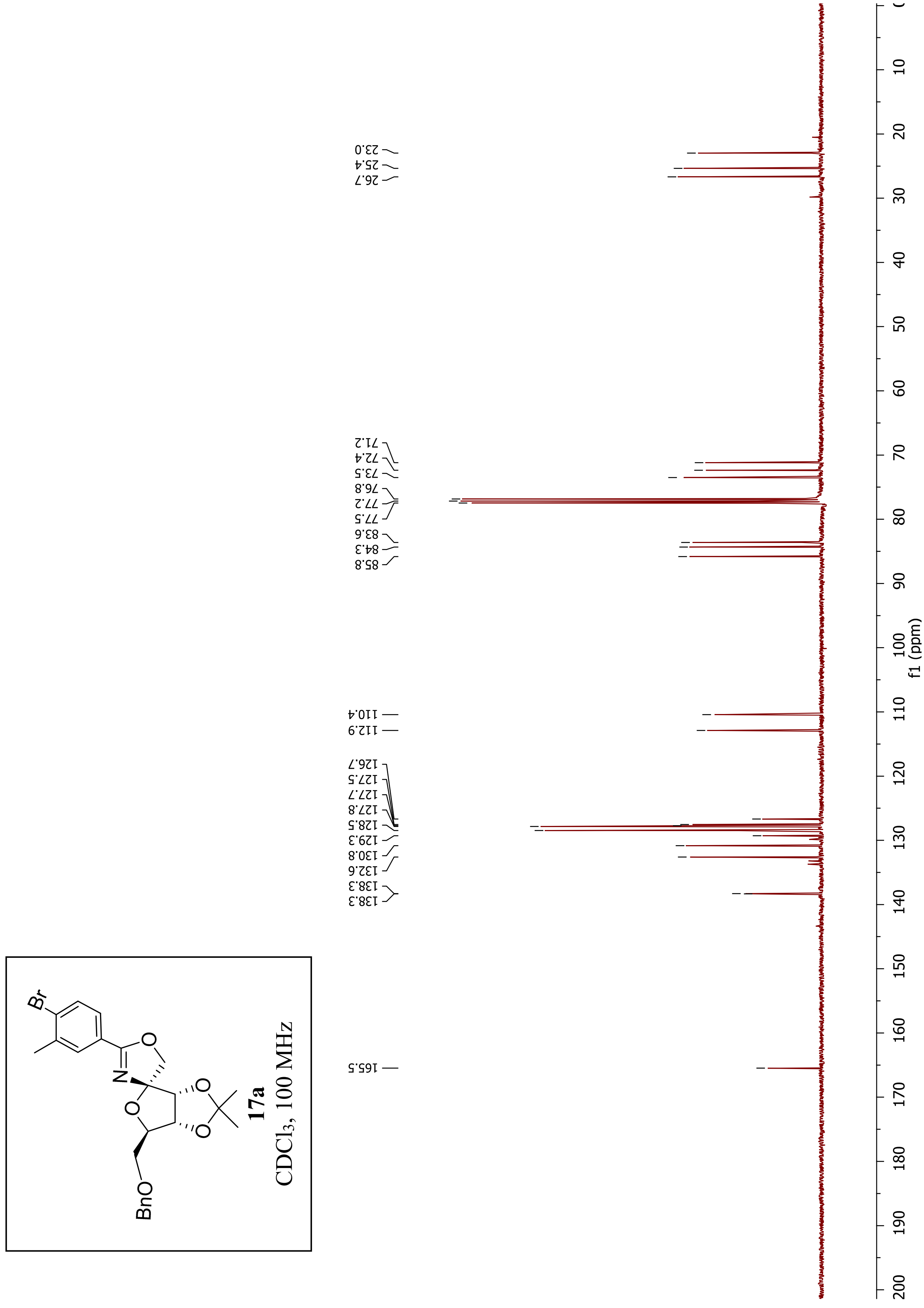

17a
$CDCl_3$, 100 MHz

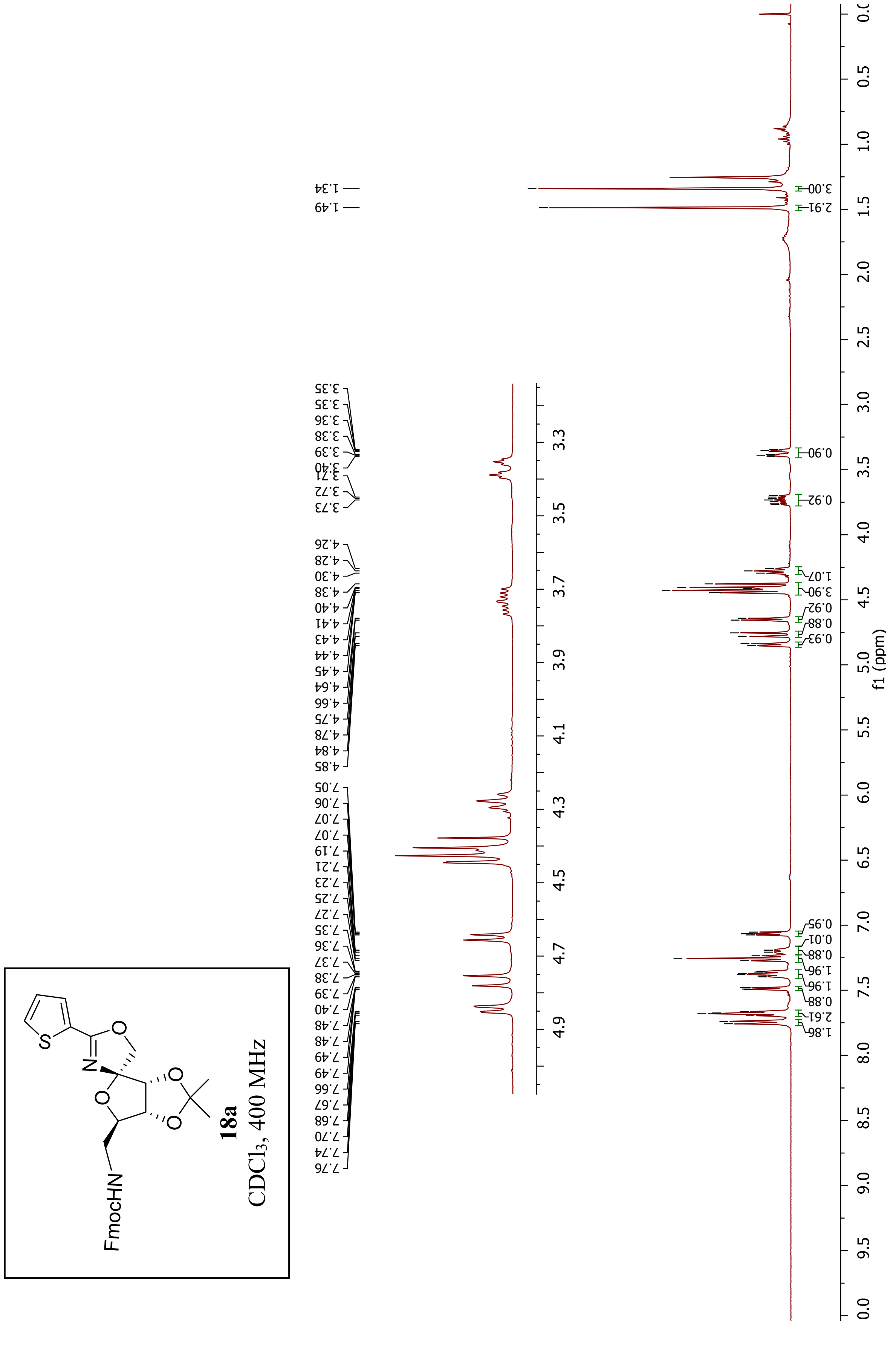
FmocHN
S
N
O
18a
CDCl3, 400 MHz
1.34
1.49
3.35
3.35
3.36
3.38
3.39
3.40
3.71
3.72
3.73
4.26
4.28
4.30
4.38
4.40
4.41
4.43
4.44
4.45
4.64
4.66
4.75
4.78
4.84
4.85
7.05
7.06
7.07
7.07
7.19
7.21
7.23
7.25
7.27
7.35
7.36
7.37
7.38
7.39
7.40
7.48
7.48
7.49
7.49
7.66
7.67
7.68
7.70
7.74
7.76
f1 (ppm)

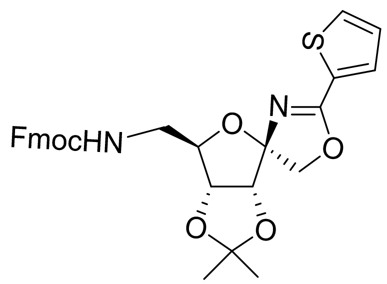

18a

$CDCl_3$, 100 MHz

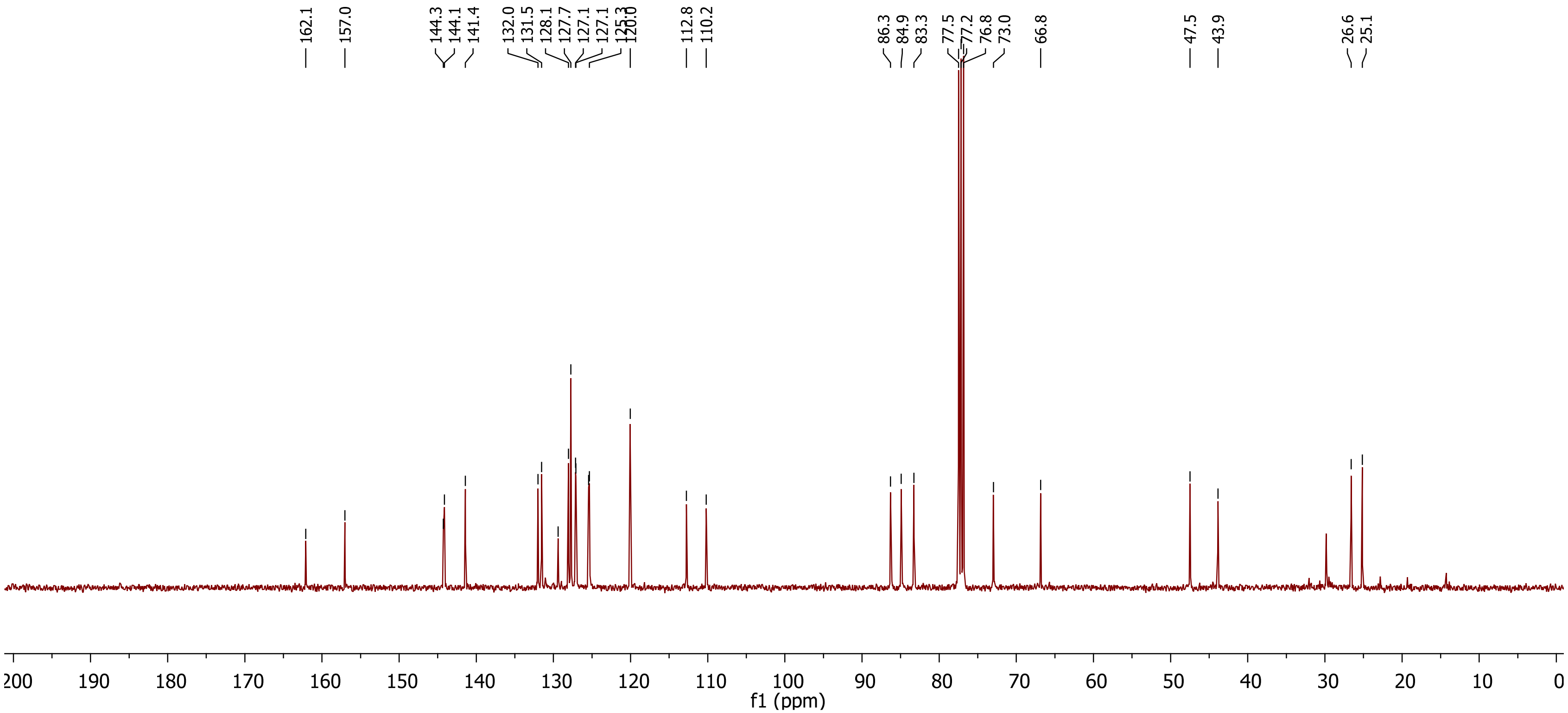

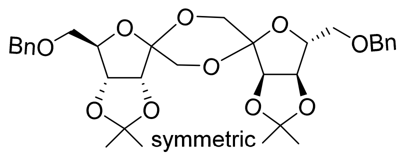

3ab

CDCl$_3$, 400 MHz

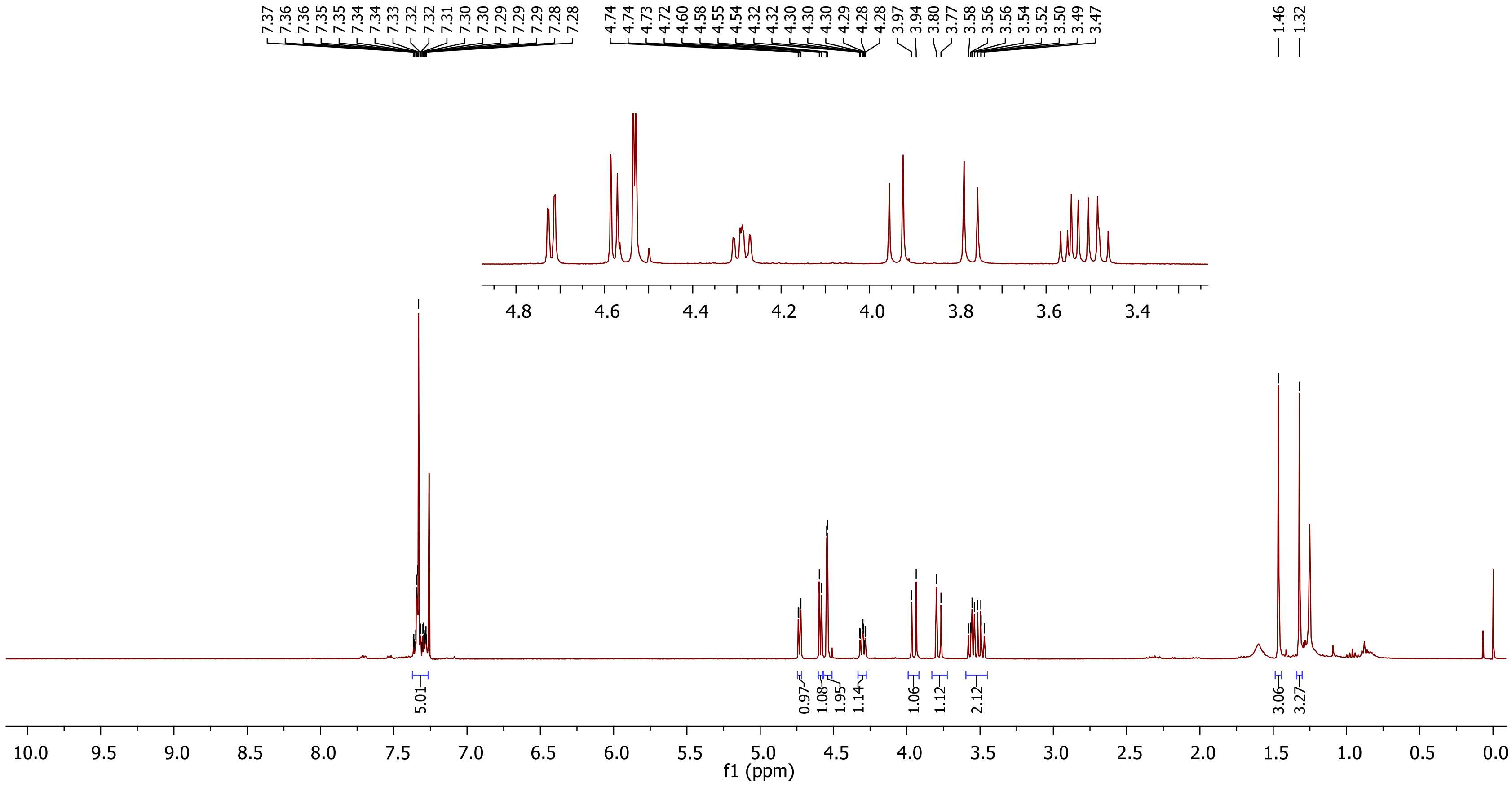

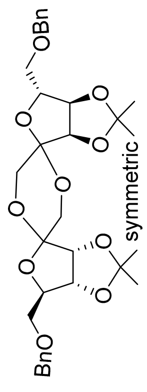

3ab
$CDCl_3$, 100 MHz

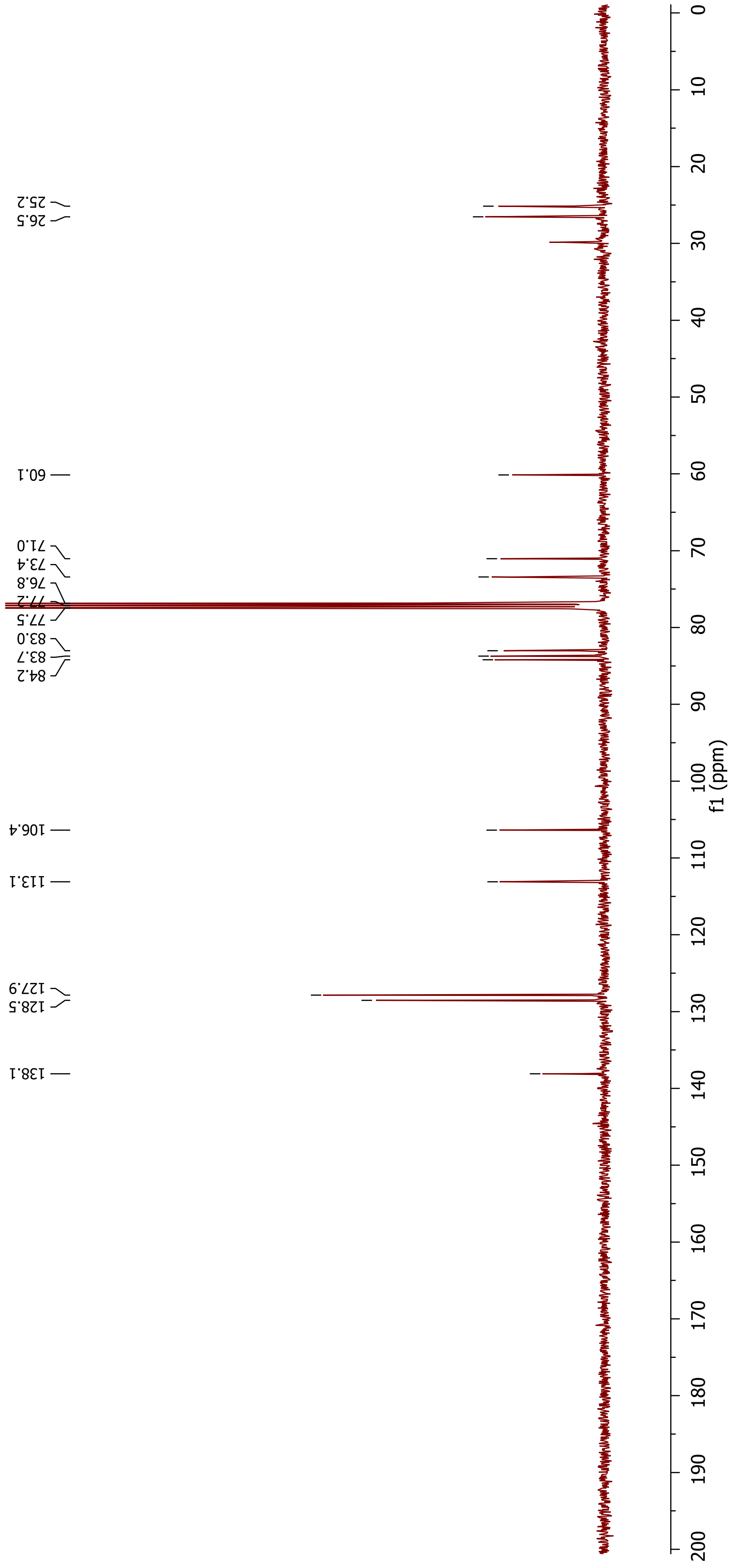

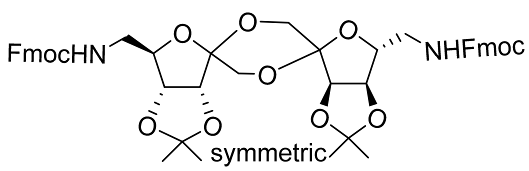

5ab, $CDCl_3$, 400 MHz

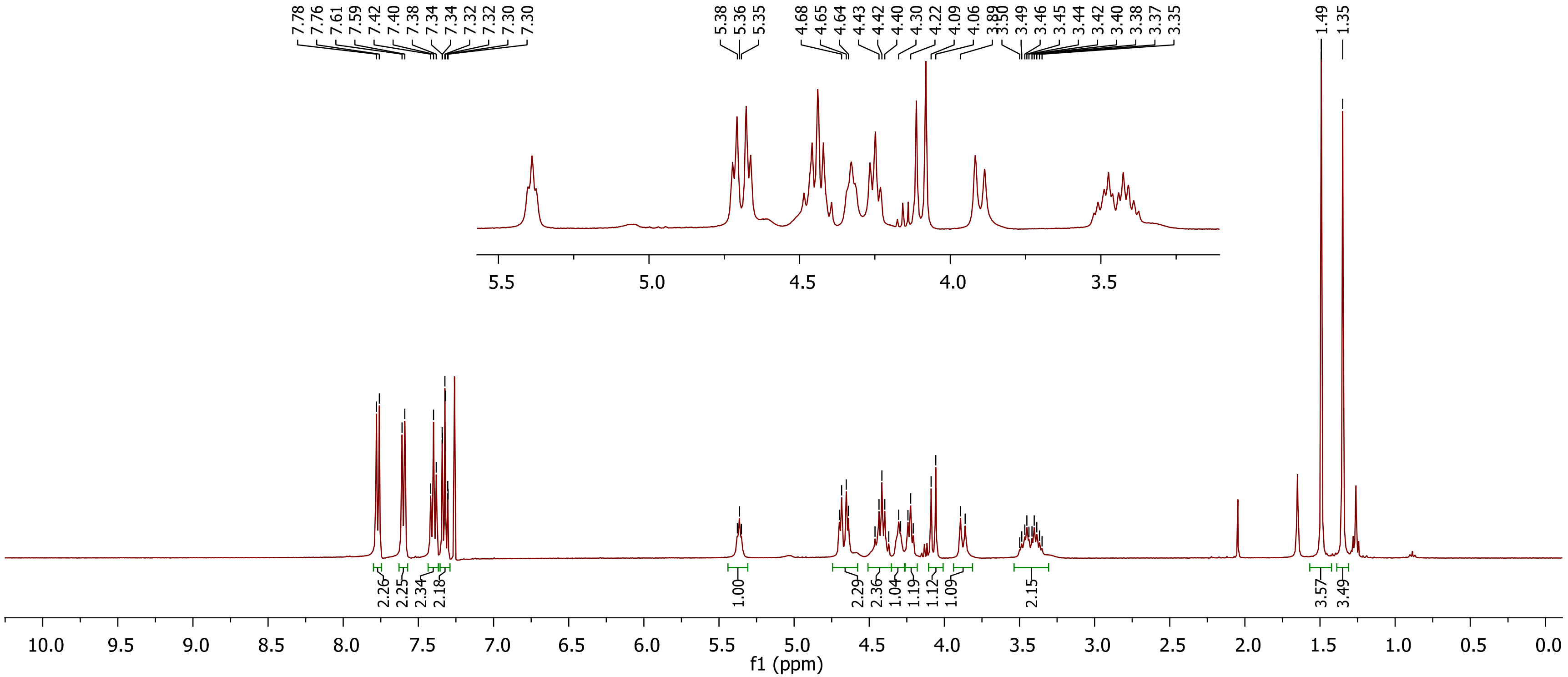

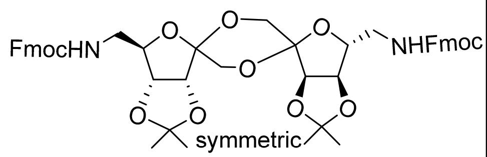

5ab, $CDCl_3$, 100 MHz

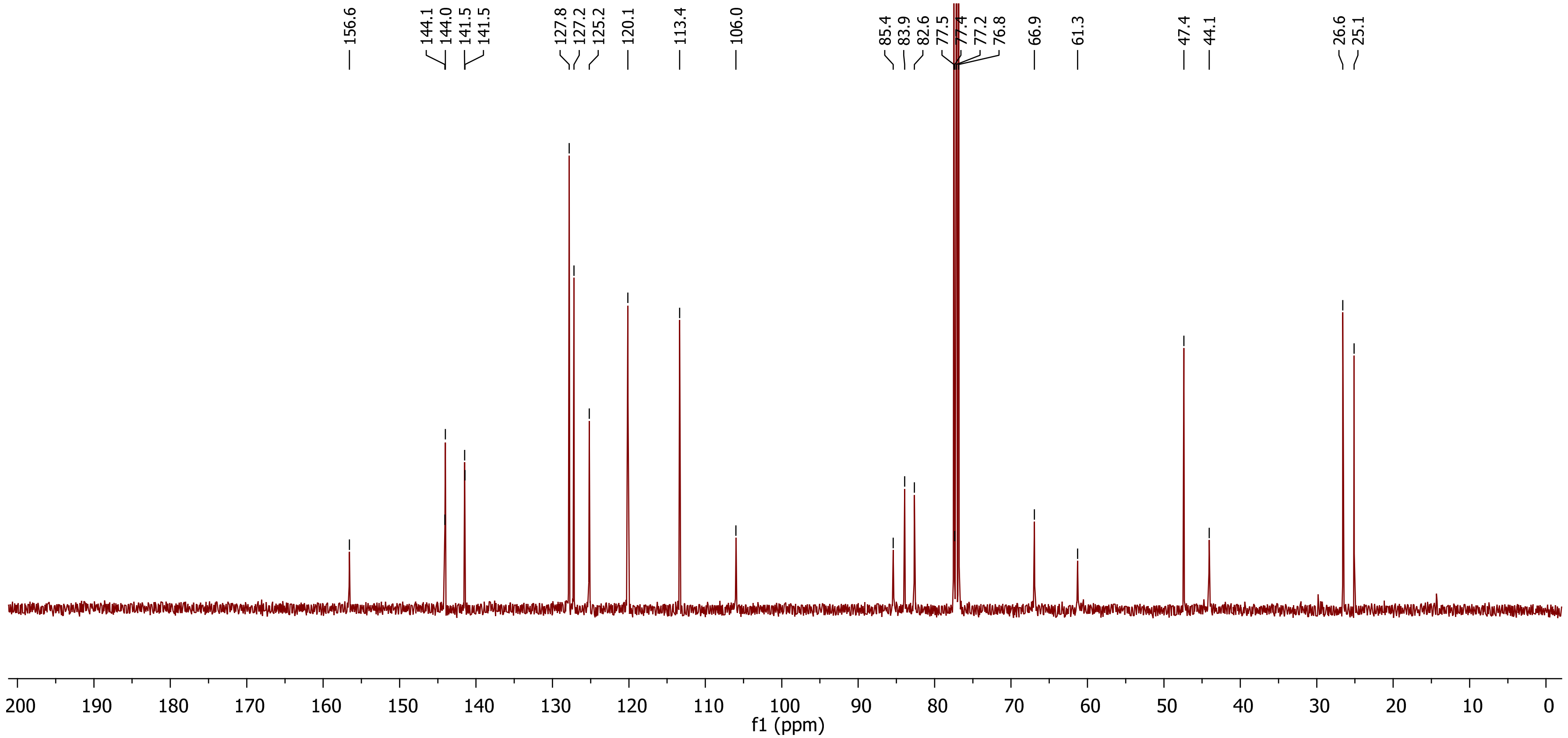

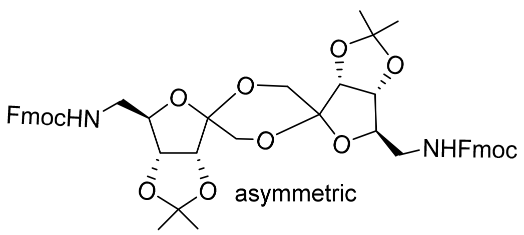

5ac, $CDCl_3$, 400 MHz

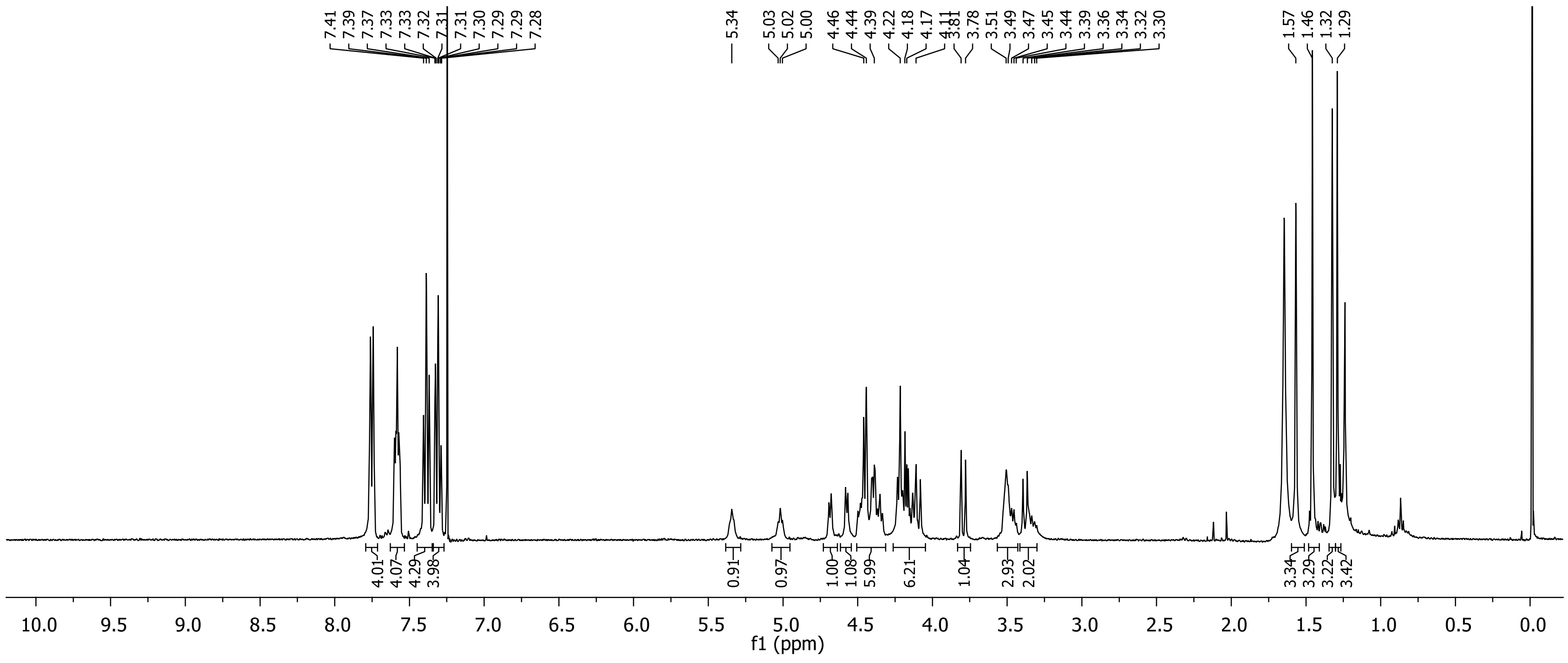

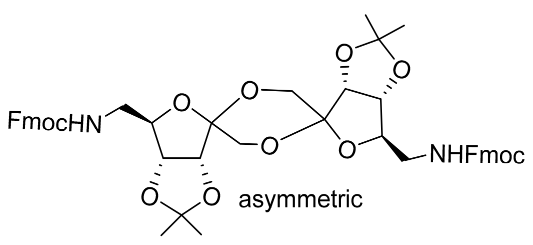

5ac, $CDCl_3$, 100 MHz

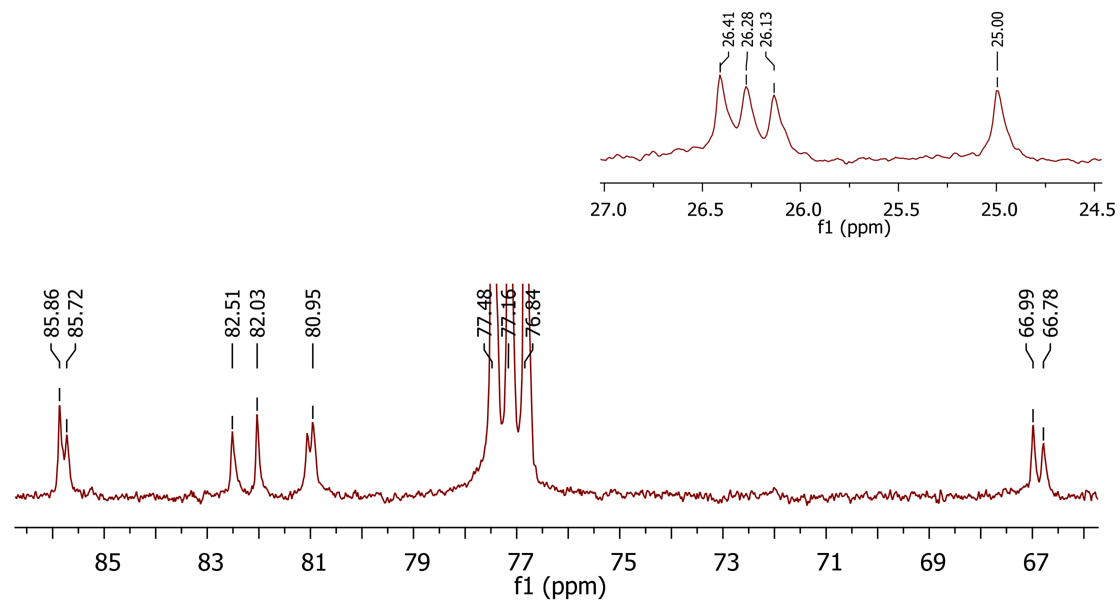

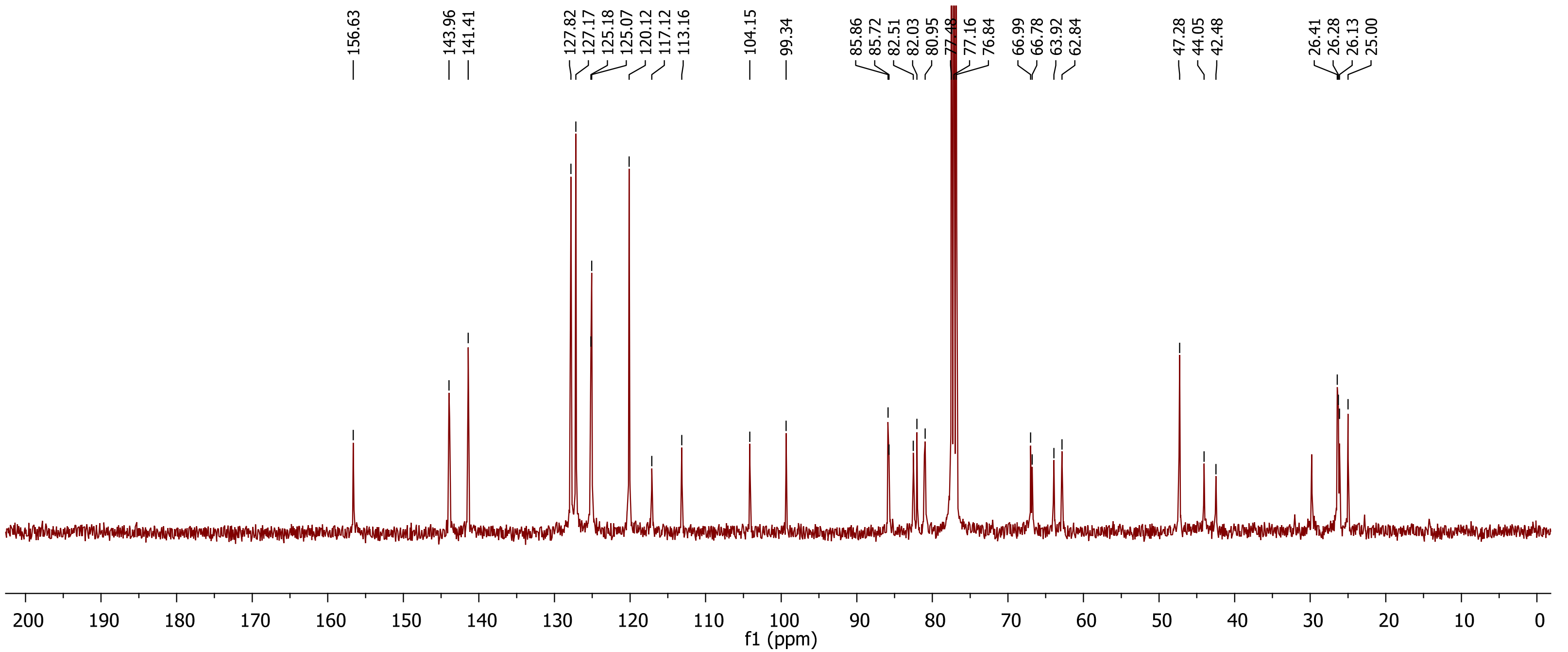

NOESY Spectra of **17a**

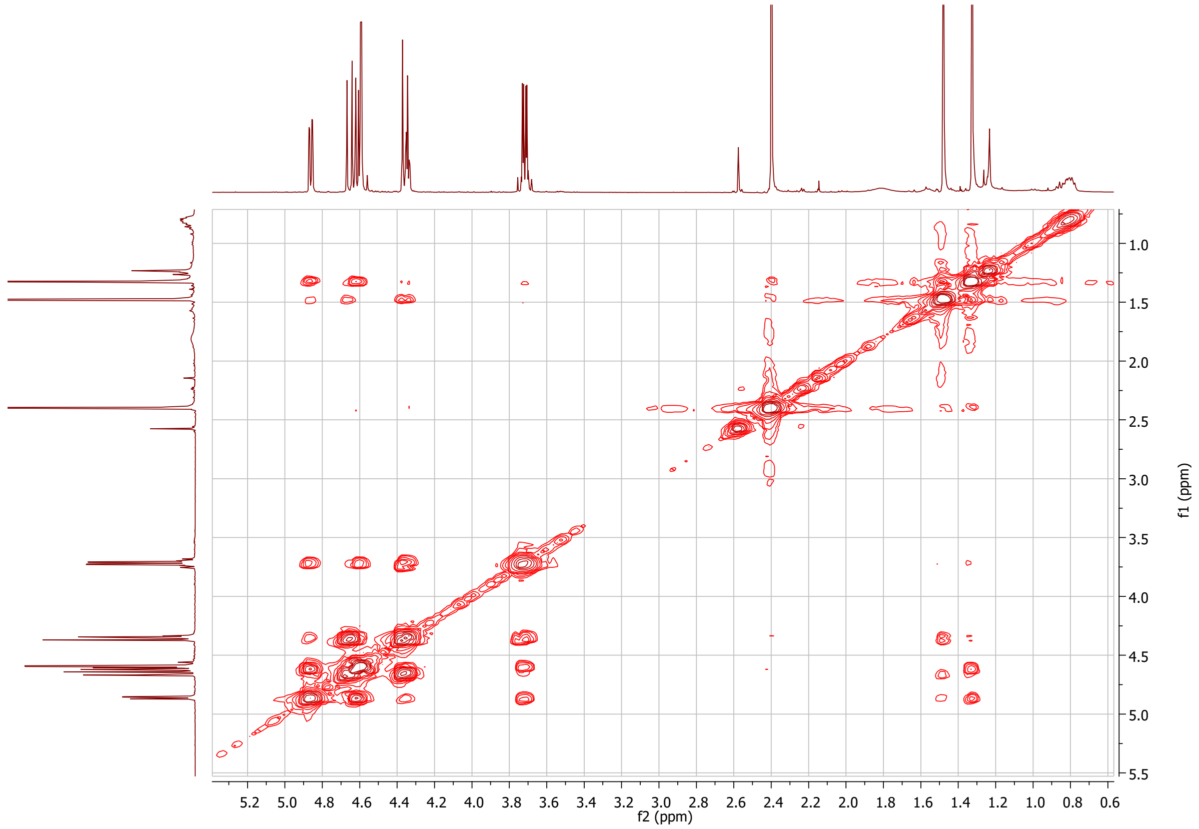

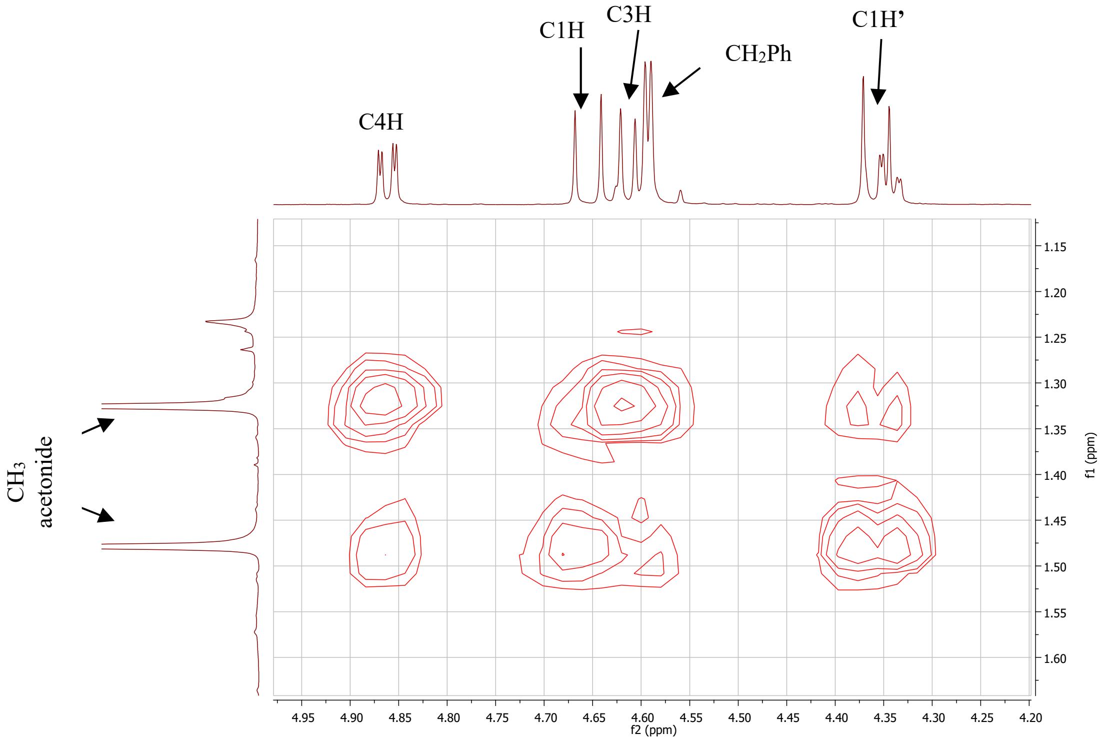

NOESY spectra of **11a**

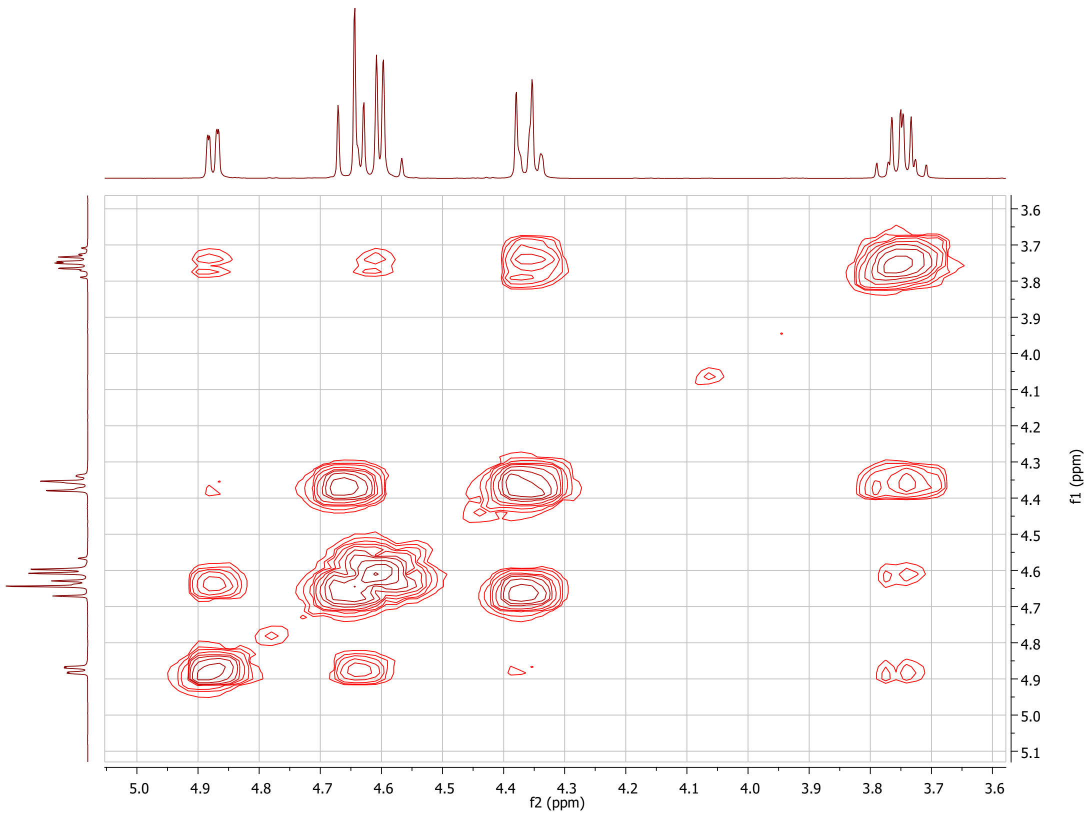

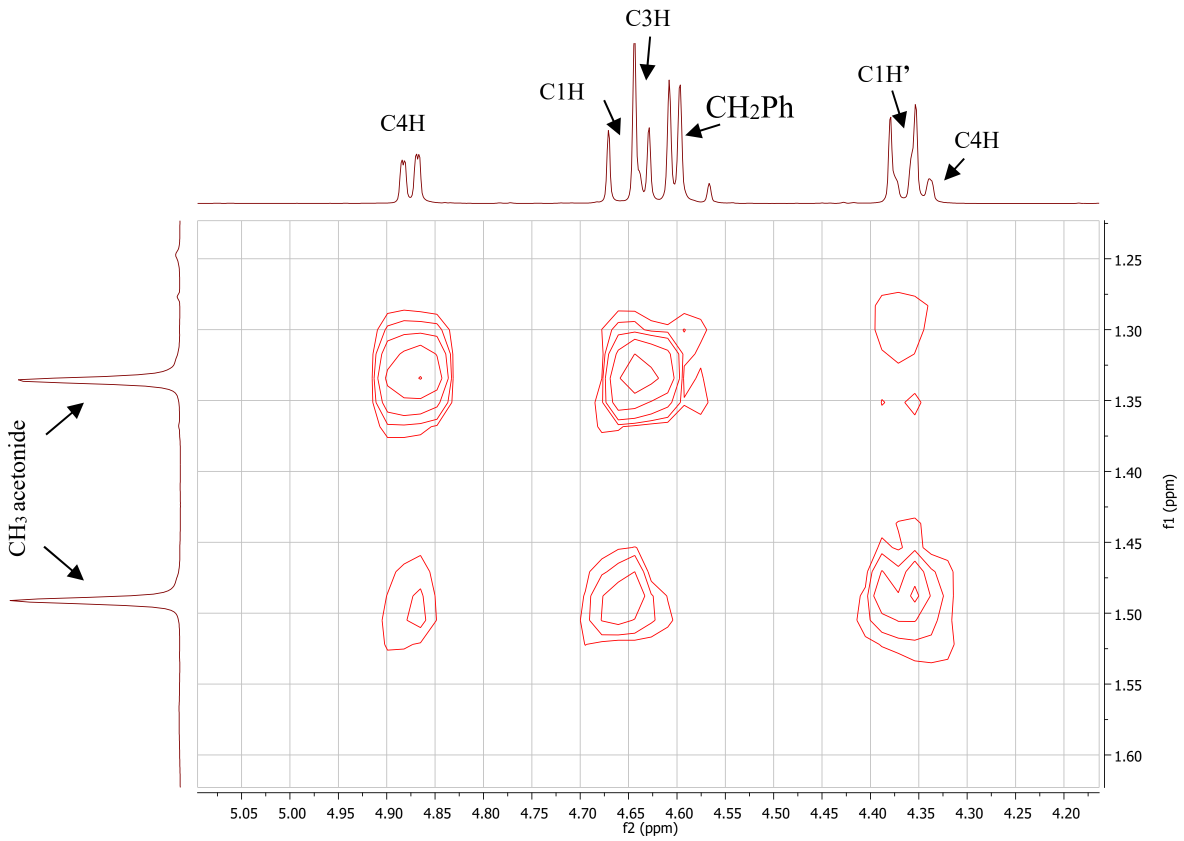

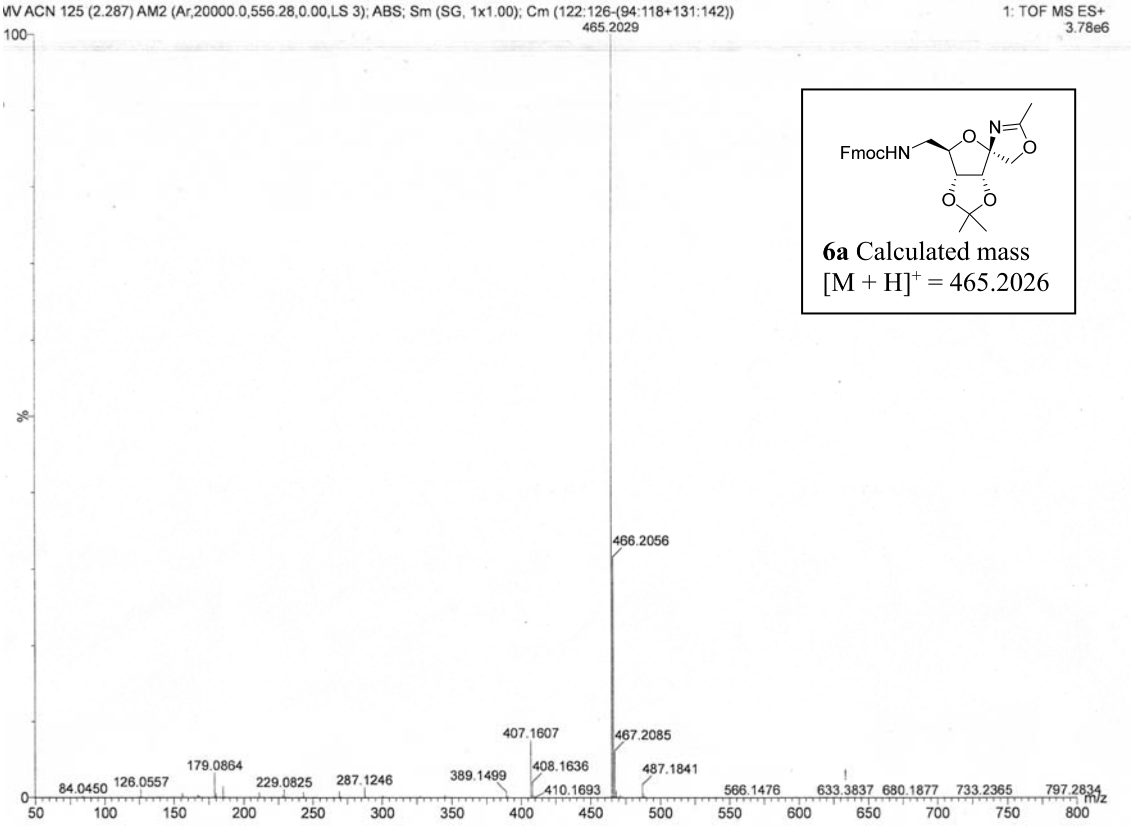

MV ACN 125 (2.287) AM2 (Ar,20000.0,556.28,0.00,LS 3); ABS; Sm (SG, 1x1.00); Cm (122:126-(94:118+131:142))
1: TOF MS ES+
3.78e6
100
%
0
465.2029
466.2056
407.1607
467.2085
408.1636
487.1841
410.1693
179.0864
84.0450
126.0557
229.0825
287.1246
389.1499
566.1476
633.3837
680.1877
733.2365
797.2834
m/z
50 100 150 200 250 300 350 400 450 500 550 600 650 700 750 800
FmocHN
N
O
O
O
O
6a Calculated mass
[M + H]+ = 465.2026

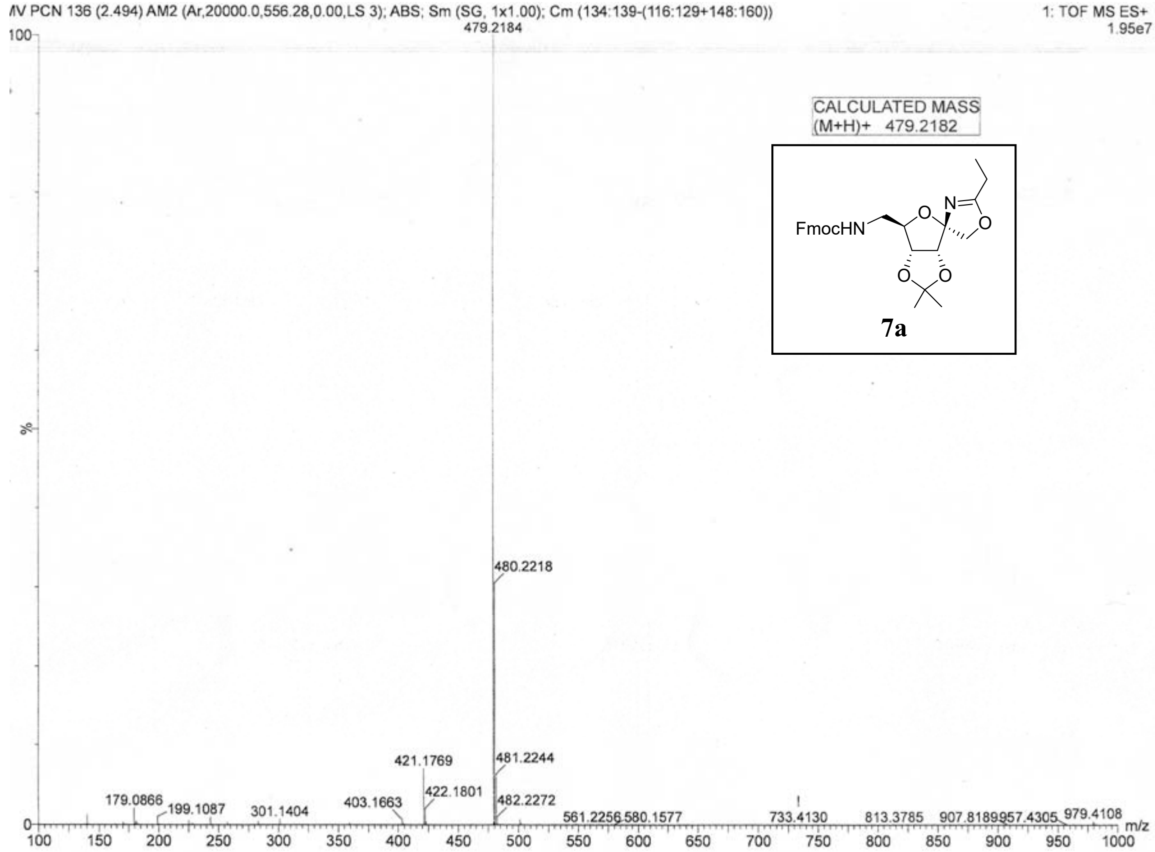

MV PCN 136 (2.494) AM2 (Ar,20000.0,556.28,0.00,LS 3); ABS; Sm (SG, 1x1.00); Cm (134:139-(116:129+148:160))
1: TOF MS ES+
1.95e7
100
%
0
479.2184
480.2218
421.1769
481.2244
422.1801
482.2272
179.0866
199.1087
301.1404
403.1663
561.2256
580.1577
733.4130
813.3785
907.8189
957.4305
979.4108
m/z
100 150 200 250 300 350 400 450 500 550 600 650 700 750 800 850 900 950 1000
CALCULATED MASS
(M+H)+ 479.2182
FmocHN
N
O
O
O
O
7a

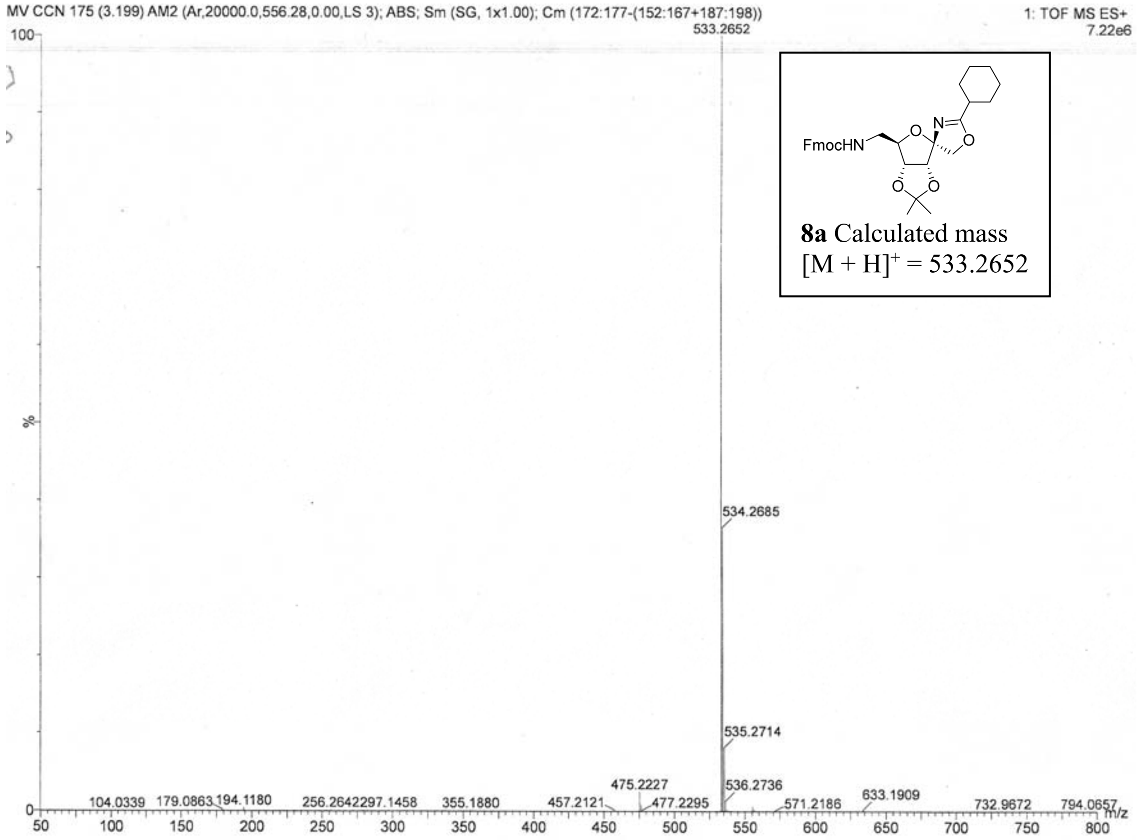
MV CCN 175 (3.199) AM2 (Ar,20000.0,556.28,0.00,LS 3); ABS; Sm (SG, 1x1.00); Cm (172:177-(152:167+187:198))
1: TOF MS ES+
7.22e6
533.2652
534.2685
535.2714
536.2736
475.2227
477.2295
457.2121
355.1880
256.2642297.1458
104.0339
179.0863194.1180
571.2186
633.1909
732.9672
794.0657
m/z
%
FmocHN
8a Calculated mass
[M + H]+ = 533.2652

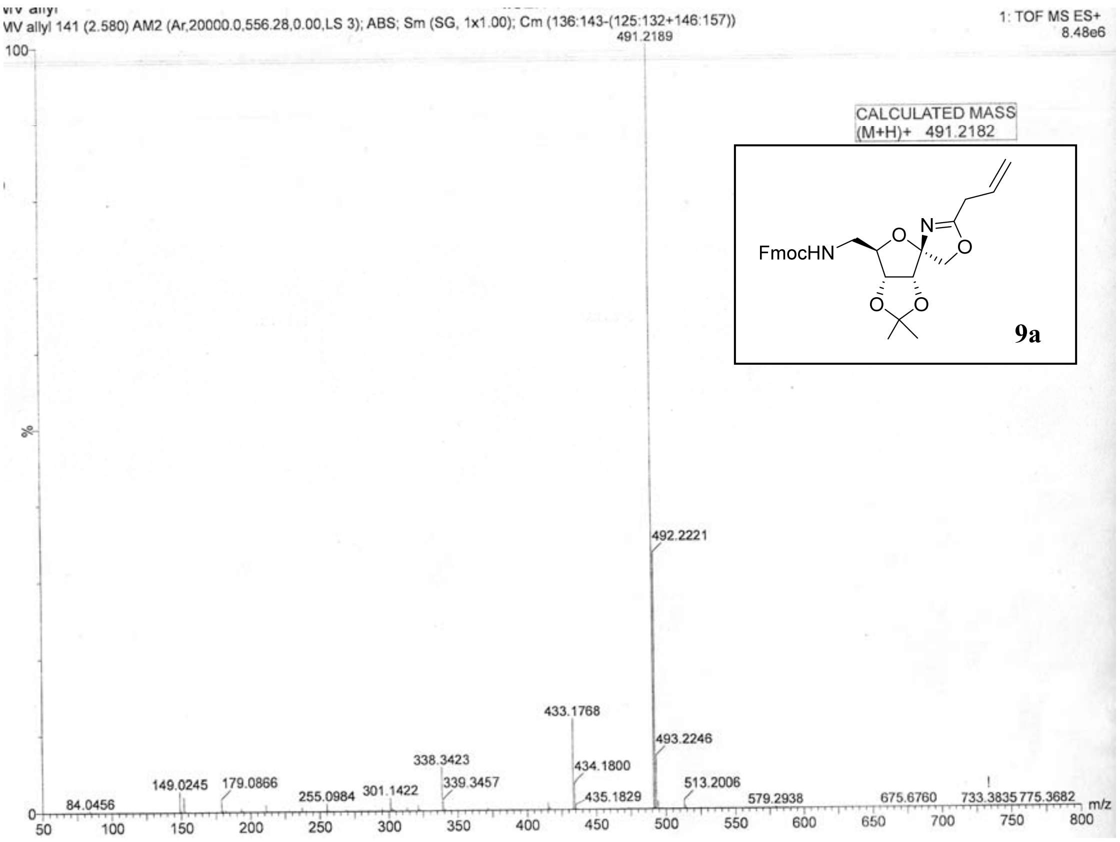
MV allyl 141 (2.580) AM2 (Ar,20000.0,556.28,0.00,LS 3); ABS; Sm (SG, 1x1.00); Cm (136:143-(125:132+146:157))
1: TOF MS ES+
8.48e6
491.2189
CALCULATED MASS
(M+H)+ 491.2182
492.2221
493.2246
433.1768
434.1800
435.1829
338.3423
339.3457
149.0245
179.0866
255.0984
301.1422
84.0456
513.2006
579.2938
675.6760
733.3835775.3682
m/z
%
FmocHN
9a

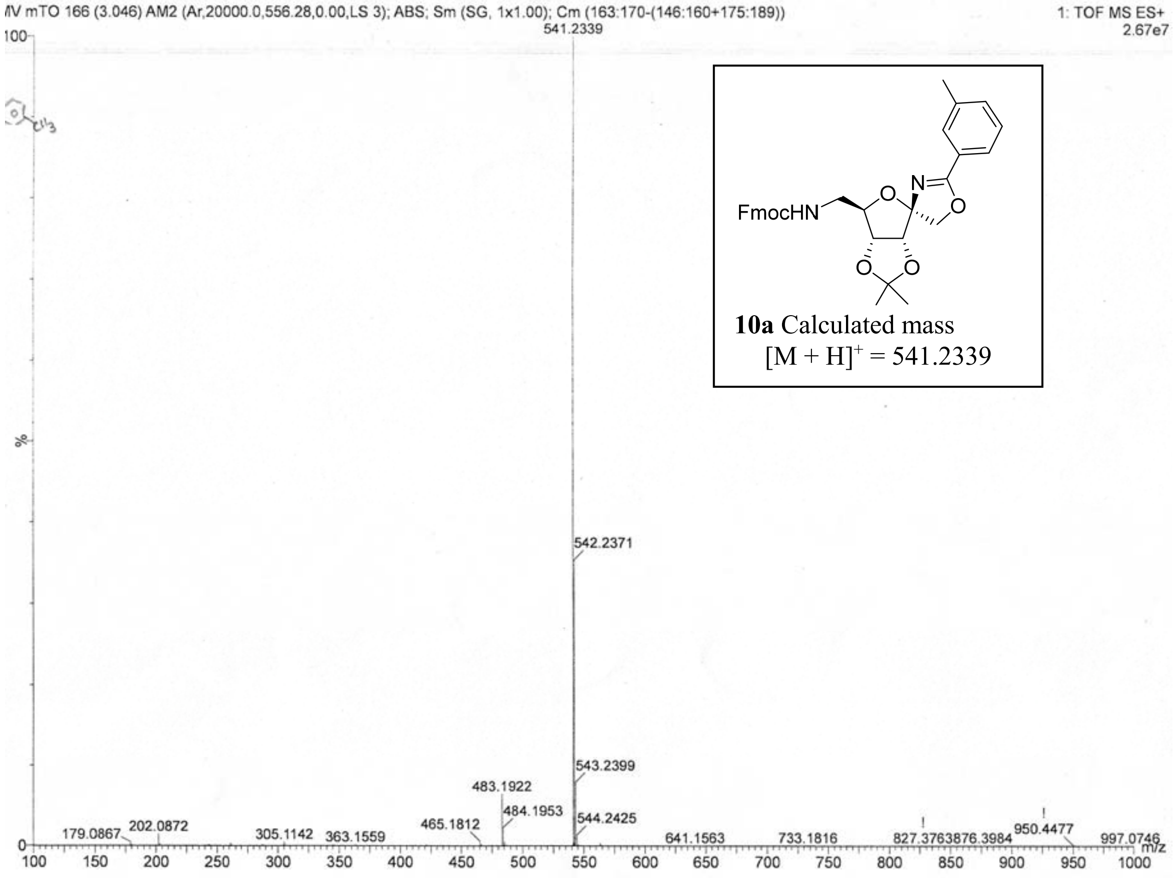
MV mTO 166 (3.046) AM2 (Ar,20000.0,556.28,0.00,LS 3); ABS; Sm (SG, 1x1.00); Cm (163:170-(146:160+175:189))
1: TOF MS ES+
2.67e7
541.2339
542.2371
543.2399
544.2425
483.1922
484.1953
465.1812
179.0867
202.0872
305.1142
363.1559
641.1563
733.1816
827.3763876.3984
950.4477
997.0746
m/z
%
FmocHN
10a Calculated mass
[M + H]+ = 541.2339

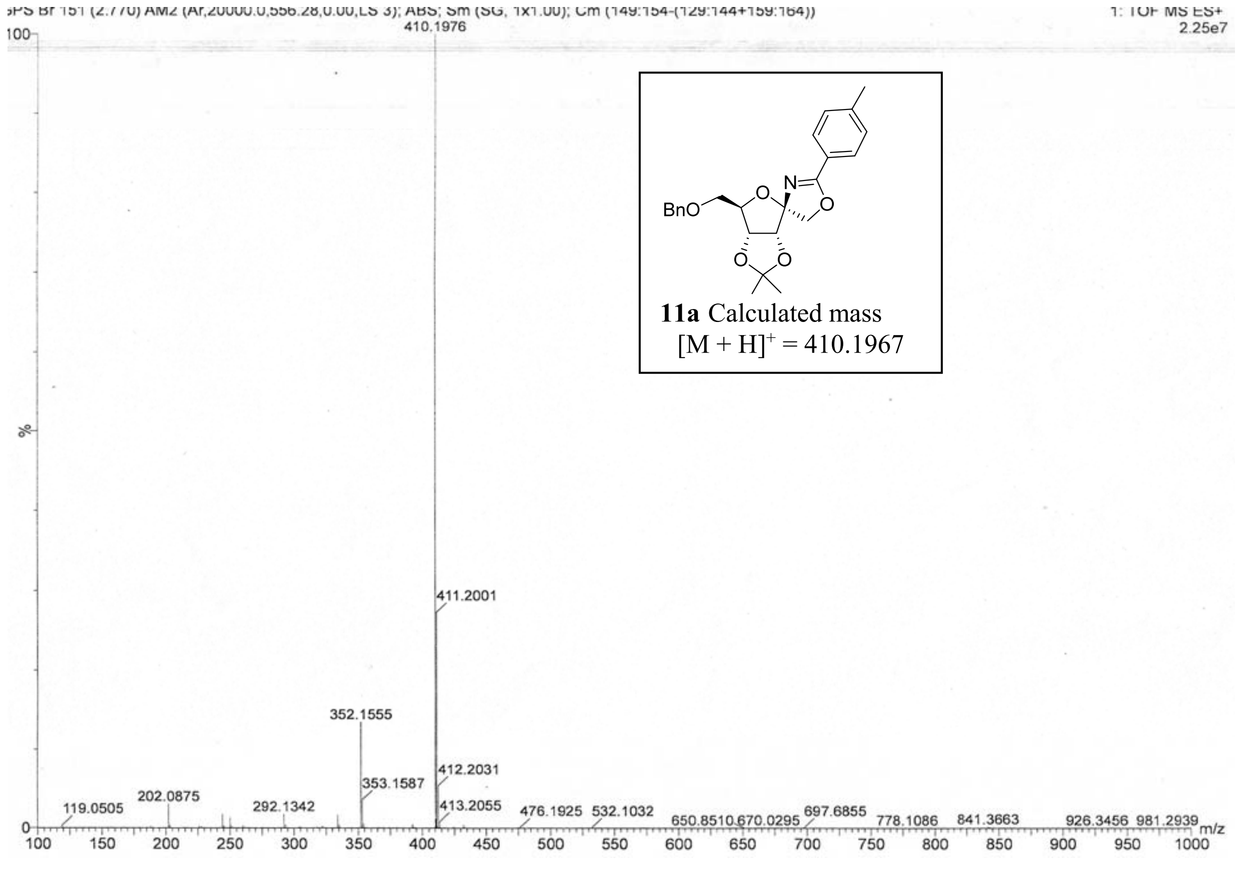
1: TOF MS ES+
2.25e7
410.1976
411.2001
412.2031
413.2055
352.1555
353.1587
119.0505
202.0875
292.1342
476.1925
532.1032
650.8510.670.0295
697.6855
778.1086
841.3663
926.3456
981.2939
m/z
%
BnO
11a Calculated mass
[M + H]+ = 410.1967

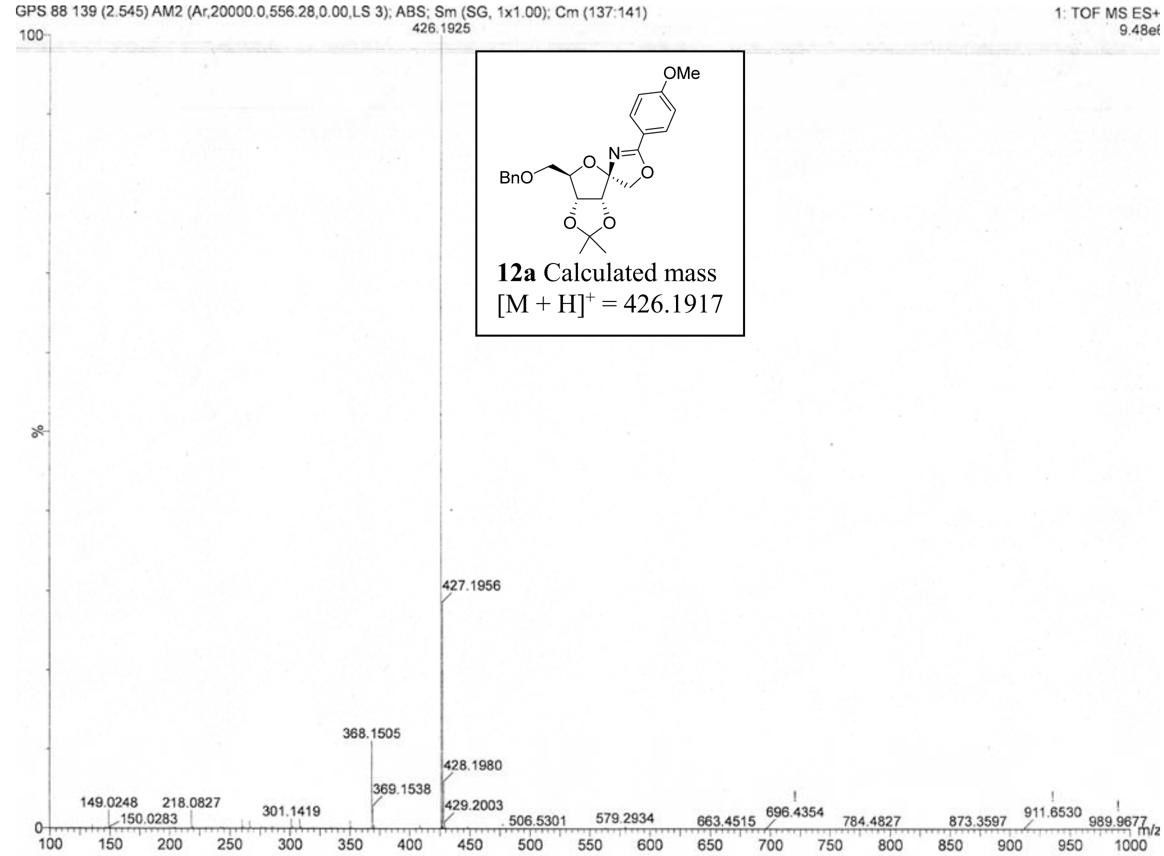
GPS 88 139 (2.545) AM2 (Ar,20000.0,556.28,0.00,LS 3); ABS; Sm (SG, 1x1.00); Cm (137:141)
1: TOF MS ES+
9.48e6
426.1925
OMe
BnO
N
O
12a Calculated mass
[M + H]+ = 426.1917
%
427.1956
368.1505
428.1980
369.1538
429.2003
149.0248
218.0827
150.0283
301.1419
506.5301
579.2934
663.4515
696.4354
784.4827
873.3597
911.6530
989.9677
m/z
100
150
200
250
300
350
400
450
500
550
600
650
700
750
800
850
900
950
1000

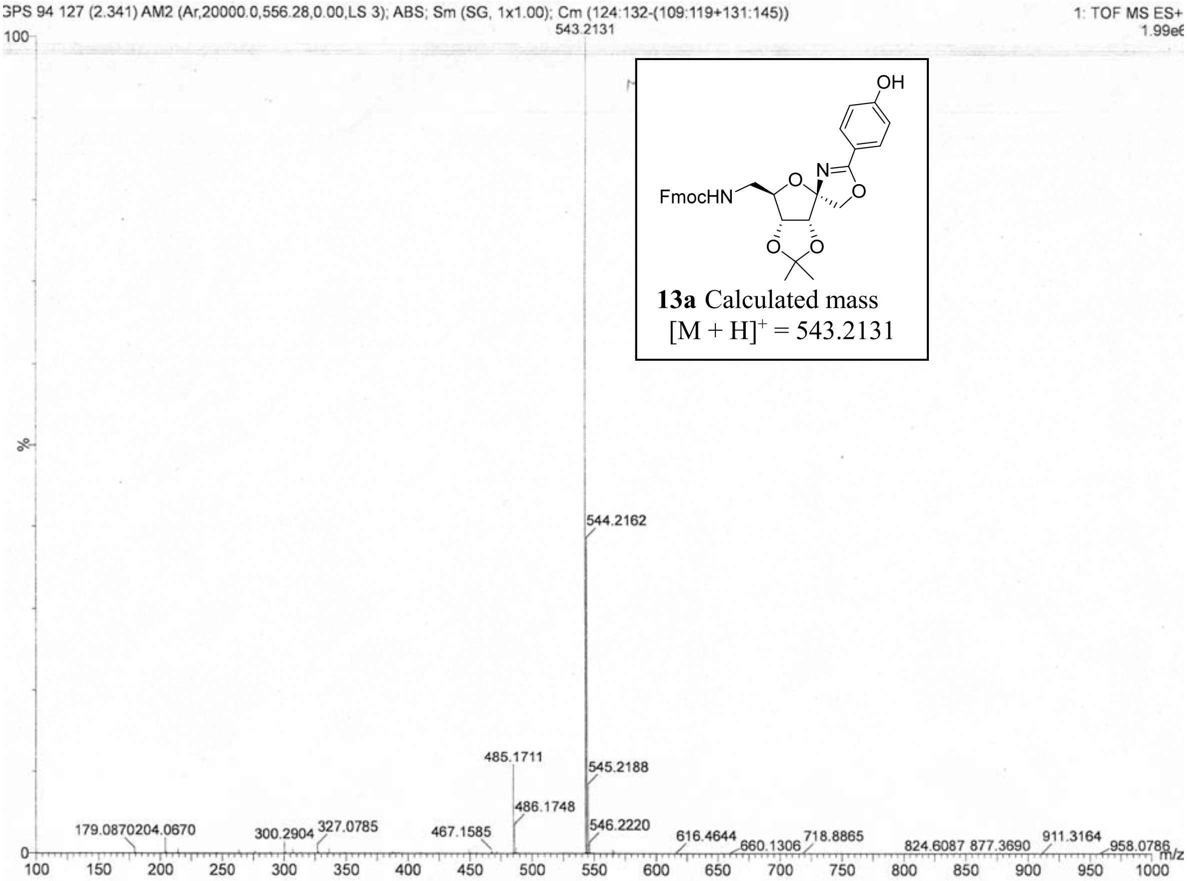
GPS 94 127 (2.341) AM2 (Ar,20000.0,556.28,0.00,LS 3); ABS; Sm (SG, 1x1.00); Cm (124:132-(109:119+131:145))
1: TOF MS ES+
1.99e6
543.2131
OH
FmocHN
N
O
13a Calculated mass
[M + H]+ = 543.2131
%
544.2162
485.1711
545.2188
486.1748
546.2220
179.0870
204.0670
300.2904
327.0785
467.1585
616.4644
660.1306
718.8865
824.6087
877.3690
911.3164
958.0786
m/z
100
150
200
250
300
350
400
450
500
550
600
650
700
750
800
850
900
950
1000

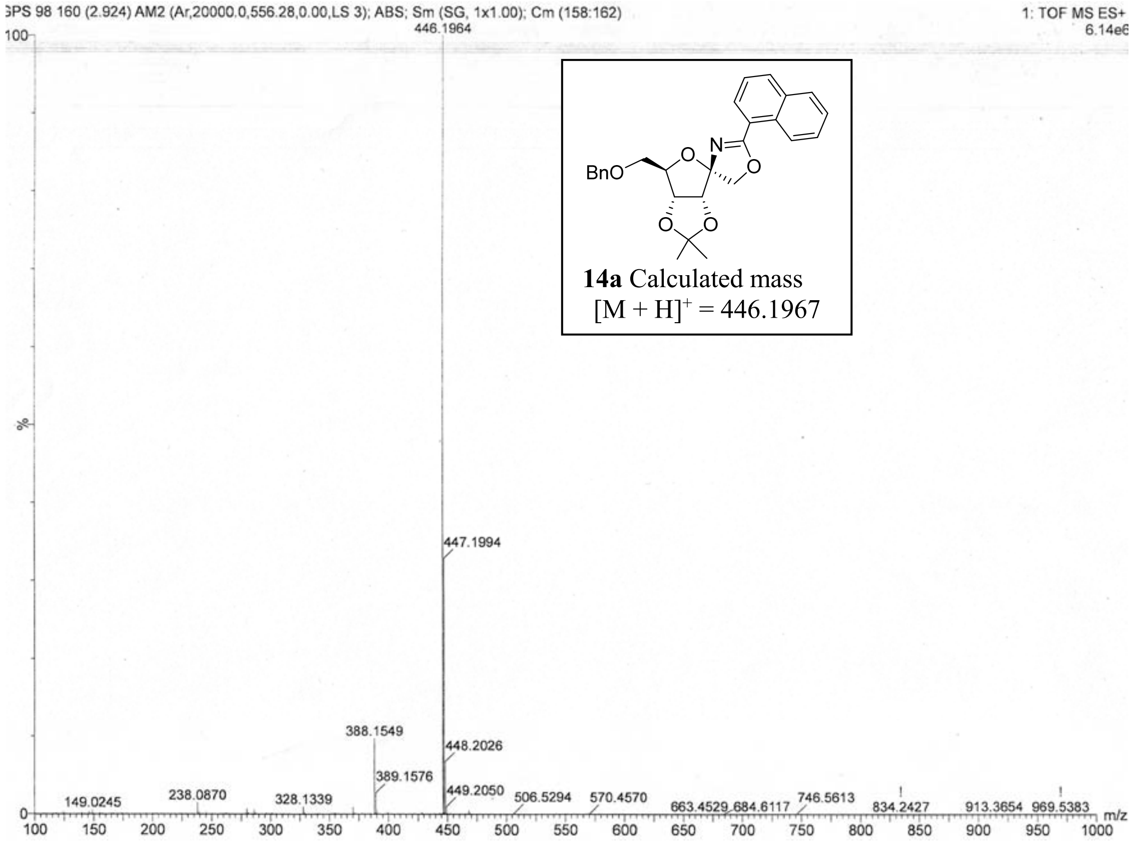
GPS 98 160 (2.924) AM2 (Ar,20000.0,556.28,0.00,LS 3); ABS; Sm (SG, 1x1.00); Cm (158:162)
1: TOF MS ES+
6.14e6
100
%
0
446.1964
447.1994
388.1549
448.2026
389.1576
449.2050
149.0245
238.0870
328.1339
506.5294
570.4570
663.4529
684.6117
746.5613
834.2427
913.3654
969.5383
m/z
BnO
O
N
O
O
O
14a Calculated mass
[M + H]+ = 446.1967

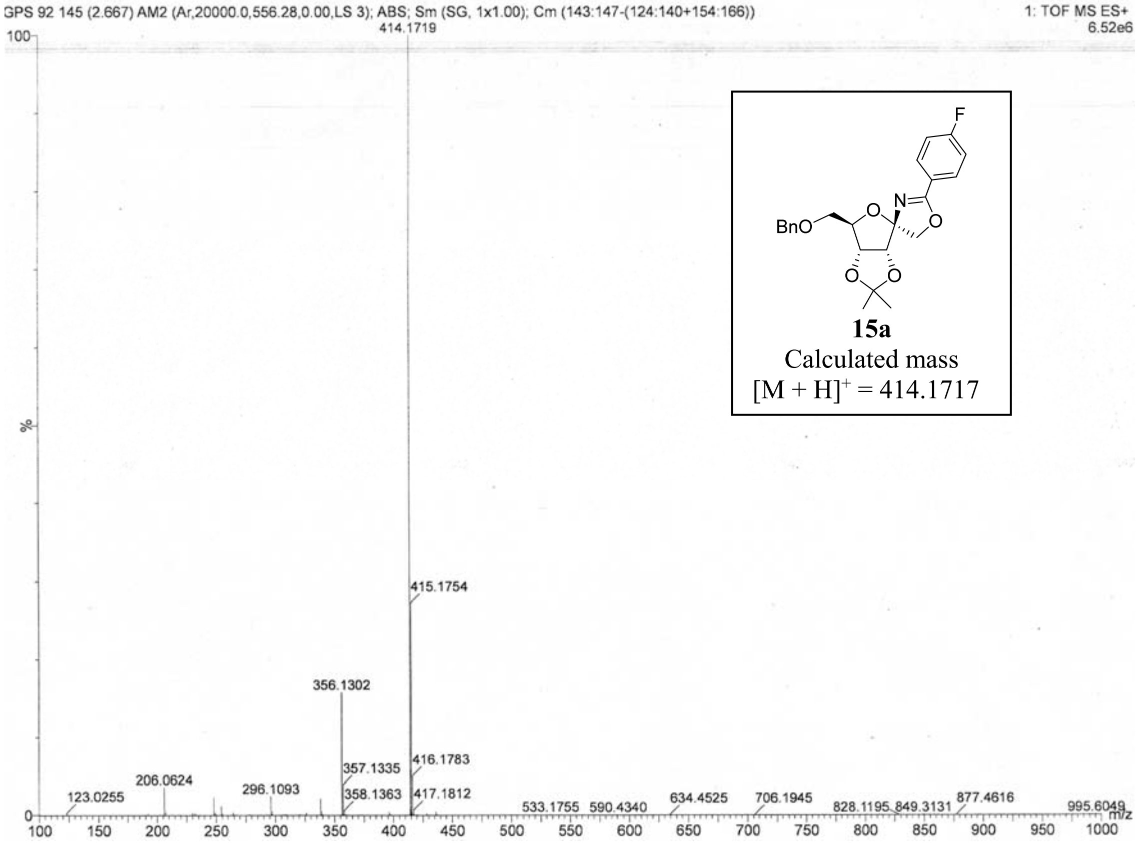
GPS 92 145 (2.667) AM2 (Ar,20000.0,556.28,0.00,LS 3); ABS; Sm (SG, 1x1.00); Cm (143:147-(124:140+154:166))
1: TOF MS ES+
6.52e6
100
%
0
414.1719
415.1754
356.1302
416.1783
357.1335
358.1363
417.1812
123.0255
206.0624
296.1093
533.1755
590.4340
634.4525
706.1945
828.1195
849.3131
877.4616
995.6049
m/z
F
BnO
O
N
O
O
O
15a
Calculated mass
[M + H]+ = 414.1717

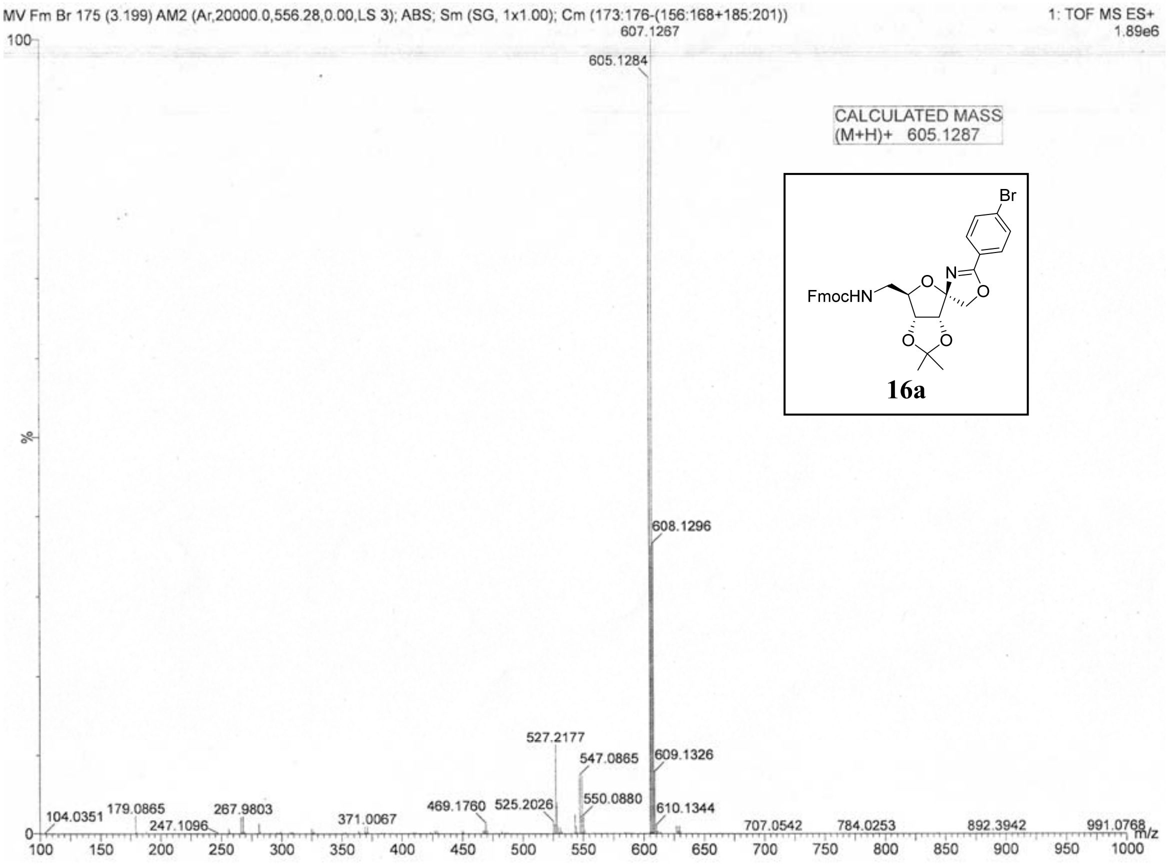

MV Fm Br 175 (3.199) AM2 (Ar,20000.0,556.28,0.00,LS 3); ABS; Sm (SG, 1x1.00); Cm (173:176-(156:168+185:201))
1: TOF MS ES+
1.89e6
607.1267
605.1284
608.1296
609.1326
610.1344
527.2177
547.0865
550.0880
525.2026
469.1760
371.0067
267.9803
247.1096
179.0865
104.0351
707.0542
784.0253
892.3942
991.0768
CALCULATED MASS
(M+H)+ 605.1287
FmocHN
Br
N
O
16a
%
m/z

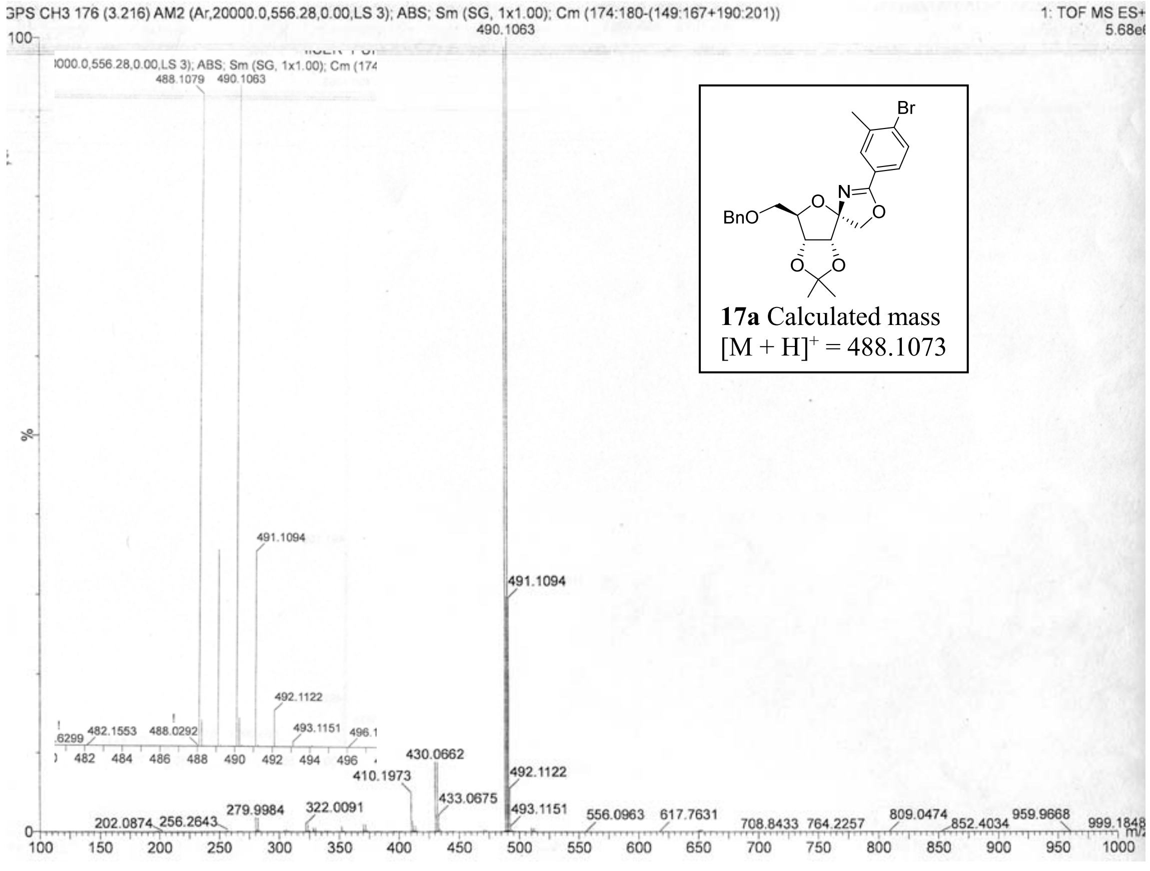

GPS CH3 176 (3.216) AM2 (Ar,20000.0,556.28,0.00,LS 3); ABS; Sm (SG, 1x1.00); Cm (174:180-(149:167+190:201))
1: TOF MS ES+
5.68e6
490.1063
488.1079
491.1094
492.1122
493.1151
488.0292
482.1553
430.0662
433.0675
410.1973
322.0091
279.9984
256.2643
202.0874
556.0963
617.7631
708.8433
764.2257
809.0474
852.4034
959.9668
999.1848
BnO
Br
N
O
17a Calculated mass
[M + H]+ = 488.1073
%

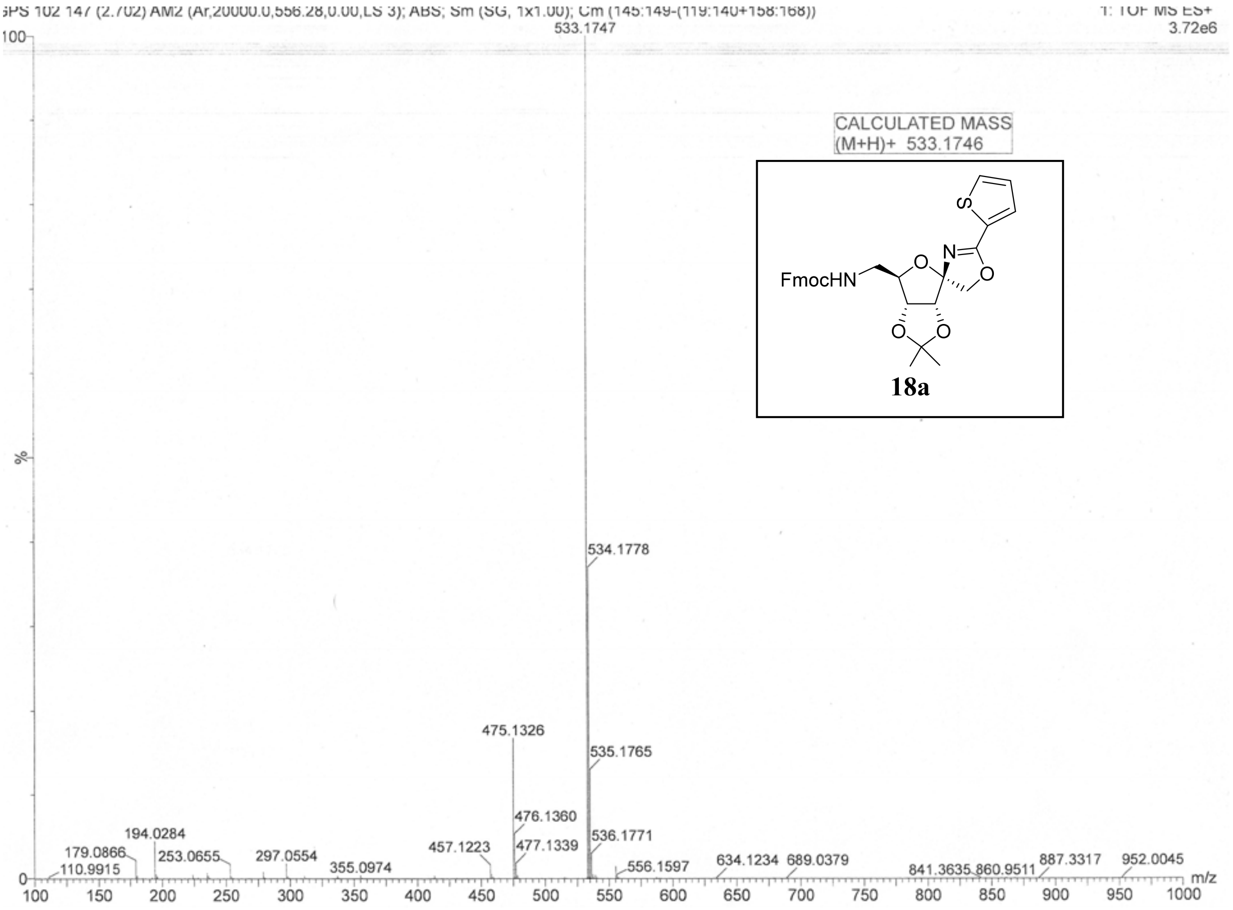
GPS 102 147 (2.702) AM2 (Ar,20000.0,556.28,0.00,LS 3); ABS; Sm (SG, 1x1.00); Cm (145:149-(119:140+158:168))
1: TOF MS ES+
3.72e6
533.1747
CALCULATED MASS
(M+H)+ 533.1746
FmocHN
18a
534.1778
475.1326
535.1765
476.1360
536.1771
477.1339
457.1223
194.0284
179.0866
110.9915
253.0655
297.0554
355.0974
556.1597
634.1234
689.0379
841.3635
860.9511
887.3317
952.0045
%
m/z

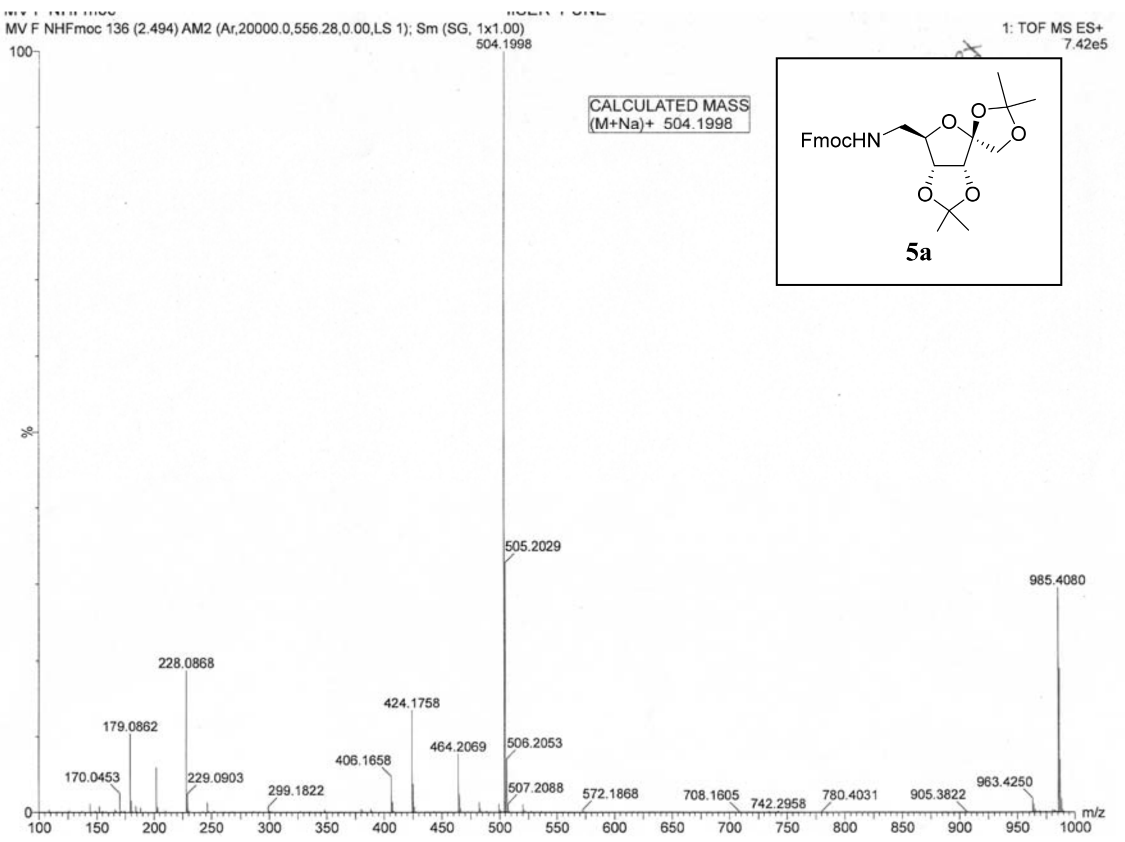
MV F NHFmoc 136 (2.494) AM2 (Ar,20000.0,556.28,0.00,LS 1); Sm (SG, 1x1.00)
1: TOF MS ES+
7.42e5
504.1998
CALCULATED MASS
(M+Na)+ 504.1998
FmocHN
5a
505.2029
985.4080
228.0868
424.1758
179.0862
464.2069
506.2053
406.1658
170.0453
229.0903
299.1822
507.2088
572.1868
708.1605
742.2958
780.4031
905.3822
963.4250
%
m/z

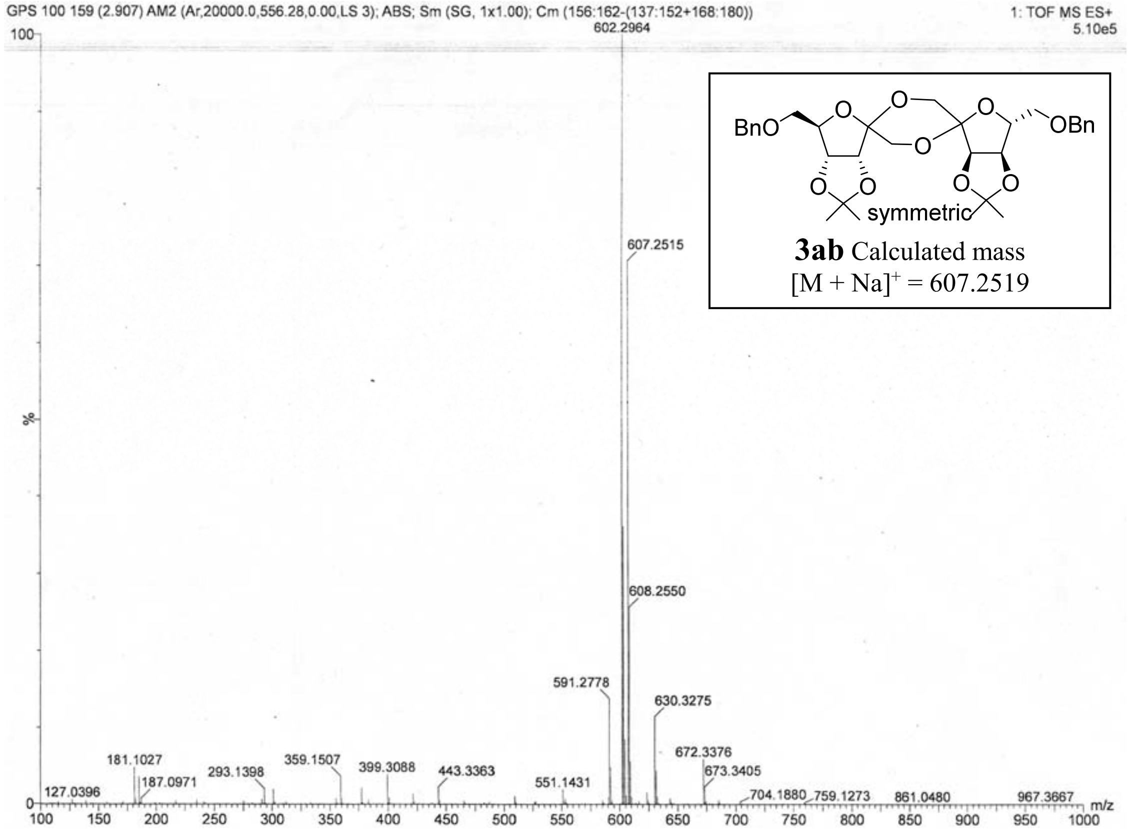
GPS 100 159 (2.907) AM2 (Ar,20000.0,556.28,0.00,LS 3); ABS; Sm (SG, 1x1.00); Cm (156:162-(137:152+168:180))
1: TOF MS ES+
5.10e5
602.2964
607.2515
608.2550
591.2778
630.3275
672.3376
673.3405
181.1027
187.0971
293.1398
359.1507
399.3088
443.3363
551.1431
127.0396
704.1880
759.1273
861.0480
967.3667
%
m/z
BnO
OBn
symmetric
3ab Calculated mass
[M + Na]+ = 607.2519

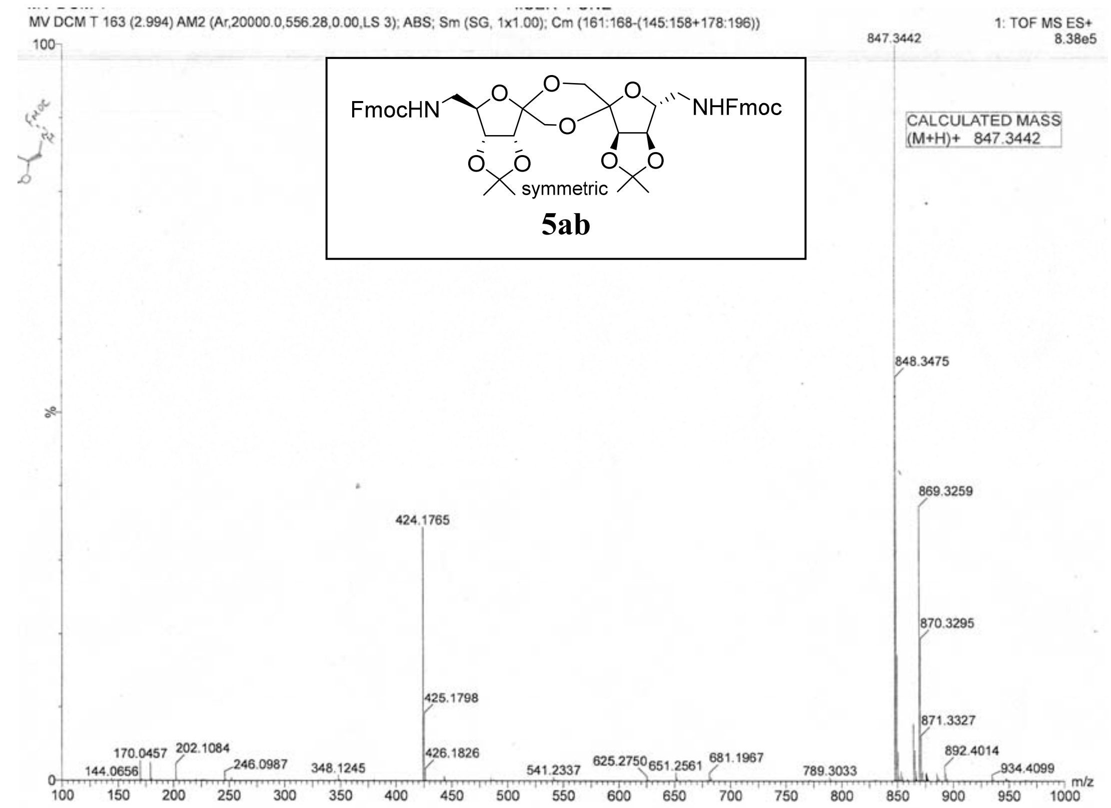
MV DCM T 163 (2.994) AM2 (Ar,20000.0,556.28,0.00,LS 3); ABS; Sm (SG, 1x1.00); Cm (161:168-(145:158+178:196))
1: TOF MS ES+
8.38e5
847.3442
CALCULATED MASS
(M+H)+ 847.3442
848.3475
869.3259
870.3295
871.3327
892.4014
934.4099
424.1765
425.1798
426.1826
170.0457
202.1084
144.0656
246.0987
348.1245
541.2337
625.2750
651.2561
681.1967
789.3033
%
m/z
FmocHN
NHFmoc
symmetric
5ab

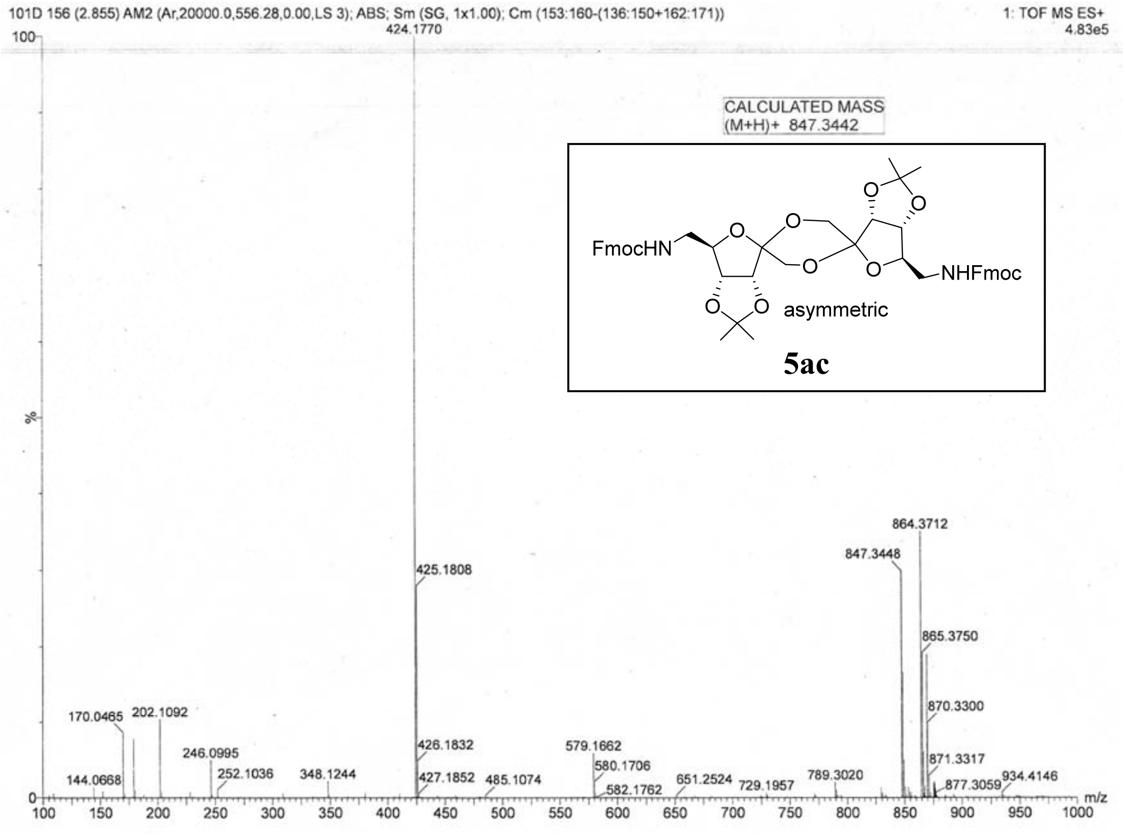

101D 156 (2.855) AM2 (Ar,20000.0,556.28,0.00,LS 3); ABS; Sm (SG, 1x1.00); Cm (153:160-(136:150+162:171))
1: TOF MS ES+
4.83e5
424.1770
CALCULATED MASS
(M+H)+ 847.3442
FmocHN
NHFmoc
asymmetric
5ac
%
864.3712
847.3448
425.1808
865.3750
870.3300
170.0465
202.1092
426.1832
579.1662
871.3317
246.0995
580.1706
144.0668
252.1036
348.1244
427.1852
485.1074
582.1762
651.2524
729.1957
789.3020
877.3059
934.4146
m/z
